# Dynamics of *bicoid* mRNA localisation and translation dictate morphogen gradient formation

**DOI:** 10.1038/s44318-026-00927-5

**Published:** 2026-10-10

**Authors:** Thamarailingam Athilingam, Elise L Wilby, Pierre Bensidoun, Antonio Trullo, Morgane Verbrugghe, Jérémy Dufourt, Xingzhu Shi, Mounia Lagha, Timothy E Saunders, Timothy T Weil

**Affiliations:** 1 Warwick Medical School, University of Warwick, Coventry, CV4 7AL UK; 2 Department of Zoology, University of Cambridge, Cambridge, CB2 3EJ UK; 3 Institut de Génétique Moléculaire de Montpellier, Univ Montpellier, CNRS, Montpellier, France; 4 Institut de Recherche en Infectiologie de Montpellier (IRIM), CNRS UMR 9004, Université de Montpellier, 1919 Route de Mende, 34293, Montpellier Cedex 5, France

**Keywords:** Development, RNA Biology

## Abstract

The transcription factor Bicoid (Bcd) protein guides early *Drosophila* patterning and is the best-characterised morphogen. The source of the morphogen, *bcd* mRNA, is maternally deposited during oogenesis and localised to the anterior pole. When and where Bcd protein is produced and how this protein gradient is dynamically shaped remains contentious. Here, we use the SunTag reporter system to quantitatively examine the spatiotemporal profile of *bcd* mRNA translation in vivo. We show that association with Processing bodies (P bodies) in mature oocytes prevent premature *bcd* mRNA translation. Following egg activation, *bcd* mRNA dissociates from P bodies and translation is observed at the anterior pole. Translation remains largely restricted to the anterior domain throughout early development. At cellularisation, translation ceases and the remaining *bcd* mRNA associates with reformed P bodies, which appear to block any further translation. We use these observations to create a new modified synthesis-diffusion-degradation model of Bcd gradient, which is consistent with experimental measurements. Overall, our study reveals that *bcd* mRNA translation is tightly controlled in space and time during oogenesis and early embryogenesis.

## Synopsis

Bicoid protein is a transcription factor that forms an essential morphogen gradient along the anterior-posterior body axis of the early *Drosophila* embryo. This study reveals for the first time the quantitative spatiotemporal dynamics of translation of a morphogen in vivo and presents a modified synthesis-diffusion-degradation model to explain the Bicoid gradient formation.

- *bicoid* mRNA is translationally silent while stored in P bodies during oogenesis, and the disruption of this association results in premature translation.
- P body disassembly at egg activation releases *bicoid* for translation, which is restricted to the vicinity of the anterior pole of the early embryo.
- *bicoid* mRNA translation discontinues at cellularisation and the remaining mRNA associates with reformed P bodies.
- Experimental measurement showing spatial and temporal variations in Bicoid protein production are consistent with a new synthesis-diffusion-degradation model.

## Abstract (web summary)

A synthesis-diffusion-degradation model based on the quantitative analysis of *bcd* mRNA dynamics in vivo explains morphogen gradient formation.

## Graphical abstract

**Figure:**
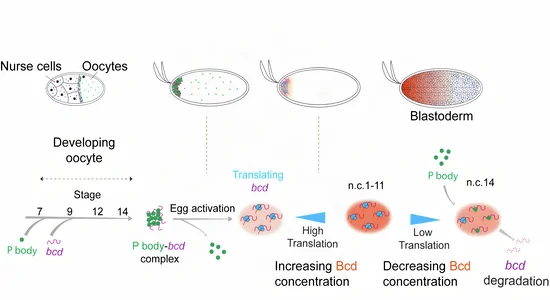

## Introduction

The concept of signalling molecules, coined morphogens, was proposed by Turing (1952). These molecules help guide spatial patterning by varying in concentration across developing systems. Downstream target genes respond in a concentration-dependent manner to the morphogen gradient (Ashe and Briscoe, 2006). Bicoid (Bcd) protein is a transcription factor found in the early *Drosophila* blastoderm stage embryo and was the first morphogen gradient identified (Driever and Nüsslein-Volhard, 1988). A concentration gradient of Bcd extends along the anterior-posterior (AP) axis from highest at the anterior pole of the embryo and reaches around 70% of the embryo’s length. The expression of target genes, like *hunchback*, depends on the local Bcd concentration (Gregor et al, 2007a; Rivera-Pomar et al, 1995; Struhl et al, 1989).

Shortly after its discovery, the Synthesis, Diffusion, Degradation (SDD) model was introduced to explain how the Bcd gradient forms (Driever and Nüsslein-Volhard, 1988; Gregor et al, 2007b). This model assumed that Bcd is produced at the anterior pole, then diffuses through the embryo and is subsequently degraded. The model predicted a concentration profile that decreased exponentially in steady state, consistent with in vivo observations (Gregor et al, 2007b; Houchmandzadeh et al, 2002; Wartlick et al, 2009). There are several mechanisms that can create morphogen profiles that decay exponentially (Durrieu et al, 2018; Huang and Saunders, 2020). Alternatives to the SDD model include *bcd* mRNA centric models with minimal or no diffusion of synthesised Bcd protein—(i) local production of Bcd protein by the spatially distributed *bcd* mRNA (Dalessi et al, 2012; Spirov et al, 2009); (ii) local production of Bcd protein by the diffusion of *bcd* mRNA (Dilao and Muraro, 2010); (iii) early translation of *bcd* in the oocyte which would allow the Bcd morphogen to spread quickly before patterning happens in later nuclear cycles (Ali-Murthy and Kornberg, 2016)—and models without degradation, with nuclear dilution determining local Bcd concentration (Coppey et al, 2007). These models predict different times and places for *bcd* mRNA translation. Still, they can all create exponential-like concentration gradients under the right conditions and time scales for patterning (Bergmann et al, 2007; Grimm et al, 2010; Lucas et al, 2013).

Localised *bcd* mRNA first appears at the anterior region in mid-stage oocytes (Johnston et al, 1989; St Johnston and Nüsslein-Volhard, 1992; Weil et al, 2006). As oogenesis progresses, *bcd* transitions from a continual active transport to stable anchoring at the anterior cortex (Weil et al, 2006; Weil et al, 2008). By the end of oogenesis, the mature oocyte (stage 14) is endowed with all the *bcd* needed for anterior patterning in the embryo (Johnston et al, 1989; St Johnston and Nüsslein-Volhard, 1992; Weil et al, 2006; Weil et al, 2008). There is no biochemical evidence that *bcd* translation happens before egg activation (Eichhorn et al, 2016; Kronja et al, 2014a; Kronja et al, 2014b), a conserved process that triggers several major changes in the egg (Horner and Wolfner, 2008; Hu and Wolfner, 2019; Mahowald et al, 1983; York-Andersen et al, 2015). These changes include restarting meiosis, altering the translational landscape, and reconfiguring the cytoskeleton. These events occur as the mature egg moves into the oviduct before fertilisation in the uterus (Aviles-Pagan and Orr-Weaver, 2018). Translational repression of *bcd* in the oocyte is proposed to occur through its association in a ribonuclear protein (RNP) granule, a class of biomolecular condensates (Sankaranarayanan et al, 2021; Weil et al, 2012b).

Most research on biomolecular condensate composition, physical properties, and function has focused on single-cell models and in vitro systems (Hubstenberger et al, 2017; Luo et al, 2018; Riggs et al, 2020; Standart and Weil, 2018; Xing et al, 2020). This provides an excellent basis for studying ribonucleoprotein (RNP) granule physiology and translational regulation in multicellular organisms. Genetic and physical manipulation in *Drosophila* have recently helped us understand the biological relevance of RNP granules in a developmental context (Bose et al, 2022; Buddika et al, 2022; Chen et al, 2024; Pushpalatha et al, 2022; Ramat et al, 2024). For example, visualising *nanos* mRNA translation shows that germ granules can either be sites of translation or repression depending on the location of the mRNA (Chen et al, 2024; Ramat et al, 2024). Also, the germ-line determinant *oskar* (*osk*) mRNA associates with “solid-like” condensates where its unique secondary structure is required to maintain *osk* mRNA inside the condensates, where it is translationally repressed (Bose et al, 2022).

A similar model of mRNA regulation via association with an RNP granule had previously been proposed for *bcd* mRNA (Sankaranarayanan et al, 2021; Weil et al, 2012b). Once localised to the anterior, *bcd* is sequestered in the core of Processing bodies (P bodies) away from ribosomes (Weil et al, 2012b). P bodies are an evolutionarily conserved cytoplasmic RNP granule. Depending on the cellular context, they are implicated in storing and degrading mRNA (Hubstenberger et al, 2017; Parker and Sheth, 2007). A variety of proteins that help regulate mRNA are enriched in P bodies including the DEAD-box family RNA helicase, Maternal Effect at 31B (Me31B). It has homologies with proteins in other species: DDX6 in humans, Dhh1 in *S.cerevisiae*, Xp54 in *Xenopus*, and Cgh-1 in *C.elegans* (Davidson et al, 2016; Weil et al, 2012b). It is known that Me31B functions both in repression and clearance of maternal transcripts in early *Drosophila* embryogenesis (Wang et al, 2017b).

Previous studies on the Bcd morphogen gradient have mainly looked at two areas. One focuses on the regulation of *bcd* mRNA during oogenesis (St Johnston, 2023; Trovisco et al, 2016; Weil et al, 2012b; Weil et al, 2010). The other examines Bcd protein dynamics after translation (Athilingam et al, 2024; Bergmann et al, 2007; Durrieu et al, 2018; Gregor et al, 2007b; Porcher et al, 2010). To fully understand how the Bcd gradient forms, we need to integrate these two approaches. Here, we used the SunTag/scFv imaging-based reporter system (Blake et al, 2024; Dufourt et al, 2021; Pichon et al, 2016; Tanenbaum et al, 2014; Vinter et al, 2021; Wang et al, 2016; Wu et al, 2016; Yan et al, 2016) to quantitatively assay *bcd* translation in *Drosophila* oocytes and embryos. We show that *bcd* mRNA is not translated when it is in P bodies at the end of oogenesis. Following egg activation, or experimentally driven disassociation, *bcd* is no longer in P bodies and is translated. We observed *bcd* translation starts quickly after release from P bodies, at the anterior pole of the embryo where *bcd* is localised. After cortical nuclear migration in the blastoderm, *bcd* localisation appears dispersed though the domains of translation remain unchanged. We saw a decrease in *bcd* translation in nuclear cycle (n.c.) 11 onwards, consistent with observations from assaying the protein levels (Little et al, 2011). Motivated by our experimental observations, we demonstrate that a modified SDD model, with spatially and temporally varying Bcd production, is consistent with our results and previous quantitative measurements of Bcd in the early embryo.

## Results

### *bcd* translation begins after egg activation

**Figure 1:**
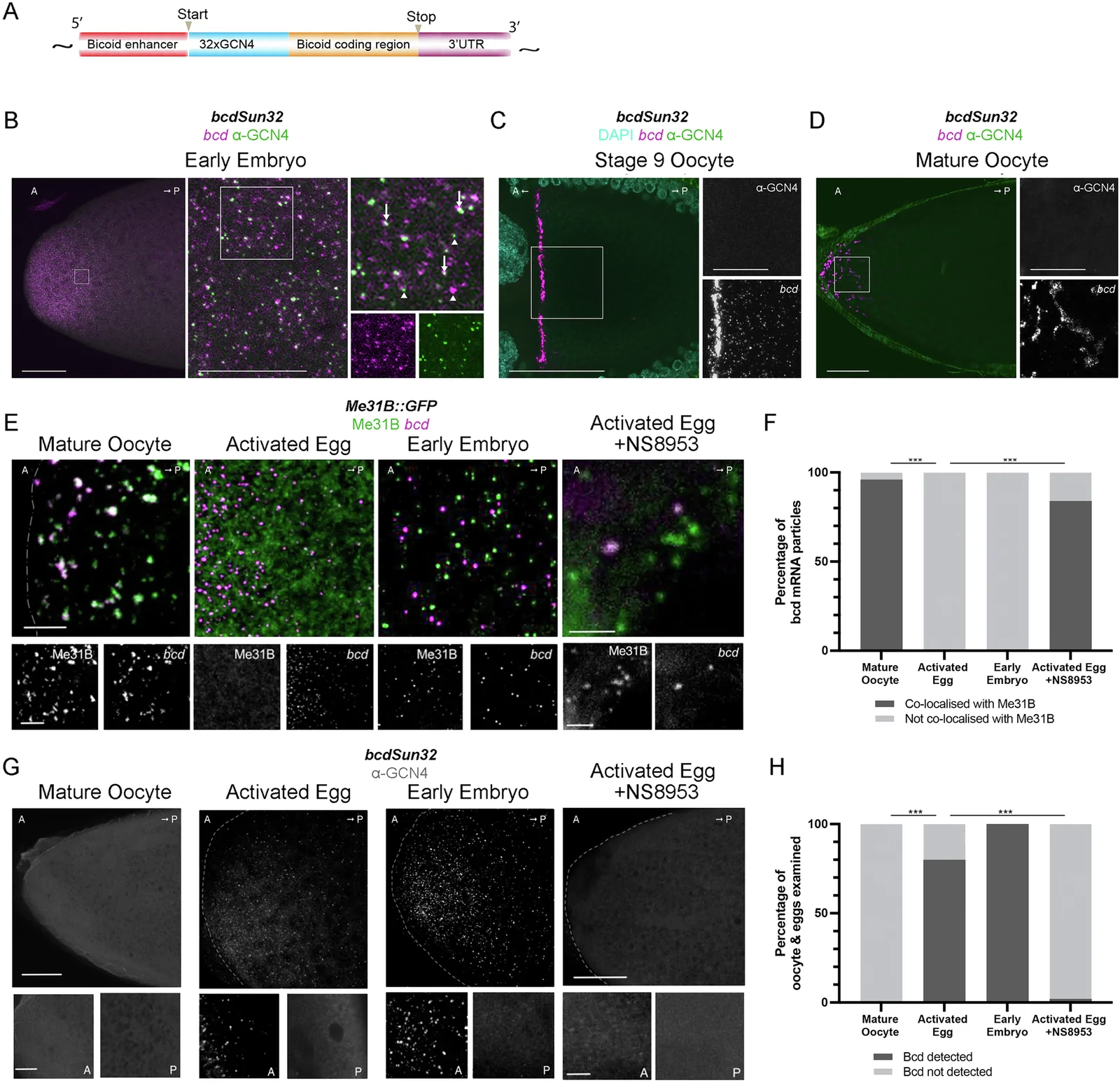
*bcdSun32* reporter enables visualisation of *bcd* mRNA translation. (**A**) Schematic illustrating the insertion of 32x SunTag repeats at the N-terminal of the *bcd* coding region after the start codon. (**B**) Representative image of *bcdSun32* in the early emryo (from 20 samples) with *bcd* translation detected by immunostaining for GCN4 (green) and *bcd* mRNA (magenta). Boxed region magnified on right. White arrowheads point to particles of either GCN4 or *bcd* mRNA alone. White arrows point to particles with GCN4 and *bcd* mRNA colocalized. Boxed region on right further magnified. *N* = 20. Scale bars: left 50 µm; middle 10 µm; right 3 µm. (**C**, **D**) Representative fixed 5–10 µm Z-stack images showing *bcdSun32* labelled *bcd* (magenta, smFISH) localising to the anterior margin of stage 9 and 14 (mature) oocytes. SunTag labelled Bcd protein (green, anti-GCN4) is not detected in stage 9 or 14 oocytes. *N* = 20. Scale bars: 50 µm; zoom 10 µm. (**E**) Representative fixed 10 µm Z-stack images (from 10 samples) showing Me31B::GFP (green, anti-GFP-nanobody) and *bcd* (magenta, smFISH) at the anterior in mature oocyte, in vitro activated egg, early embryo 30-min post fertilisation, and in vitro activated egg incubated with NS8953, a calcium channel blocker. Single-channel images are shown for Me31B and *bcd*. Scale bar: 5 μm. (**F**) Quantification of the colocalisation (as shown in E) between *bcd* particles and Me31B::GFP particles. *N* = 6 for mature oocytes, in vitro activated egg, early embryos. *N* = 3 for in vitro activated egg incubated with NS8953 due to difficulty with particle detection post oocyte swelling. *p<0.001*  (mature v.s. activated); *p* *<0.001* (activated v.s. NS8593). (**G**) Representative fixed 10 µm Z-stack images (from 10 samples) showing BcdSun32 protein (anti-GCN4) is only present at the anterior of an in vitro activated egg or early embryo 30-min post fertilisation. BcdSun32 protein is not detected in these samples at the posterior pole (image contrast increased to highlight the lack of distinct particles at the posterior). BcdSun32 protein is also not detected at the anterior or posterior of a mature oocyte or an in vitro activated egg incubated with NS8953 (images have the contrast increased to highlight the lack of distinct particles). Scale bar: 20 μm; zoom 2 μm. (**H**) Quantification of the number of mature oocytes or early embryos that displayed *bcd* translation as measured by the presence of BcdSun32 protein at the anterior but not the posterior. *N* = 10 for each condition. Anterior to the left and posterior to the right in all images. ***p < 0.001.

**Figure EV1:**
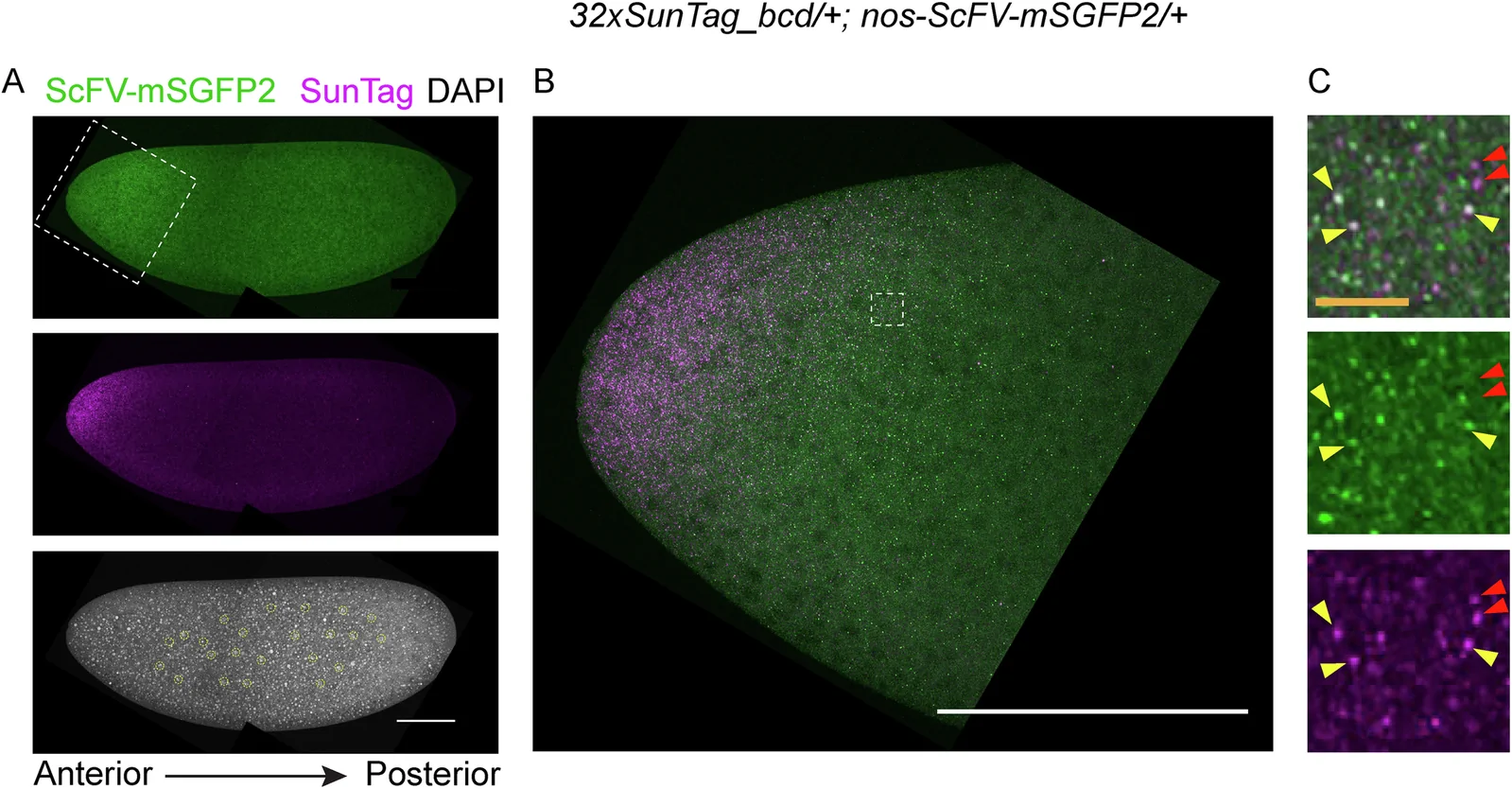
(Related to Fig. 1): Detection of *bcd* translation using nos-ScFv-mSGFP2. (**A**) Fixed n.c.4 *32xSunTag bcd*/*nos-ScFv-mSGFP2* embryos detecting colocalised and non-colocalised ScFv to *bcd*. (Top) ScFv-mSGFP2 (Green), (middle) *bcd* (Magenta), and (lower) Dapi (Grey). (**B**) Magnification of the anterior domain from (**A**). (**C**) Further magnified imaged showing the translating (yellow arrowheads) and non-translating (red arrowheads) *bcd* particles. Scale bars: (**A**) = 100 μm; (**B**) = 100 μm; (**C**) = 5 μm.

To test when the *bcd* mRNA is translated, we generated a *SunTag-bcd* transgene, containing an array of 32 SunTag GCN4 repeats (*bcdSun32*) (Fig. 1A, Methods). We first validated our reporter in early embryos, where Bcd protein is known to be present (Gregor et al, 2007b). We performed fluorescence single molecule in situ hybridisation (smFISH) to label *bcdSun32* and antibody labelling to visualise the SunTag nascent peptide simultaneously (Fig. 1B). We verified the *bcdSun32* reporter by showing two populations of *bcdSun32* particles: *bcdSun32* mRNA particles colocalising with the GCN4 antibody signal, (implying polysome and mRNA engaged in translation); and *bcdSun32* mRNA molecules that do not overlap with anti-GCN4 (not in translation) (Fig. 1B). We also performed this validation using the fluorescent detector, the single-chain antibody scFV, which is genetically encoded and maternally provided (*nos-scFv-mSGFP2*) (Fig. EV1) (Bellec et al, 2024). To further test the reporter, we expressed *bcdSun32* in oocytes. We observed no translation in oocytes (Fig. 1C,D) which matches what we predicted from earlier findings of *bcd* mRNA translational regulation (Lasko, 2012; Tadros and Lipshitz, 2009).

Previous work on P bodies has shown they do not contain ribosomes and can act as sites of mRNA storage (Hubstenberger et al, 2017; Riggs et al, 2020; Xing et al, 2020). To test if *bcd* mRNA is translationally repressed when in P bodies, we calculated the amount of *bcd* mRNA colocalisation to P bodies. We focused on the developmental transition from the mature egg to the early embryo. Our previous qualitative data postulated that location in P bodies is key for controlling *bcd* translation (Sankaranarayanan et al, 2021). Using single-molecule fluorescence in situ hybridisation (smFISH) and the standard P body marker Me31B, we confirmed that *bcd* is in P bodies before egg activation (First panel, Fig. 1E,F). Then, during the transition from a mature oocyte to activated egg, P bodies disassembled and *bcd* was no longer colocalised with P bodies (Second panel, Fig. 1E,F) (Sankaranarayanan et al, 2021; Weil et al, 2012b). We also confirmed that *bcd* is released from the anterior cortex at egg activation (Second panel, Fig. 1E) (Sankaranarayanan et al, 2021). In the early embryo, the distribution of *bcd* stayed the same while ‘embryonic’ P bodies formed (Third panel, Arrow heads, Fig. 1E). There was no colocalisation detected between *bcd* and P bodies at this stage, consistent with past work showing that the re-formed P bodies have different traits compared to those in the oocyte (Fig. 1F) (Sankaranarayanan et al, 2021).

We next sought to maintain the relationship between *bcd* mRNA and P bodies through egg activation. This would act as a control to further test if colocalisation of *bcd* to P bodies was necessary for its translational repression. Previous work has shown that a calcium wave is required at egg activation for further development (Hu and Wolfner, 2019; Kaneuchi et al, 2015; York-Andersen et al, 2020). Chemical treatment with NS8593 disrupts this calcium wave, while other phenotypic markers of egg activation are still observed (58). Using NS8593 to disrupt the calcium wave in the activated egg, we show P bodies are retained during ex vivo egg activation (Fig. 1E). In these treated eggs, *bcd* mRNA remains colocalised with the retained P bodies (Fig. 1E (Sankaranarayanan et al, 2021; Weil et al, 2012b)), we hypothesised that the loss of colocalisation between *bcd* and P bodies correlates with *bcd* translation.

To test this model, we used the *bcdSun32* to observe Bcd protein during this transition. Before egg activation, Bcd protein was not detected (Fig. 1G,H). Consistent with previous findings, we only observed the presence of Bcd at the anterior of the activated egg and early embryo (Fig. 1G,H) (Driever and Nüsslein-Volhard, 1988). Furthermore, we did not see Bcd in NS8593 treated activated mature eggs (Fig. 1G,H). This means that the P bodies likely serve as a barrier, keeping *bcd* transcripts within a translation-free environment in the oocyte.

### Premature release of *bcd* mRNA results in ectopic translation

To further show that *bcd* storage in P bodies is required for translational repression, we treated mature eggs with chemicals known to disrupt RNP granule integrity (Bose et al, 2022; Bose et al, 2024; Sankaranarayanan et al, 2021; Alberti et al, 2019; Gao et al, 2022; McSwiggen et al, 2019). Previous work has shown that the physical properties of P bodies in mature *Drosophila* oocytes can be shifted from an arrested to a more liquid-like state by the addition of the aliphatic alcohol hexanediol (HD) (Kroschwald et al, 2017; Ribbeck and Görlich, 2002; Sankaranarayanan et al, 2021). While 1,6 HD has been widely used to probe the physical state of phase-separated condensates both in vivo and  in vitro (Alberti et al, 2019; Gao et al, 2022; McSwiggen et al, 2019), in some cells it appears to have unwanted cellular consequences (Ulianov et al, 2021). These include a potentially lethal cellular consequences that may indirectly affect the ability of condensates to form (Kroschwald et al, 2017) and wider cellular implications thought to alter the activity of kinases (Duster et al, 2021). While we did not observe any noticeable cellular issues in mature *Drosophila* oocytes with 1,6 HD, we also used 2,5 HD, known to be less problematic in most tissues (Ulianov et al, 2021) and the monovalent salt sodium chloride (NaCl), which changes electrostatic interactions (Sankaranarayanan et al, 2021).

**Figure 2:**
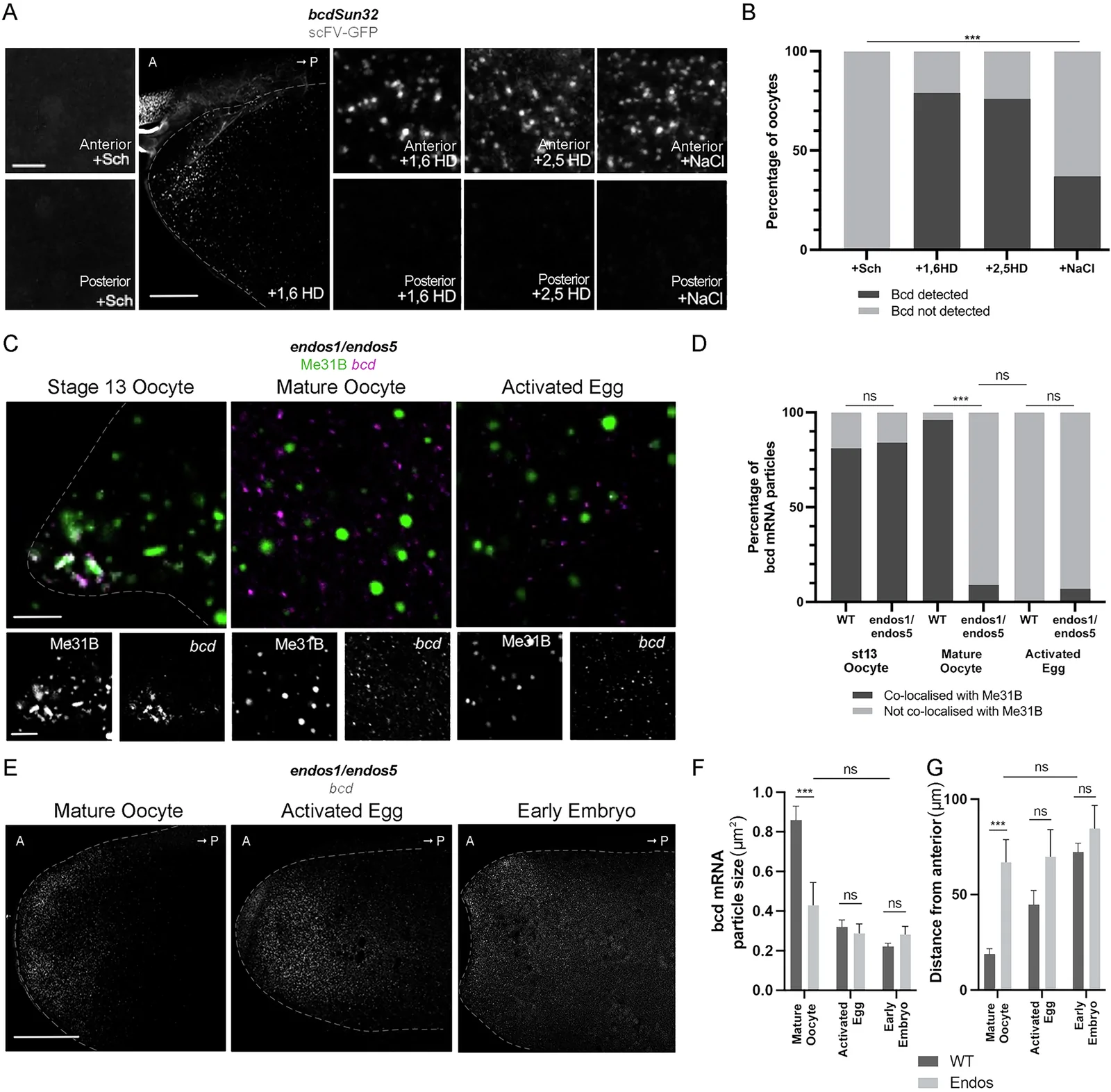
Chemical and genetic manipulation of P bodies can cause *bcd* mRNA misregulation. (**A**) Representative fixed 10 µm Z-stack image (from 10 samples) showing the overall distribution of BcdSun32 protein (scFV-GFP, anti-GFP-nanobody) at the anterior of stage 14 (mature) oocytes after treatment with different reagents for 20 min. Schneider’s Insect Medium ( + Sch) used as a control. Panels show that BcdSun32 protein is detected at the anterior pole and not at the posterior pole in 5% 1,6 hexanediol, 5% 2,5 hexanediol, and 400 mM NaCl treated mature oocytes. Scale bars: Left 20 μm; right 2 μm. (**B**) Quantification of experiments shown in (**A**). The number of oocytes that displayed Bcd protein at the anterior as measured by the presence of BcdSun32 at the anterior of the oocyte, but not the posterior. Schneider’s Insect Medium ( + Sch) used as a control. *N* = 30 oocytes for each treatment. Scale bar: 5 μm, *p<0.0001* . (**C**) Representative fixed single plane image showing the distribution of Me31B::GFP (green, anti-GFP-nanobody) and *bcd* (magenta, smFISH) in an Endos mutant stage 13 oocyte, mature oocyte, and an in vitro activated egg. Scale bar: 5 μm. (**D**) Quantification of experiments shown in (**C**). Colocalisation is shown between *bcd* and Me31B in both wild-type and mutant stage 13 oocytes and in wild-type stage 14 oocytes. Significantly less, or no colocalisation is detected in mutant stage 14 oocytes or both wild-type and mutant activated eggs. *N* = between 6 and 10 oocytes for each genotype, *p*<*0.0001*. (**E**) Representative fixed 10 µm Z-stack image showing the overall distribution of *bcd* (smFISH) in an Endos mutant background at the anterior of the mature oocyte, in vitro activated egg, and early embryo. *bcd* appears to be particulate and diffuse at the anterior pole of all samples. Scale bars: 20 μm. (**F**) Quantification of experiments shown in (**E**). Quantification of the size of *bcd* particles in Endos mutant background compared to the wild type in mature oocytes, activated eggs, and early embryos. Particles in wild-type mature oocytes are significantly larger than in Endos mutant backgrounds. Particles in an Endos mutant background all of a similar size and are not significantly different from particles in wild type activated eggs or early embryos. *N* = 5, Wilcoxon signed-rank test, error bar = SD, *p<0.001* (t = 6.831, df = 5.54). (**G**) Quantification of experiments shown in (**E**). Quantification of the distance from the anterior of *bcd* particles in an Endos mutant background compared to the wild type in mature oocytes, activated eggs, and early embryos. In mature oocytes, particles in an Endos mutant background are significantly further from the anterior than in wild type. Particle distance from the anterior is not significantly different Endos mutant mature oocytes as compared to wild type early embryos. *N* = 5, Wilcoxon signed-rank test, error bar = SD, *p<0.001* (t = 8.81, df = 4.73). Anterior to the left and posterior to the right in all images. ***p < 0.001.

After treating mature eggs with these solutions, we observed BcdSun32 protein in the oocyte anterior (Fig. 2A,B). One caveat to this experiment could be that treating mature eggs with these chemicals results in egg activation, which would in turn generate Bcd protein. To eliminate this possibility, we first screened for phenotypic egg activation markers, including swelling and a change in the chorion (Mahowald et al, 1983). We also applied the classic approach of bleaching eggs for 2 minutes, which causes the lysis of unactivated eggs (Horner and Wolfner, 2008). All chemically treated eggs failed this bleaching test meaning they were not activated (Horner and Wolfner, 2008). While we unable to rule out non-specific actions of these treatments, these experiments corroborate that storage in P bodies which have adopted an arrested physical state is crucial to maintain *bcd* translational repression (Sankaranarayanan et al, 2021).

**Figure EV2:**
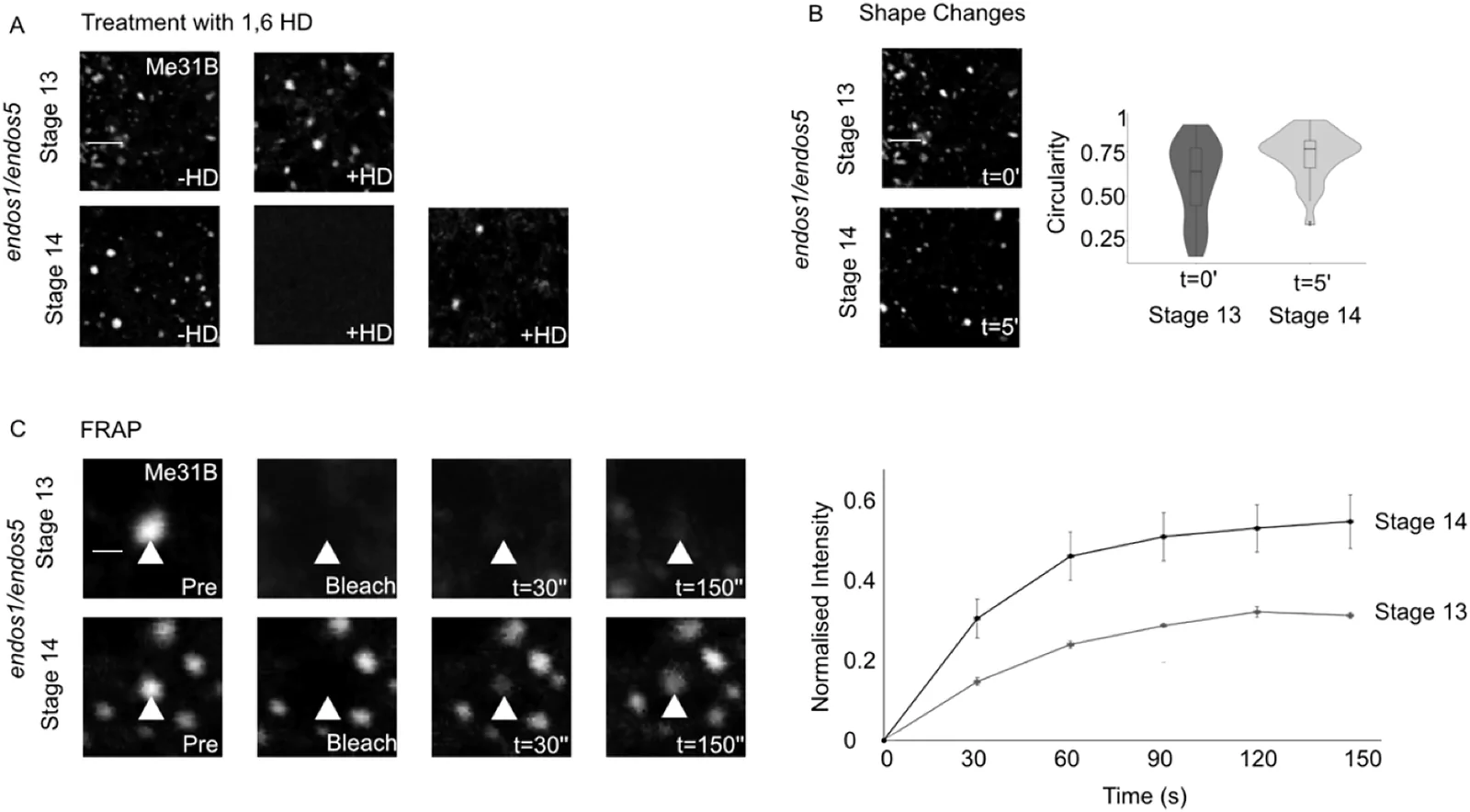
(Related to Fig. 2): P bodies in Endos mutant are less viscous after oocyte maturation. (**A**) Treatment of Endos mutant oocytes with 5% 1,6 hexanediol fails to disperse stage 13 oocyte P bodies within 20 min. The same treatment in stage 14 oocytes leads to almost complete P body dispersal consistent with a more liquid-like nature. There are no particles in 70% of the embryos (left 5% 1,6 hexanediol stage 14 panel), and 30% show reduced particles (right 5% 1,6 hexanediol stage 14 panel). *N* = 10 ooyctes for each stage. (**B**) Live imaging of Endos mutant oocytes during maturation followed by analysis of P body shape as measured by circularity. Wilcoxon signed-rank test, ** p<0.01, *N* = 3 ooyctes for each stage. Figure EV2A and EV2B, stage 13 panels are the same. (**C**) Fluorescence Recovery After Photobleaching (FRAP) of P bodies in Endos backgrounds, shows minimal recovery in stage 13 oocytes. After oocyte maturation there is a higher recovery of fluorescence intensity in stage 14 oocytes, suggesting the condensates are less viscous as they exhibit more exchange with the cytoplasm. *N* = 5 ooyctes for each stage. Scale bars: (**A**, **B**) = 5 µm; (**C**) = 2 µm.

Finally, we used a genetic manipulation to change the physical state of P bodies in mature oocytes. Mutations in *Drosophila* Endosulfine (Endos), which is part of the conserved phosphoprotein α-endosulfine (ENSA) family (Von Stetina et al, 2008), caused a liquid-like P body state after oocyte maturation, similar to that observed with chemical treatment (Fig. 2C) (Sankaranarayanan et al, 2021). This temporal effect matched the known roles of Endos as the master regulator of oocyte maturation (Rangone et al, 2024; Von Stetina et al, 2008). *endos* mutant oocytes lost the colocalisation of *bcd* mRNA and P bodies, concurrent with P bodies becoming less viscous during oocyte maturation (Figs. 2D and EV2). Particle size and position analysis showed that *bcd* mRNA prematurely exhibits an embryo distribution in these mutants (Fig. 2E). Due to genetic and antibody constraints, we are unable to test for translation of *bcd* in the *endos* mutant. However, it follows that *bcd* observed in this diffuse distribution outside of P bodies would be translationally active (Fig. 2E–G).

### *bcd* mRNA translation is spatially restricted during early *Drosophila* embryogenesis

**Figure EV4:**
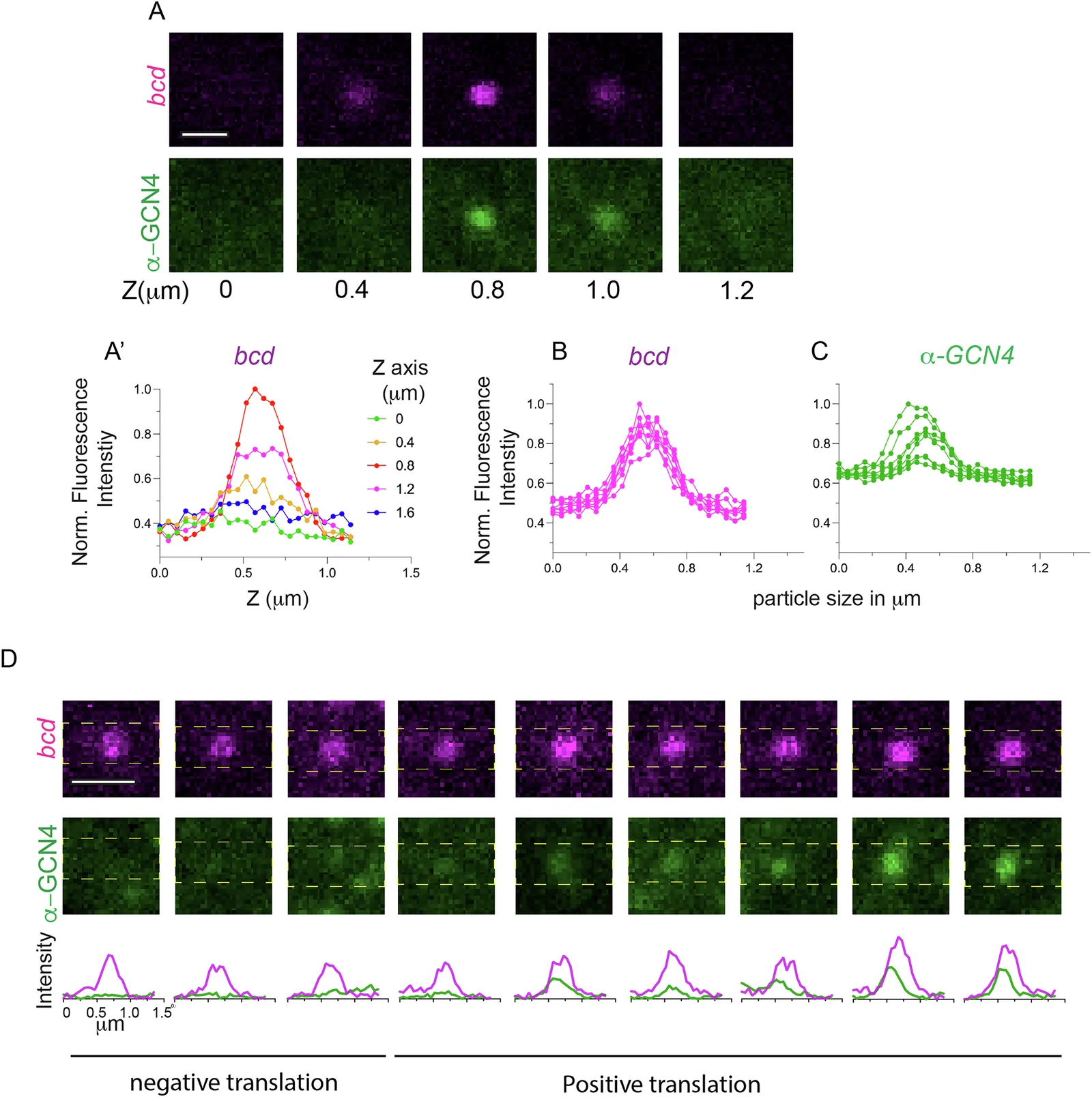
(Related to Fig. 3): Quantification of translating smFISH *bcd*. (**A**) Z-slices of translating *bcd* (*bcd* (magenta), anti-GCN4 (Green)) from a *bcdSun10;bcd*^*E1*^ embryo at n.c.3. The *bcd* intensity from z-slices in (**A**) is captured in (**A**’). (**B**–**D**) Plot showing maximum intensity of multiple *bcd* particles superimposed to show the overall spot size (Magenta, **B**) and variations in the intensity profile of SunTag ((**C**), green). We find mean width of *bcd* spots is 0.7–0.8 µm. The intensity variations of GCN4 are shown in the bottom panel (**D**). Translating and non-translating *bcd* are classified based on the thresholded SunTag signal. Scale bar: (**A**, **D**) = 1 µm.

**Figure 3:**
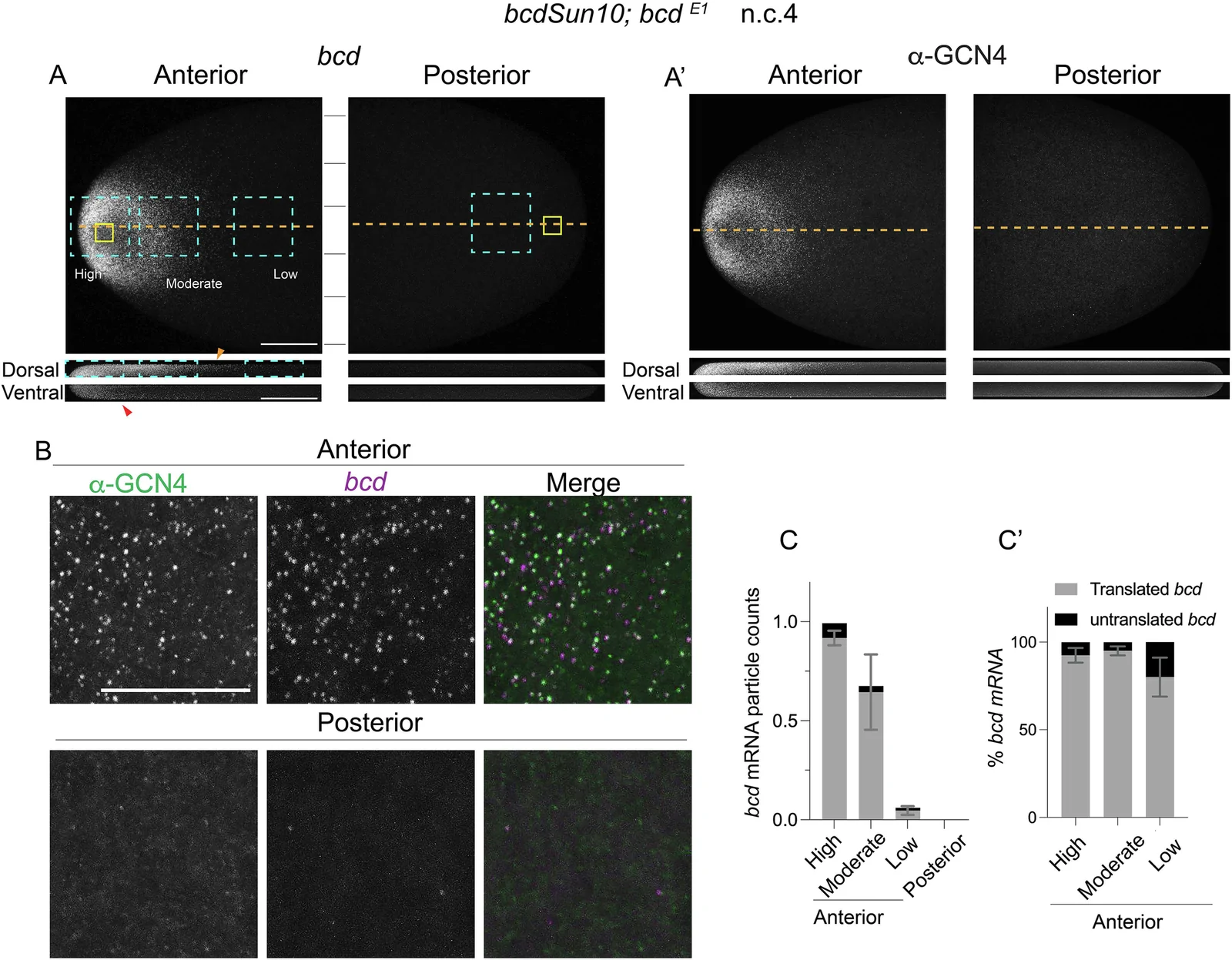
*bcd* mRNA is translated exclusively in the anterior domain of the early *Drosophila* embryo. (**A**–**Aʹ**) Projected Z-stacks of the anterior and posterior domain of n.c. 4 embryo expressing the *bcdSun10* transgene, stained for *bcd* coding region (magenta) and SunTag, anti-GCN4 (green) using Immuno-FISH.ß The projected cross sections in the bottom panel show the dorsal and ventral surface of the anterior and posterior domains of the embryo. Orange and red arrowheads indicate the extent of *bcd* spread on the dorsal and ventral surfaces. Translating *bcdSun10* is shown in (**A**’) along with projected cross sections. Scale bars: 50 µm. (**B**) Yellow boxes from A are magnified to show colocalisation of SunTag to *bcd*. Scale Bars: 10 µm. (**C**) Normalised *bcd* counts from the cyan boxes in (**A**) located at regions of varied mRNA counts across AP-axis. Data normalised to the total mRNA counts from the most anterior region analysed. *N* = 3 embryos. (**Cʹ**) Percentage of translated and untranslated *bcd* mRNA at different locations (cyan boxes in (**A**)) in the anterior and posterior domains (*N* = 3 embryos) in n.c. 4. Error bars = SD.

**Figure EV5:**
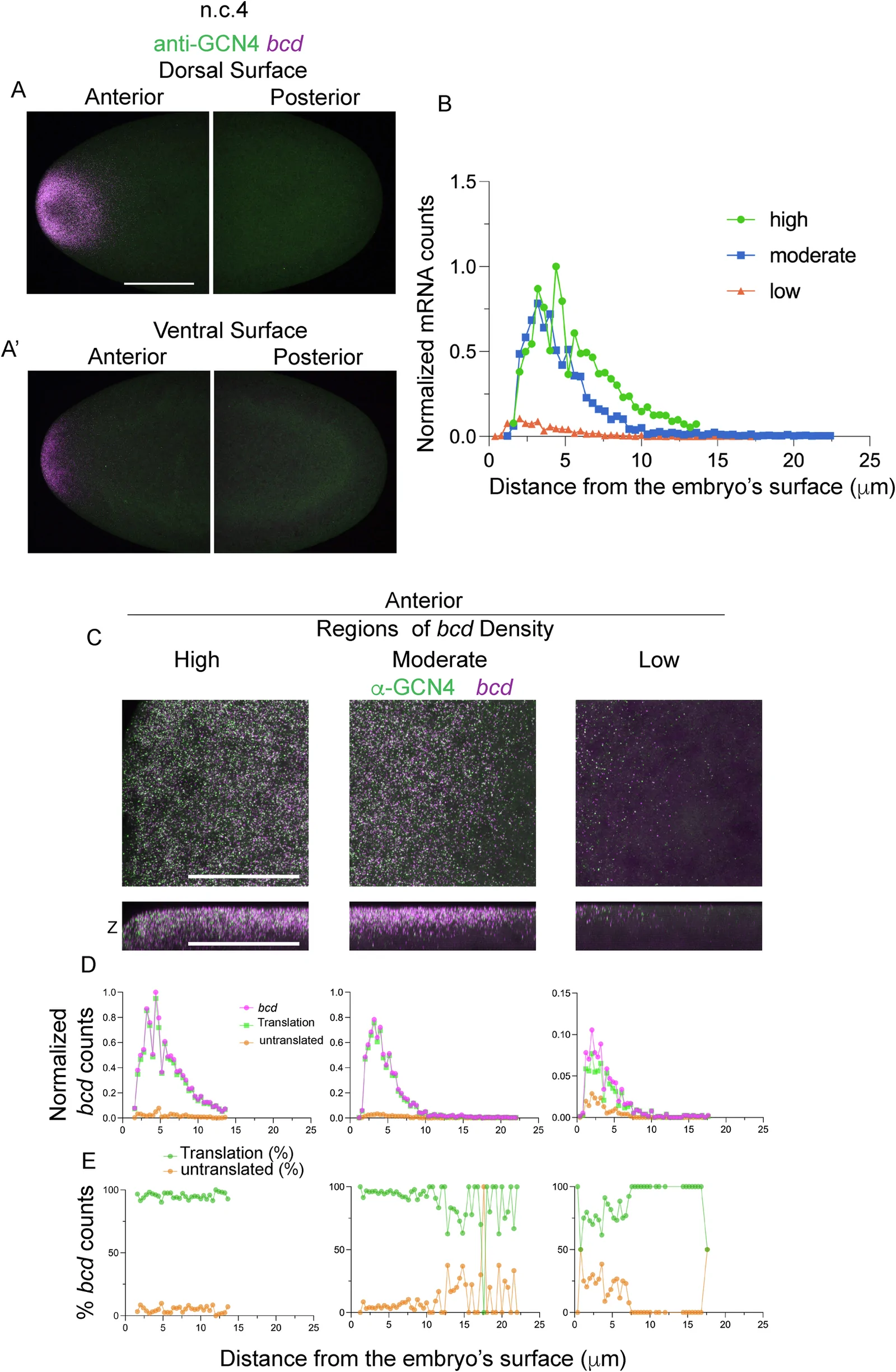
(Related to Fig. 3): Quantification of translation in the anterior domain of the embryo. (**A**,**A’**) Immuno-FISH embryos at n.c.4 for *bcd* (magenta) and anti-GCN4 (Green) on the dorsal surface (**A**) and ventral surface (**A**’). The dorsal surface was reproduced here from Fig. 3A,A’, to compare it with the ventral surface. (**B**) Plot showing the distribution of *bcdSun10* counts from the surface-to-depth of the embryo at n.c.4 and at the anterior domains where the mRNA are of high, moderate and low density. Counts are normalised to the total number of mRNA counts in the most anterior domain. (**C**) Maximum intensity projections of Z-slices and Z-cross sections at high, moderate and low dense *bcd* regions as quantified in (**B**). (**D**, **E**) Normalised *bcdSun10* counts (**D**) and their percentages (**E**) undergoing translation at different positions from the embryo’s cortical surface (2 embryos analysed). Counts are normalised to the total number of mRNA counts in the most anterior domain. Scale bar: (**A**) = 100 µm, (**C**) = 50 µm.

We next addressed when and where the embryo translates *bcd* mRNA, if *bcd* translation is limited to the anterior pole, and when does the maximum translation occur. We considered that the size of *bcdSun*32 may impede mRNA biogenesis and the function of the protein as a transcription factor. Therefore, we generated a second transgene with ten GCN4 repeats (*bcdSun10*) (Fig. EV4). Importantly, *bcdSun10* rescued the *bcd*^E1^ null mutant suggesting that the tag does not greatly affect function. Immuno-smFISH for GCN4 and *bcd* revealed translating and non-translating *bcd* particles in the anterior pole of the early embryo (Fig. 3). Individual *bcd* particles (inferred from diffraction limited spots) colocalised with varying intensities of translating Bcd-SunTag nascent proteins (Fig. EV5). At n.c.4, we saw a strong link between *bcd* mRNA and translation sites in the anterior, with most translation events happening within 100 μm of the anterior pole on the dorsal surface (Fig. 3A,A’). The Bcd protein gradient is known to have small but reproducible differences between dorsal and ventral surfaces (Gregor et al, 2007a). Consistent with this, the translation profile differed between the dorsal to ventral sides, likely due to the asymmetric *bcd* spread on the DV-axis and on their surfaces (Fig. 3A,A’ lower panels, Fig. EV5A,A’).

We next quantified the number of translation events in regions of differential *bcd* density in the anterior domain and at different depths in n.c. 4 embryos (Methods, Fig. EV5B). In the anterior region of the dorsal surface, less than 50 µm from the pole, above 90% of *bcd* particles are engaged in translation (Fig. 3C–C’). Over 98% of all observed translation events occurred within 100 µm of the anterior pole on the dorsal side, with very few translation events seen beyond 100 µm, with negligible *bcd* observed (Appendix Table S6). The translation efficacy does not depend on depth from the cortex, though the number of *bcd* particles reduces significantly from the cortex towards the yolk (Fig. EV5D,E; Appendix Table S7). Overall, we see consistent translation efficacy irrespective of spatial differences. We conclude that during early embryogenesis, before nuclear migration to the cortex, *bcd* translation occurs in the anterior region and it mirrors the anterior-localised *bcd* distribution.

### Spatiotemporal pattern of *bcd* mRNA translation

**Figure 4:**
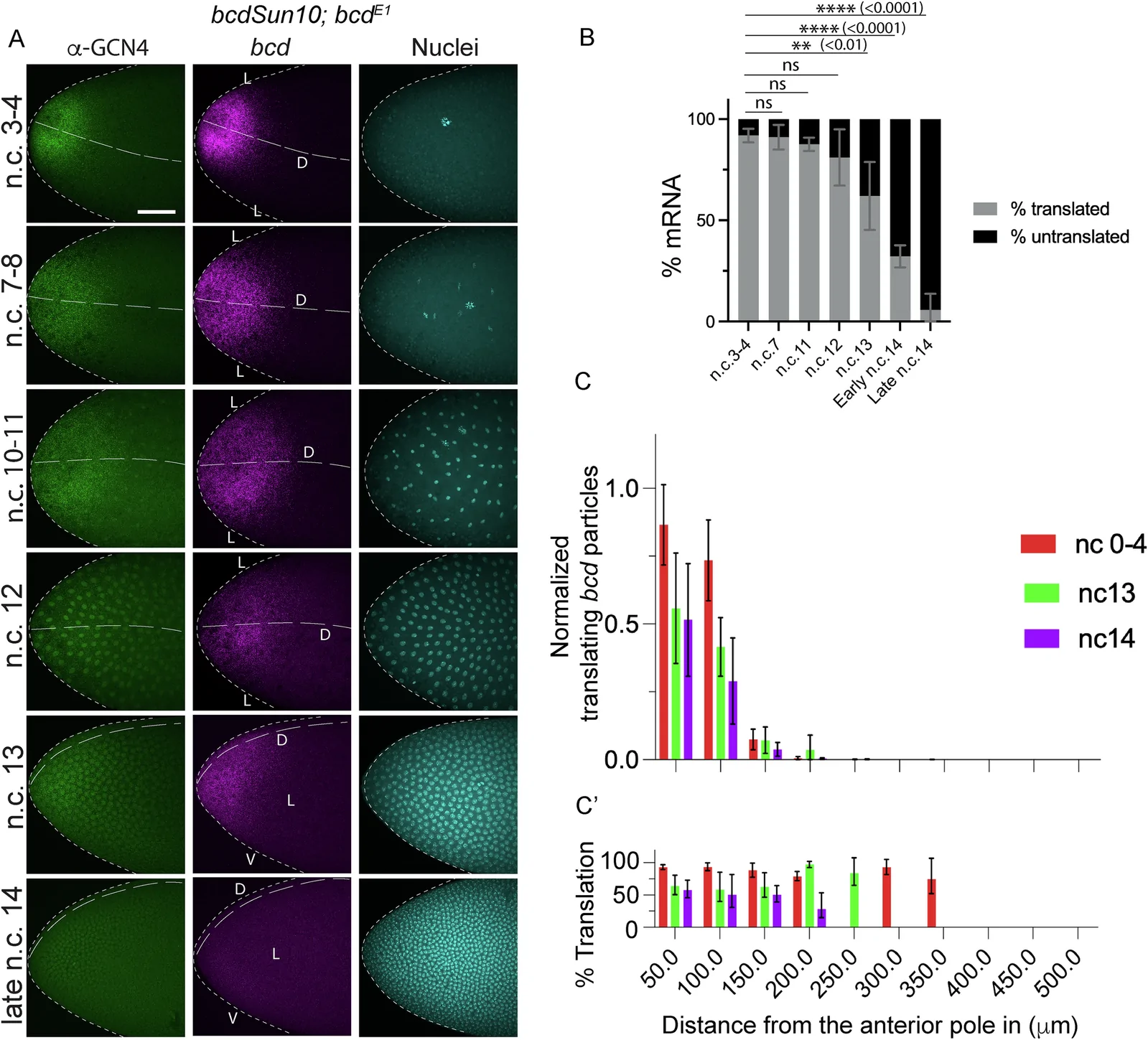
Temporal changes in *bcd* translation from early to late blastoderm. (**A**) *bcdSun10* translation detected through immuno-FISH marked for SunTag translation (anti-GCN4, green) and *bcd* (magenta) from the early to late blastoderm embryo. D = dorsal; V = ventral; V = ventral. Scale bars: 50 µm. (**B**) Mean percentage of *bcd* particles undergoing translation at different nuclear cycles. Error bars = SD. The measure of significance (*P* values) is calculated by two-way ANOVA comparing n.c.3-4 values with rest of the nuclear cycles. *p* < 0.05 is considered significant. *p* = 0.99 (n.c.3-4 vs n.c.7); *p* = 0.98 (n.c.3-4 vs n.c.11); *p* = 0.576 (n.c.3-4 vs n.c.12); *p* = 0.0045 (n.c.3-4 vs n.c.13); *p*<*0.0001* (n.c.3-4 vs Early n.c.14); *p*<*0.0001* (n.c.3-4 vs Late n.c.14). (**C**) Mean measure of translating *bcd* particles in 50 μm regions from the anterior pole along the mid-dorsal surface of the anterior-posterior axis. (**C’**) Mean percentage of *bcd* mRNA particles observed undergoing translation. Note, for >100 μm, very few *bcd* mRNA particles were observed, as shown in (**C**). For (**B**, **C**), *N* = 3 embryos for each nuclear stages, with error bars = SD.

**Figure EV6:**
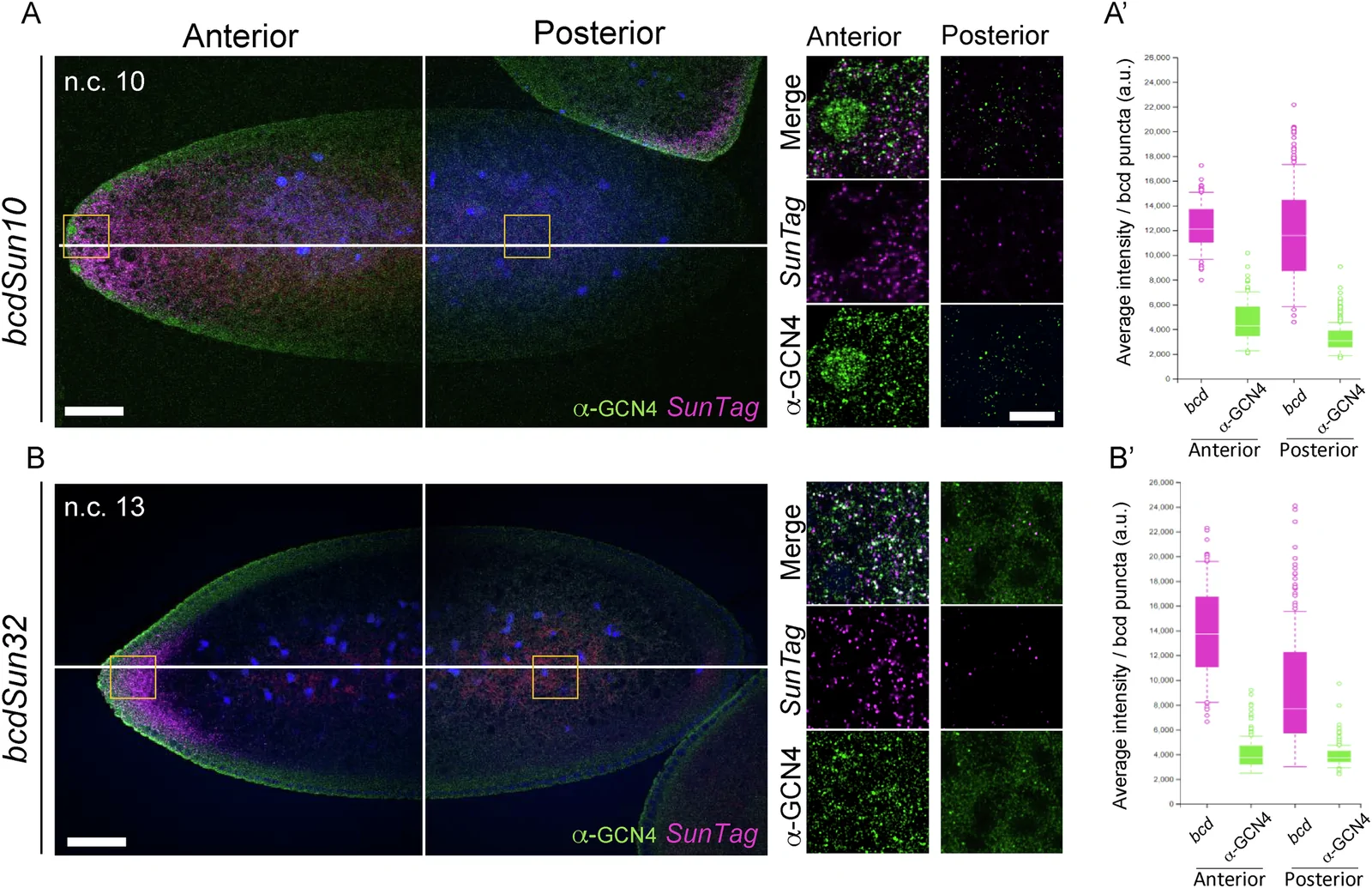
(Related to Fig. 4): quantification of translation intensity of *bcd* in the anterior and posterior domains. (**A**–**B’**) Projected Z stacks of the anterior and posterior domains embryos expressing *bcdSun*32 (**A**) and *bcdSun*10 (**B**) transgenes. *Bcd* reporters and translation spots were detected by Immuno-FISH using Suntag probes and anti-GCN4 shown in magenta and green, respectively. Quantification of the single molecule average intensity for bcd-reporters FISH and GCN4 IF signals in anterior and posterior domains (**A**’, **B**’) (right panel).

How does translation of *bcd* vary during embryogenesis? We analysed 3D image stacks in the anterior domain (within 50 μm from the pole) at different stages of blastoderm development from n.c. 4 through n.c. 14. Before n.c.9, there was sustained translation of *bcd* in a region near the anterior cortex (Fig. 4A). After nuclear migration in n.c. 9, we observed a gradual decrease in translation events and by the end of n.c. 14, translation events were scarcely detected (Fig. 4A,B; Appendix Table S8). We further employed this analysis across the entire embryo length at pre- and post-nuclear migration stages of the blastoderm to probe for changes in *bcd* translation along the anterior-posterior axis (Fig. 4C). We found a negligible number of *bcd* particles scattered beyond 100 μm on the dorsal surface and we detected translation in few of these particles (Fig. 4C’, Table S9). Therefore, we conclude that there is no significant gradient of translation of *bcd* through the embryo. To test if the efficiency of translation varied along the anterior-posterior axis, we quantified and compared the intensity of translation events. We did not see any clear changes in the intensity of translation events between these locations (Fig. EV6).

**Figure EV7:**
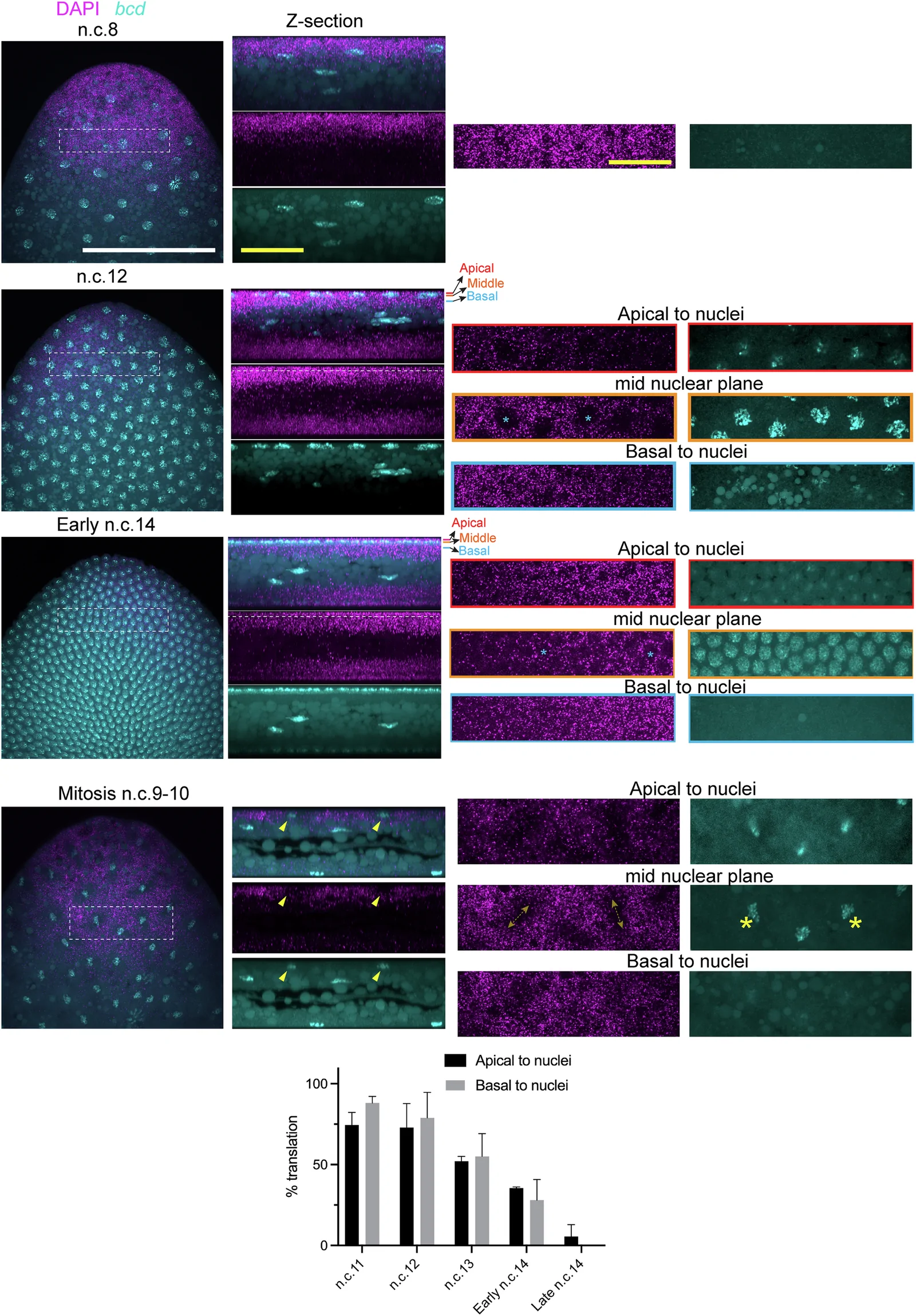
(Related to Figs. 4 and 5): Redistribution of *bcd* mRNA upon cortical migration and division of blastoderm nuclei. Left panel: Anterior domain of flattened embryos staged at pre-cortical nuclear migration stages (n.c.8), post cortical nuclear migration stages at n.c.12 and early n.c.14, and at n.c.9-10 mitosis stages stained for *bcd*(magenta) and Dapi (cyan). Middle panel: Z-section of a magnified region (broken box) of the aforementioned staged embryos showing *bcd* distribution across the depth. Broken white lines in the *bcd* single channel of n.c.12 and n.c.14 segregates nuclear boundary. Yellow arrowheads indicate mitotic nuclei. Right panel: X–Y planes of the magnified region in the left panel. Apical, mid, and basal nuclear planes are shown for n.c.12, 14 and mitosis stages. Cyan stars indicate nuclear regions. Yellow double arrowheads indicate the direction of the nuclear division. Yellow stars indicate mitotic nuclei. Bottom: Barplot showing variation in *bcd* translation localised apical and basal to the cortical nuclei. *N* = 3 embryos per nuclear cycle. Scale bars: left panel = 100 µm; middle panel (yellow) = 50 µm; right panel (yellow) = 50 µm.

Translation can depend on the location of mRNA within the cytoplasm. In the early *Drosophila* embryo, for example, apical *twist* mRNA has lower translation than basal mRNAs (Dufourt et al, 2021). We analysed and compared the translating *bcd* particles above and below the cortical nuclei from n.c.9 to late n.c.14. We did not see any differences in the translation of *bcd* based on their position with respect to the nuclei position (Fig. EV7). We also noticed that upon migration of nuclei to the cortex, *bcd* localisation at the anterior pole became more dispersed (Fig. EV7). The interphase nuclei and their subsequent mitotic divisions appeared to displace *bcd* towards the apical surface (Fig. EV7).

Our *bcdSun10* not only marks the translating nascent Bcd-SunTag but can also generate a functional gradient. We can directly test different models of Bcd protein production (Driever and Nüsslein-Volhard, 1988; Durrieu et al, 2018; Little et al, 2011; Spirov et al, 2009) and how this impacts the formation of the Bcd morphogen gradient. We first crossed *bcdSun10* line with *nos-ScFv-mNeonGreen* to image Bcd gradient formation in live embryos. Our imaging revealed the emergence of *BcdSun10* gradient, however, the dynamics of *bcdSun10* nuclear shuttling is rapid, rendering quantification difficult (Supplementary Video 1). This could be due to insufficiency in the level of nos-driven ScFv-mNeonGreen.

**Figure EV8:**
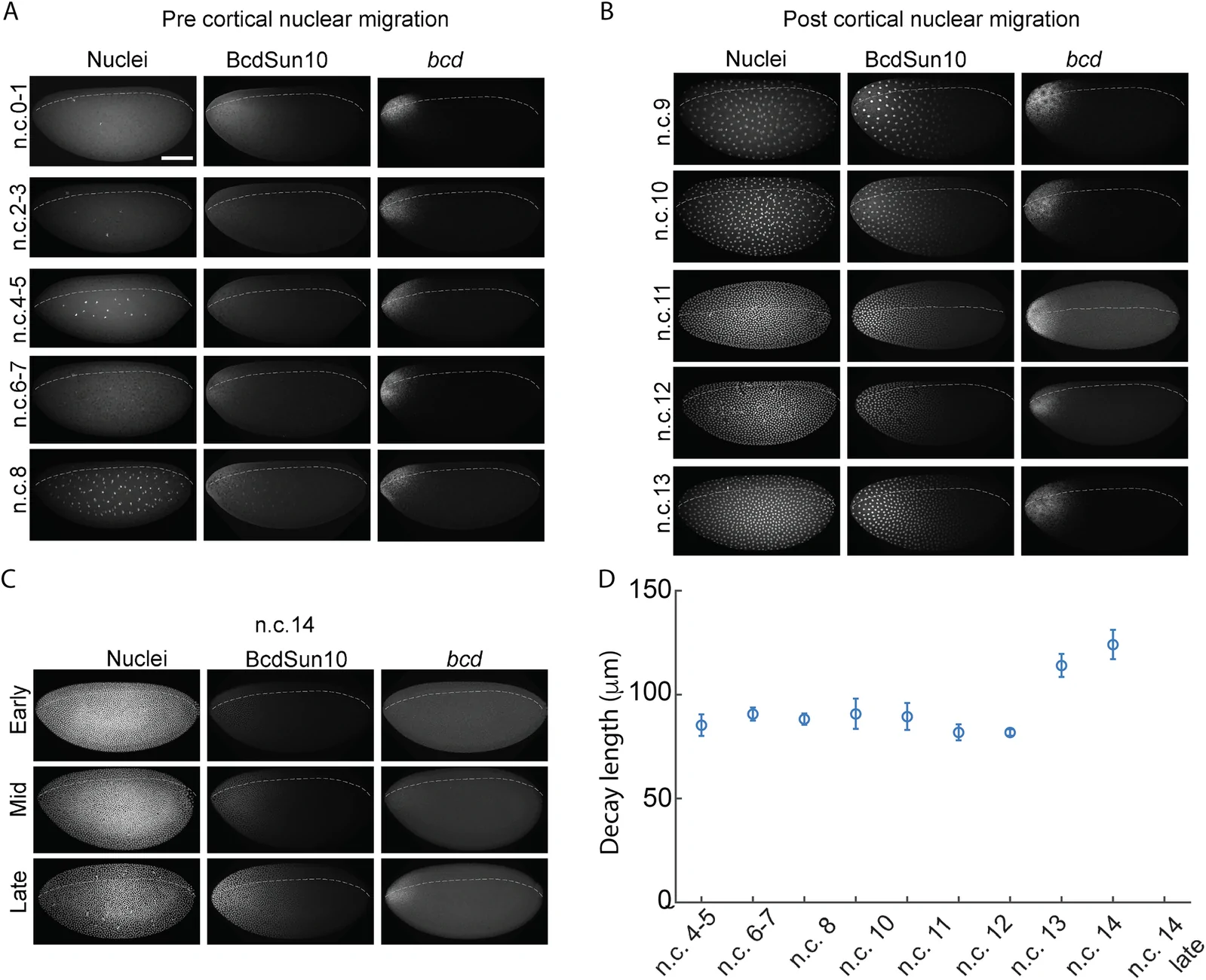
(Related to Fig. 5): The *bcdSun10* gradient is formed by spread from the translation domain. (**A**–**C**) *bcdSun10;bcd*^*E1*^ embryos at different stages, showing the evolution of the SunTag-Bcd gradient (middle panel) in pre-cortical (**A**), post-cortical nuclear migration (**B**) and n.c.14 (**C**) with respect to the *bcd* distribution (right panel). The embryos are stained with anti-GCN4 antibody (Bcd-SunTag), *bcd* (mRNA) and DAPI (nuclei). Embryos classified based on the nuclear counts and internuclear distances from the DAPI staining. Broken lines indicate the mid-dorsal lines of the embryo. Scale bar: 50 µm. (**D**) Quantification of the decay length of the BcdSun10 gradient between 100 μm to 350 μm (from data presented in Fig. 5). Equation of fit: $\left[{Bcd}\right]={\left[{Bcd}\right]}_{0}{e}^{-x/\lambda }$, where λ is the measured decay length. Error bars 95% confidence intervals. Increase in n.c.14 due to decreasing Bcd concentration, leading to flatter profile.

**Figure 5:**
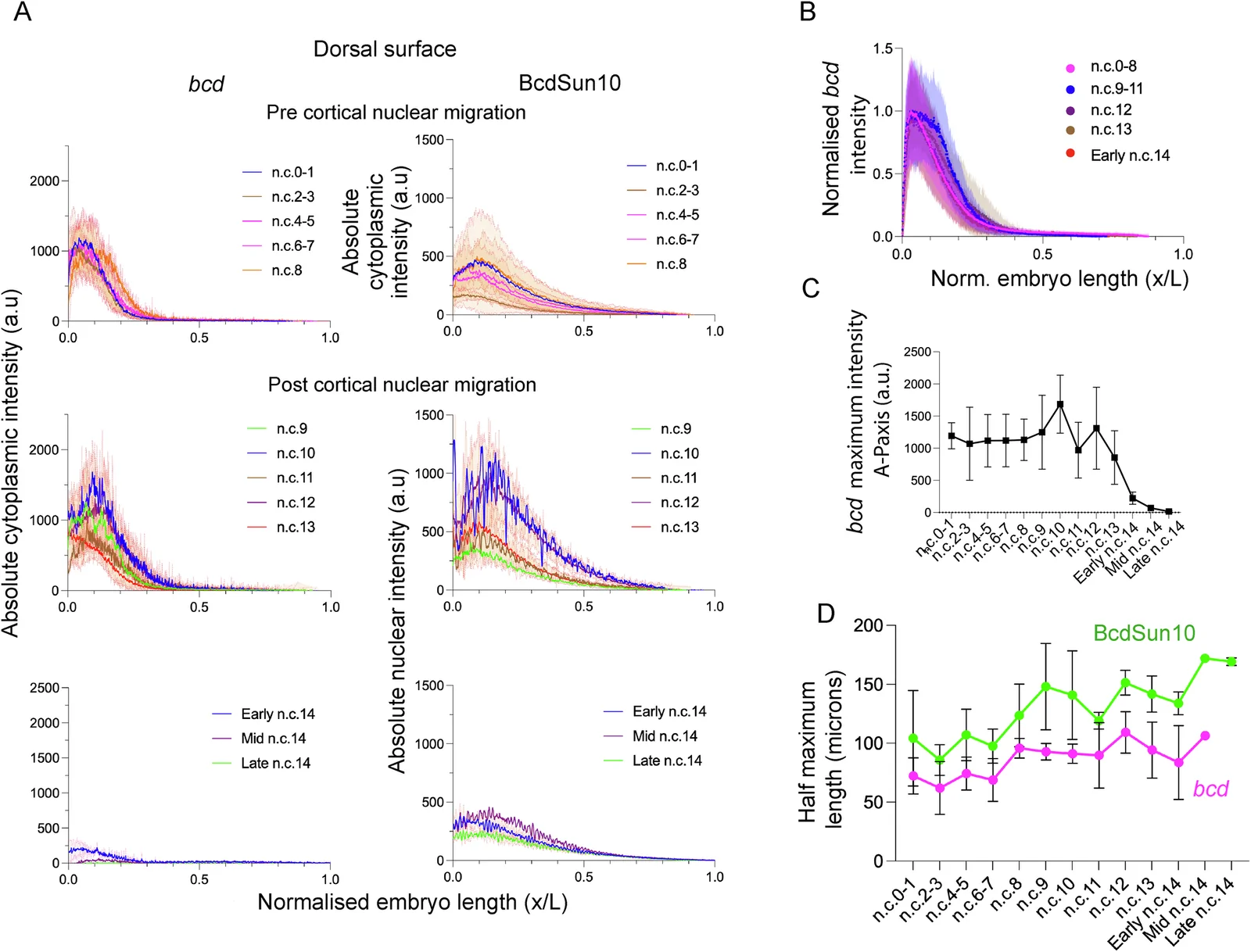
The *bcdSun10* gradient is formed by spread from the translation domain. (**A**) Line plots showing the intensity of *bcd* (left) and anti-GCN4 (right) measured across the AP-axis of the embryo’s mid-dorsal surface at different blastoderm stages. Background shading represents ±1 SD. (**B**) Line plot showing the intensity of *bcd* across the AP-axis of the embryo’s mid dorsal surface. Background shading represents ±1 SD. Intensity normalised to the *bcd* intensity maxima. (**C**) Line plot of the mean *bcd* maximum intensity distribution at different nuclear cycles. Error bar = SD. (**D**) Line plot of the mean distance from the anterior pole for *bcd* maximum intensity to decrease by half from the anterior peak value (Half maximum length) along the AP-axis. Error bar = SD. (**B**–**D**) embryo numbers: *N* = 3 (n.c.0-1), *N* = 4 (n.c.2-3), *N* = 5 (n.c.4-5), *N* = 3 (n.c.6-7), *N* = 3 (n.c.8), *N* = 3 (n.c.9), *N* = 3 (n.c.10), *N* = 2 (n.c.11), *N* = 4 (n.c.12), *N* = 6 (n.c.13), *N* = 2 (Early n.c.14), *N* = 1 (Mid n.c.14), and *N* = 2 (Late n.c.14).

Therefore, we resorted to quantification of translation using fixed embryo samples at different time points. We compared the concentration profiles of *bcd* mRNA and the protein distribution in our *bcdSun10* line on the embryo’s dorsal surface from n.c.1-8 (prior to cortical nuclear migration), n.c.9-13 (post cortical nuclear migration), and during n.c.14 (leading up to cellularisation) (Fig. EV8A–C). We noticed an overall spread of BcdSun10 beyond the *bcd* signal, consistent with an SDD-like model for gradient formation (Fig. 5A), with a good fit to an exponentially-decaying profile (Fig. EV8D). The extent of *bcd* mRNA was localised to the anterior during n.c.1 to 8, with some dispersal post cortical nuclear migration (Fig. 5B). However, there was no apparent *bcd* mRNA gradient extending across a significant part of the embryo; again, the vast majority of translation events were within 100 μm of the anterior pole. We saw a reduction of Bcd intensity in n.c.14, consistent with previous reports (Fig. 5C) (88). Critically, the BcdSun10 protein gradient always had further extent than the *bcd* mRNA distribution (Fig. 5D).

Overall, we observe a small but steady shift in the domain of *bcd* mRNA translation, which correlates with nuclei movements. However, as this change in translation domain is small relative to the size of the embryo, we do not observe substantially more dispersed *bcd* translation along the AP-axis. More than 98% of all translation events occurred within 100 μm of the anterior pole, regardless of the nuclear cycle. These results support a model of localised Bcd production, restricted to a domain near the anterior pole.

### *bcd* mRNA degradation in n.c. 14 embryos

**Figure 6:**
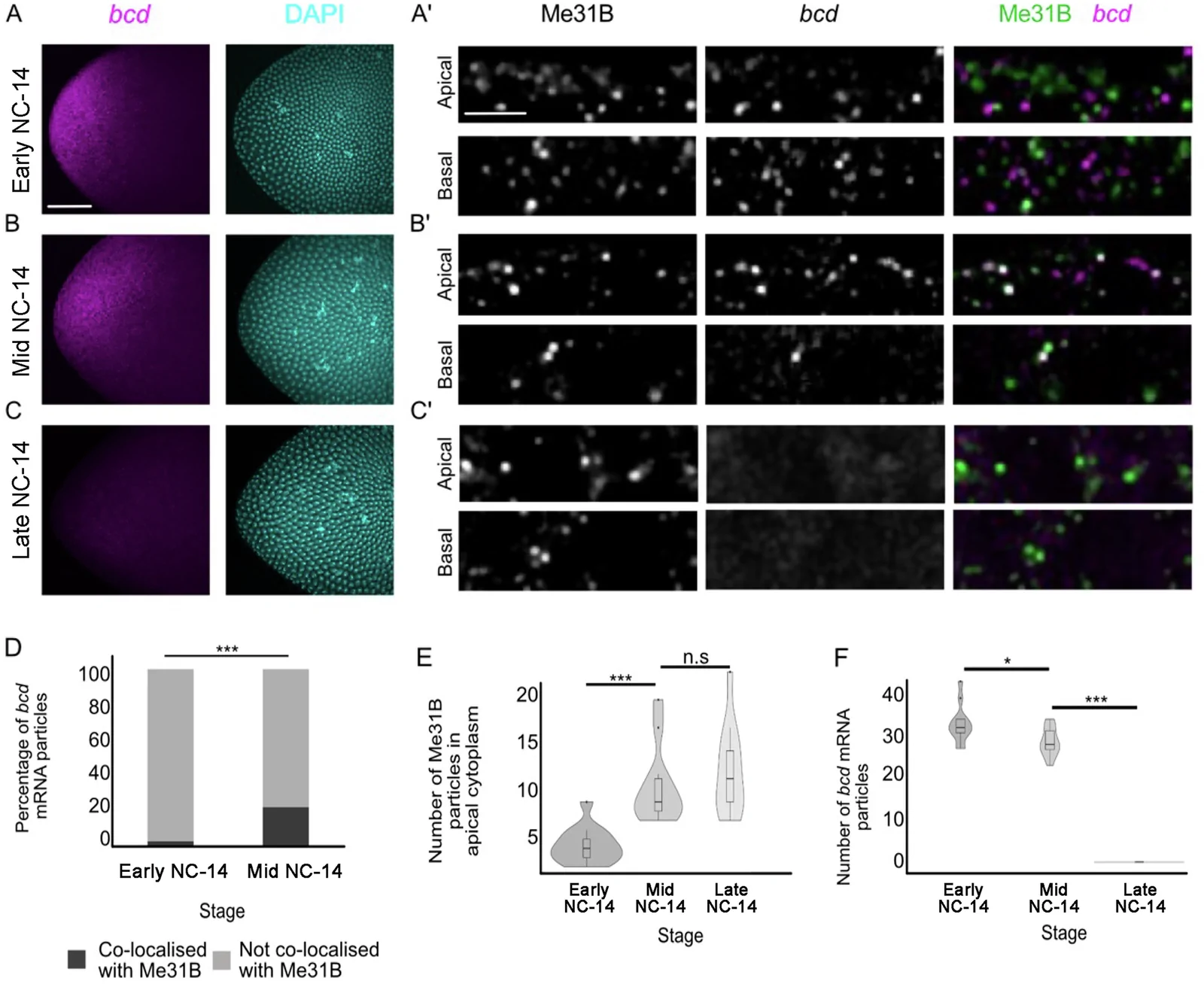
*bcd* re-associates with P bodies in n.c. 14, coinciding with their degradation. (**A**–**C**) Representative fixed projected Z-stack images (from 20 embryos) showing *bcd* (magenta, smFISH) and DAPI (cyan) early n.c. 14, mid n.c. 14, and late n.c. 14. Scale bars: 50 µm. (**Aʹ**–**Cʹ**) Representative fixed single plane images showing the apical side and basal side of early n.c. 14, mid n.c. 14, and late n.c. 14. Me31B::GFP (anti-GFP-nanobody), *bcd* (smFISH), and merge shown. *N* = 10 embryos. Scale bars: 10 µm; zoom 5 µm. (**D**) Colocalisation between *bcd* and Me31B::GFP particles in the apical cytoplasm early and mid n.c. 14. *N* = 5 embryos, Fisher’s exact test, *p* < 0.0001. (**E**) P body number in the apical cytoplasm between early n.c. 14 and mid n.c. 14. *N* = 5 embryos, unpaired t test, *p* < 0.0001 and *p* = 0.4733. (**F**) Number of bcd particles present in the apical cytoplasm through early n.c. 14, mid n.c. 14, and late n.c. 14. *N* = 5 embryos, unpaired t test, 0.0291 and <0.0001. (**E–F**) In the box-and-whisker plots, the box extends from the 25th to the 75th percentile, the whiskers show the minimum and maximum values, and the line within the box represents the median. The overlaid violin plot indicates the distribution of the data, with minima and maxima of the shaded area representing the data range. (**D**–**F**): *p < 0.05 ; ***p < 0.001.

We showed a significant reduction in *bcd* mRNA translation from n.c. 13 onwards, and smFISH confirms that during n.c. 14 *bcd* is degraded (Figs. 5C and 6A–C) (Spirov et al, 2009; Surdej and Jacobs-Lorena, 1998). Previous work has shown mRNA colocalisation with embryonic P bodies is correlated with degradation (Eichler et al, 2020; Forbes Beadle et al, 2023; Wang et al, 2017a). We next tested if *bcd* was targeted for degradation in embryonic P bodies. We observed *bcd* and Me31B at early, mid, and late n.c. 14 (Fig. 6Aʹ–Cʹ). With smFISH, we showed that *bcd* was evenly distribution on both the apical and basal sides of the nuclei. Colocalisation analysis revealed that *bcd* is not in P bodies at early n.c. 14 (Fig. 6Aʹ,D).

At mid n.c. 14, *bcd* was localised to the apical side of the nuclei (Spirov et al, 2009). We found that it colocalised with embryonic P bodies, which were also enriched on the apical side (Fig. 6Bʹ,E,F). As the embryo transitioned into gastrulation, we observed a significant loss of *bcd* while embryonic P bodies remained (Fig. 6Cʹ–E,F). Together, this supports a model where, as the embryo transitions to zygotic control, maternal mRNAs in the embryo gather in embryonic condensates for degradation (Eichler et al, 2020; Forbes Beadle et al, 2023; Wang et al, 2017a).

### Adapted SDD model of Bcd protein gradient formation

Formation of the Bcd morphogen gradient has been extensively modelled (Grimm et al, 2010; He et al, 2010; Huang and Saunders, 2020). Previous models typically considered the rate of Bcd production stayed constant during early embryogenesis. We asked if considering the spatiotemporal pattern of *bcd* translation in the SDD model matched the observed Bcd gradient.

**Figure 7:**
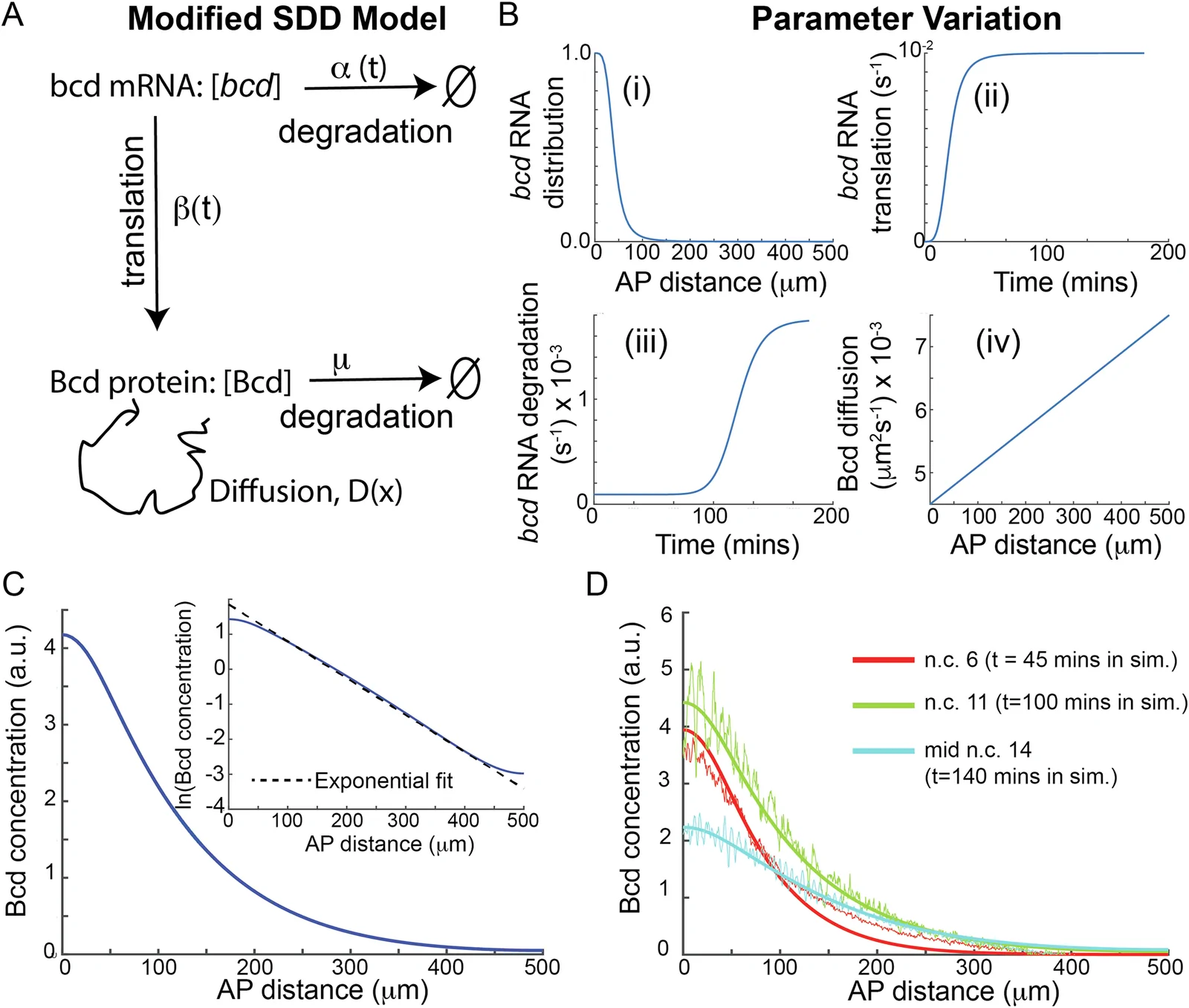
Modified SDD model of Bcd gradient formation. (**A**) Model schematic, outlining parameters defining bcd and Bcd production, degradation and movement. (**B**) Initial conditions (i) and parameter variation (ii–iv) over time or space within the model. (**C**) Bcd concentration after 120 min in the simulation. (**Inset**) Same as (**C**) on log-plot, with an exponential fit shown with the dashed black line. Dashed line has decay length of 85 µm. (**D**) Comparison of model prediction (solid lines) with experimental measured gradients at different time points (data points). Error bars not shown for clarity (see Fig. EV9B).

We considered the concentrations of *bcd* mRNA, shown as *[bcd]*, and the protein *[Bcd]*, where the concentrations are functions of space and time, within a modified SDD model (Fig. 7A):

1a $$\frac{\partial \left[{bcd}\right]}{\partial t}=-\alpha (t)[{bcd}]$$

1b $$\frac{\partial \left[{Bcd}\right]}{\partial t}=\frac{\partial }{\partial x}\left[D\left(x\right)\cdot \frac{\partial \left[{Bcd}\right]}{\partial x}\right]-\mu \left[{Bcd}\right]+\beta [{bcd}]$$

We defined time t = 0 to denote egg activation. The initial conditions for [*bcd]* and [Bcd] were given by:

2a $$\left[{bcd}\right]\left(t=0,x\right)=f(x)$$

2b $$\left[{Bcd}\right]\left(t=0,x\right)=0$$

*bcd* mRNA was localised in the anterior pole with a form denoted by *f(x)*, (Eq. 2a), shown in Fig. 7B(i). Motivated by our results with regards to onset of Bcd translation post egg activation, we incorporated a delay initially in translation to account for egg activation (Fig. 7B(ii)). The rate of *bcd* degradation *α* (*t*) increased markedly in n.c. 14 (Fig. 7B(iii)), motivated by results in Fig. 6.

It was recently observed that the effective diffusivity of Bcd protein varies between the anterior and posterior pole (Athilingam et al, 2024). included this through spatial variation in the effective diffusion coefficient *D(x)* (Fig. 7B(iv)). We assumed that the rate of *bcd* translation after egg activation (β) and Bcd degradation (μ) remained constant. The degradation rate for Bcd has been calculated previously based on protein lifetime measurements (Durrieu et al, 2018).

**Figure EV9:**
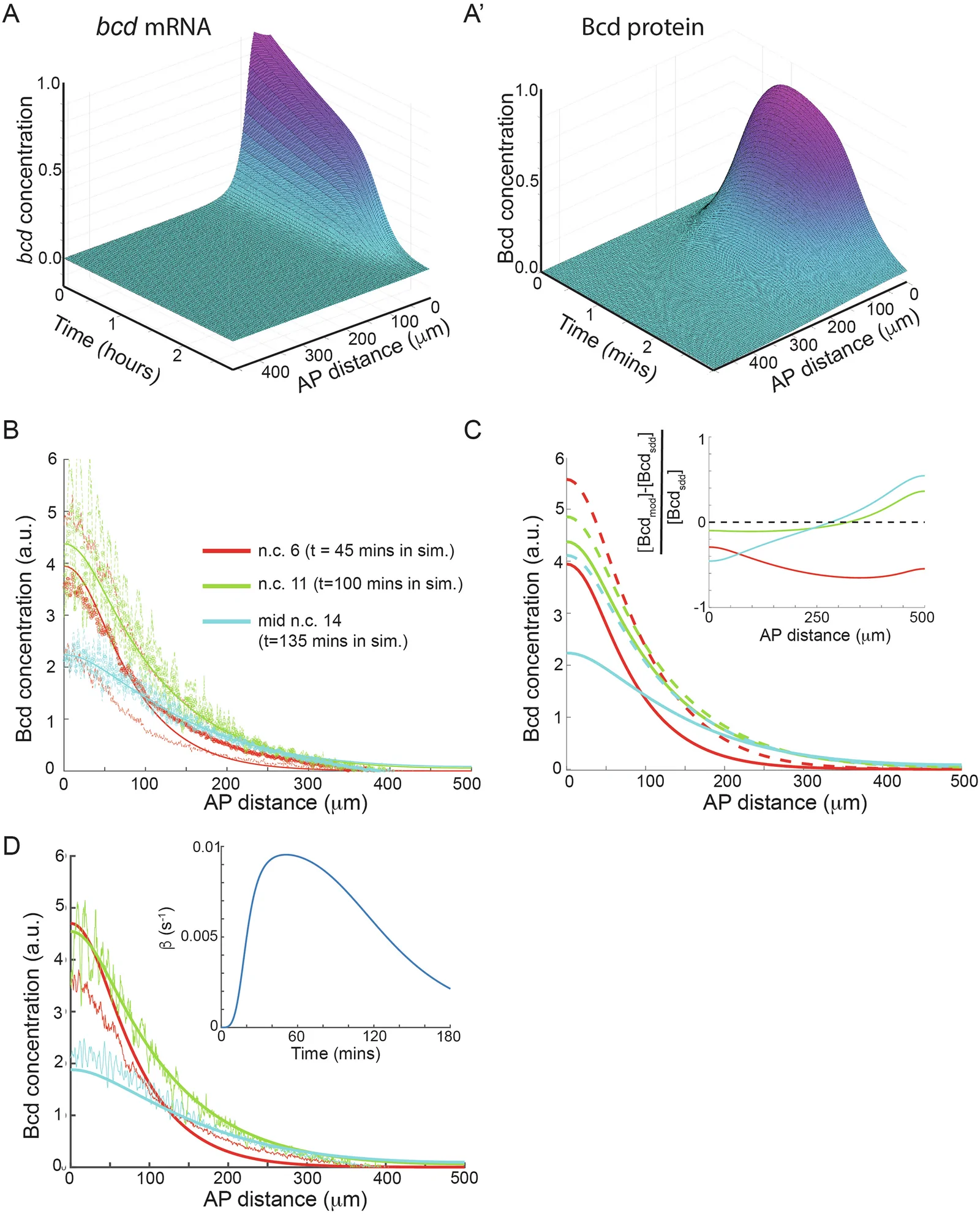
(Related to Fig. 7): Modified SDD model of Bcd gradient formation. (**A**) Normalised concentration (by maximum value) in space and time for *bcd* (**A**) and Bcd protein (**A**ʹ) from the modified SDD model. (**B**) Same as Fig. 7D but showing the standard error on the experimental concentration measurements (dashed lines). (**C**) Comparison of SDD (dashed) and modified-SDD (solid) models, with colour scheme same as (**B**). Inset shows the relative difference in the two models. Here, the SDD model is taken to have constant diffusion coefficient, mRNA degradation, and translation rates (Table S5). (**D**) Modified SDD model with time varying β parameter, as shown in inset. Colour coding same as in (**B**).

Our modified one-dimensional SDD model generated an exponential-like gradient of Bcd (Figs. 7C and EV9A), which matched the observed in vivo Bcd profile at different time points, especially after n.c. 9 (Figs. 7D and EV9B). We emphasise that all the parameters in this model are constrained except β, so it is not over parameterised (Appendix Table S5). We found a decay length of around 85–95 µm, consistent with our and previous experimental measurements (Durrieu et al, 2018; Gregor et al, 2007b; Little et al, 2011) (Fig. EV9D). The overall behaviour of the Bcd protein gradient (Fig. 7D) is consistent with previous observations (Little et al, 2011) and our quantification presented here, and represents an improved fit on the naive SDD model (Fig. EV9C).

Critically, this model can explain numerous experimental observations. (i) Bcd reduction in n.c. 13-14. It was previously shown that Bcd production appears to decrease from n.c. 13, but the reason was unclear. Here, our experimental and theoretical results show this is driven primarily by a reduction in Bcd production and does not require a significant increase in the Bcd degradation rate. (ii) The Bcd gradient emerges gradually during early embryogenesis. This is due to the activation of Bcd translation only after egg activation. The Bcd gradient does not form from an initial pulse during oogenesis. (iii) Dynamic measurements of Bcd (FCS ~ 7 μm^2^s^−1^, FRAP ~ 1 μm^2^s^−1^) can be explained by their being different effective pools of Bcd, with both fast and slow populations (Athilingam et al, 2024), with the FRAP measurements mainly assaying the slow population. (iv) The Bcd gradient can form across the embryo on relevant time scales without a dispersed gradient of *bcd* mRNA. Further, we see no evidence for significant change in the spatial extent of *bcd* mRNA. Overall, the SDD model—with suitable experimentally justified modifications—can explain the dynamics of Bcd gradient formation without requiring other mechanisms (e.g., *bcd* mRNA gradient or protein production in the oocyte).

## Discussion

We have provided a quantitative dissection of when and where *bcd* mRNA is translated in early *Drosophila* development. Translation is restricted until after egg activation by localisation within P bodies. Translation occurs in the anterior pole of the embryo and undergoes a marked decrease after n.c. 12. As embryogenesis continues, *bcd* becomes localised to the apical domain and is degraded, particularly at mid n.c. 14. This rapid degradation of *bcd* correlates with its localisation to P bodies during embryo cellularisation. These observations, along with recent quantifications of Bcd dynamics (Athilingam et al, 2024), support a modified SDD model of gradient formation.

In early embryos, Bcd protein is only observed in the anterior pole. This closely matches the *bcd* translation pattern. No significant translation was observed more than 50 μm from the embryo anterior. These results argue that *bcd* translation does not occur in the oocyte and against a model of protein gradient formation by a dispersed zone of production. Another model suggests that *bcd* is distributed through the embryo and hence can generate a Bcd gradient without the need for diffusive transport (Baumgartner, 2018; Shlemov et al, 2021; Spirov et al, 2009). However, we observe most translation events occurred within 50 µm of the embryo anterior pole. Consistent with previous reports (Little et al, 2011), we posit this is due to *bcd* localisation in the anterior region, rather than there being translational regulation of *bcd* in more posterior regions. This means that any graded form of *bcd* translation mostly happens in the anterior and hence a gradient of *bcd* translation does not underlie formation of the Bcd protein gradient with a decay length of around 85 μm.

We noticed distinct differences in the apical-basal localisation of *bcd*, especially after nuclear migration to the embryo cortex. This change in apical-basal distribution correlates with a reduction in the observed *bcd* translation. However, it still needs to be tested if the localisation of *bcd* impacts its translation rate. The localisation of mRNAs within the microenvironment defined by each nuclear domain can impact translation in n.c. 14 (Dufourt et al, 2021), though whether a similar effect occurs earlier in development is unclear.

The rapid loss of *bcd* at n.c. 14 has been previously quantified (Little et al, 2011) and here we discovered it also correlates tightly with localisation to P bodies (Fig. 6). It remains unclear what determines the model of *bcd* degradation at n.c. 14, and if it is due to global degradation of maternal mRNA or a specific regulation of *bcd* mRNA (Wang et al, 2017b). Testing for *bcd* degradation is challenging. One method is to infer degradation by examining spectral signal with different smFISH probes for the 5’ and 3’ UTRs (Forbes Beadle et al, 2023; Kramer, 2017). However, the *bcd* 5’ UTR is very short, and we were unable to make effective probes. An alternative approach is to target the *bcd* instability element (BIE). This regulates degradation of *bcd* (Surdej and Jacobs-Lorena, 1998) and is located within the more accessible 3’ UTR.

It will be interesting to explore how differences in P bodies between the oocyte and embryo affects mRNA. P bodies appear to switch function, from shielding *bcd* from translation to enhancing *bcd* degradation. Prior to cellularisation, we found that Me31B condensates were insensitive to cycloheximide. However, after cellularisation begins, they become sensitised to cycloheximide (Patel et al, 2016), suggesting a change in their structure. A similar change in RNP function has been shown in *Drosophila* germ granules (Hakes and Gavis, 2023). Germ granules transition from protecting their mRNA contents to targeted degradation of specific mRNAs after germ cell specification. This is accomplished through recruitment of degradation factors as the granules grow through fusion. Decapping activity in these enlarged, functionally different germ granules lead the authors to call them P body-like. It is intriguing to consider that many RNP granules in *Drosophila*, and more broadly in *C. elegans, Xenopus*, zebrafish, will likely undergo changes in size, composition, and function throughout development. Relatedly, due to the conserved nature and wide-ranging downstream effects, it is intriguing to consider the possibility that Endos is a condensate regulator both within *Drosophila* eggs as well as more generally.

Over 40 years of work span from the original proposition of the SDD model for Bcd protein gradient formation. Here, we quantitatively assay the production of Bcd in vivo, which is arguably the last piece required to understand Bcd gradient formation. We see that the original SDD model, which assumed that dynamic parameters such as production rate and diffusion were constant in space and time, was too simple. However, with suitable modifications, like spatially varying diffusion and temporally altering translation, it can explain the formation of the Bcd gradient. The parameters in our model are all constrained by experimental observations, with the exception of the parameter β, which represents the translation rate. It is plausible that β itself decreases at later n.c.’s; i.e., the translation rate decreases, rather than the pool of *bcd* mRNA reducing as modelled in Fig. 7. Modelling such behaviour of β (Fig. EV9D) can also generate a gradient of Bcd that is similar to experiment. It will be interesting to dissect this, and other questions, for example related to expanding to 3D, in further work utilising live imaging approaches.

## Methods

**Table:** Reagents and tools table

Reagent	Supplier/provider	Catalogue number	Concentration used
**Antibodies**			
GFP Booster Alexa 488	Chromotek	gb2AF488-100	1:1000 in 0.1% PBST
Rabbit GCN4 Antibody (C11L34) - Chimeric	Novus Biologicals	NBP2-81274SS	1:200 in 0.1% PBST
Goat anti-Rabbit Cy3	Thermofisher Scientific	A10520	1:200 in 0.1% PBST
Goat anti-Rabbit Alexa Flour 647	Thermofisher Scientific	A21245	
**Buffers, chemicals, and pharmacological treatments**			
Schneider’s Insect Media (SIM)	Gibco	21720024	1 X
Activation Buffer (AB)	Made in house: 3.3 mM NaH_2_PO_4_, 16.6 mM KH_2_PO_4_, 10 mM NaCl, 50 mM KCl, 5% polyethylene glycol (PEG) 8000, 2 mM CaCl_2_, brought to pH 6.4 with a 1:5 ratio of NaOH:KOH (260 mOsm) (Mahowald et al, 1983)	–	1 X
Dulbecco’s Phosphate Buffered Saline (without CaCl_2_)	Sigma-Aldrich	D8537	1X
1,6 Hexeandiol (1,6-HD)	Sigma-Aldrich	240117	5% in Schneider’s Insect Medium or 1x PBS
2,5 Hexeandiol (2,5-HD)	Sigma-Aldrich	H11904	5% in Schneider’s Insect Medium or 1x PBS
Sodium Chloride (NaCl)	Sigma-Aldrich	S9888	400 mM in H_2_O
NS8593	Sigma-Aldrich	492031	Stock: 100 mM in ethanol Final concentration: 100 µM in Schneiders Insect Medium or Activation Buffer (104)
Household Bleach	ESPO	200-300-0011/1	50% in H_2_O
Paraformaldehyde	Thermofisher Scientific	J19943-K2	4% in PBS
Methanol	32213	Sigma-Aldrich	1 x
Heptane	Thermofisher Scientific	H/0106/PB17	1 x
Triton^TM^ X-100	Sigma-Aldrich	X100	0.2% in PBS
Polyoxyethylenesorbitan Monolaurate (Tween-20)	Sigma-Aldrich	P1379	0.1% in PBS
De-ionised Formamide	Sigma-Aldrich	S4117	10–30% in various FISH buffer solutions
20 X SCC	Sigma-Aldrich	S6639	2X-5X SCC in various FISH buffer solutions
Dextran sulphate	Sigma-Aldrich	D6001	10% in various FISH buffer solution
‘Wash Buffer A’ for Stellaris smFISH	LG Biosearch Technologies	Cat# SMF-WA1-60	20% in de-ionised Formamide and nuclease free H_2_O
‘Hybridisation Buffer’ for Stellaris smFISH	LG Biosearch Technologies	Cat# SMF-HB1-10	90% with de-ionised Formamide
Hybridisation buffer for ImmunoFISH	Made in house (10% Formamide, 2xSSC, Ecoli tRNA (0.36 mg/ml), VRC (2 mM), 5% Dextran sulphate, ultra pure BSA (0.25 mg/ml), stellaris probes, Primary antibody (1:500)		
Tris EDTA (TE) Buffer	Made in house (10 mM Tris-HCl, 1 mM EDTA, pH 8.0)	–	1 x
Slowfade Diamond Antifade Mountant with DAPI	Thermofisher Scientific	S36934	1 X
**Oligonucleotides**			
(1) Custom Stellaris FISH Probes for the 3’UTR of *bcd* RNA labelled with a Quasar 570 (2) Bcd coding region Quasar 670 (3) 32xSunTag Quasar 570	Stellaris	(See appendix table S2-S4 for list of smFISH probes)	Stock: 12.5 μM in TE buffer Final Concentration: 50 nM in 0.25 ml Hybridization buffer
**Software and Algorithms**			
Image J	Schneider et al, 2012	https://imagej.nih.gov/ij/	
R Studio/R Software	R Studio Team 2024	http://www.rstudio.com/	
Graphpad Prism			
**Other**			
Standard Cornmeal Starch Agar FlyFood	Made in house by the Fly Facility, Department of Genetics, University of Cambridge	N/A	N/A
Olympus FV3000 Confocal Laser Scanning Microscope	Olympus	FV3000	N/A

### Generation of SunTag *bcd* transgenic fly lines

**Figure EV3:**
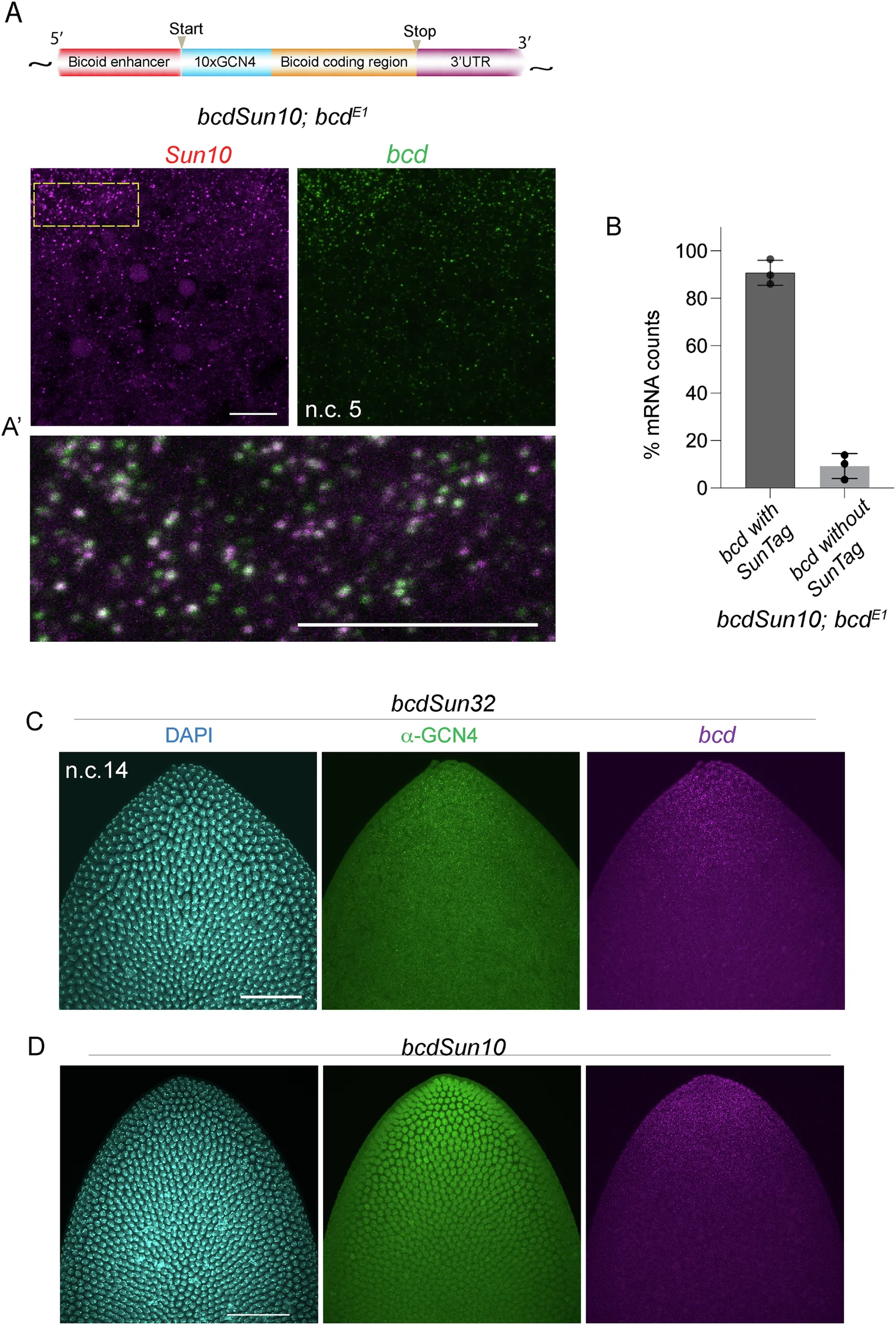
(Related to Fig. 3): *bcdSun10* rescues *bcd* null mutant. (**A**,**A’**) (Top) Cartoon of *bcdSun10* construct. smFISH of SunTag (magenta) and *bcd* (green) regions of *bcdSun10* mRNA localised in the anterior domain of the early blastoderm of homozygous *bcd*^*E1*^ mutant background. Yellow box magnified in (**A**’), showing the highly resolved *bcdSun10* mRNA particles. (**B**) Percentage of colocalisation of *bcd* and SunTag regions in the total *bcd* counts in the embryo’s anterior pole (3 embryos). Error bars are SD. (**C**, **D**) Immuno FISH images of n.c.14 embryo. Embryos from *bcdSun32* (**C**) and *bcdSun10* (**D**) embryos are shown in upper and lower panels, respectively (see Methods section for details). Scale bars: (**A**, **A**ʹ) = 10 µm; (**C**, **D**) = 50 µm.

*bcdSun32* was generated by replacing N-terminal eGFP of eGFP:Bcd-pCaSpeR4 construct (kindly gifted by Thomas Gregor (Gregor et al, 2007b)) by 32xSunTag repeats (kindly gifted by Mounia Lagha (Dufourt et al, 2021)). The 32xSunTag fragments are flanked by NheI and SpeI restriction sites at their N and C-terminal ends, respectively, by PCR, and then ligated into the eGFP:Bcd construct, replacing eGFP. Likewise, the first 10xSunTag repeats of 32xSunTag were ligated in the same way to generate *bcdSun10* construct (Fig. EV3A). Both Bcd SunTag constructs from the donor pCaSpeR4 construct were recombined into w+attB construct (addgene 30326) and flies were generated by phiC31 recombination into attP40 site of chromosome 2. The injection and generation of fly lines were done by BestGene Inc. USA.

The *bcdSun10* fly lines were crossed to the *bcd* null allele mutant, *bcd*^*E1*^. *bcdSun10*;*bcd*^*E1*^ fly lines were viable, suggesting our construct can complement the function of endogenous Bcd. smFISH was carried out to test if *bcdSun10*;*bcd*^*E1*^ effectively replaced the function of endogenous Bcd in the blastoderm. Stellaris smFISH probes with distinct fluorophores were used for SunTag and *bcd* coding regions of the *bcdSun10* mRNA. This enabled us to confirm that the only contribution of maternal *bcd* mRNA was from our *bcdSun10* in the *bcd*^*E1*^ background (Fig. EV3). Colocalisation analysis in the early blastoderm revealed ~90% of the mRNA were accounted for *bcdSun10* (Fig. EV3B). Further, the immunostaining of the blastoderm embryos with anti-GCN4 antibody revealed the translocation of the *bcdSun10* into the nuclei, unlike *bcdSun32*, eventually forming an anterior posterior gradient similar to endogenous Bcd (Fig. EV3C,D).

### Preparation of oocytes

Twenty female flies and ten male flies of the correct genotype were transferred into a vial containing fly food supplemented with yeast and were incubated at 25 °C for 48 h before dissection (Weil et al, 2012a).

### Oocyte dissection

Fattened or aged female flies were anaesthetised, and the posterior tip of the abdomen was removed using a set of fine forceps. The ovaries were then removed from the open posterior end by squeezing the abdomen (Weil et al, 2012a).

### Fly husbandry and preparation of embryos

Fly stocks were maintained at 21 °C in vials that contained standard cornmeal starch agar fly food. To collect flies for experimentation or for genetic crosses, twenty females (virgin females were used for genetic crosses) and ten males were transferred into a plastic bottle containing yeasted cornmeal starch agar fly food at 25 °C. Either virgin females or males from this generation were used for additional genetic crosses, or females younger than 10 days old were selected for experimentation. Collected embryos were dechorionated in 50% bleach for 2 min before excess bleach was removed with distilled water.

### Chemical and pharmacological treatments of oocytes

#### Hexanediol and sodium chloride

Dissected oocytes were incubated for 20 min in 5% 1,6-HD, 5% 2,5-HD, or 400 mM NaCl before immediate fixation. As controls, dissected oocytes were incubated in PBS or SIM for equal amounts of time before fixation.

#### in vitro activation of stage 14 oocytes (fixed)

Dissected stage 14 oocytes were incubated in activation buffer (AB), for 30 min at room temperature. Activated oocytes were incubated in SIM for 0–30 min. To ensure that only activated oocytes were visualised, oocytes were treated with 50% bleach for 2 min. The activated oocytes were then fixed using the embryo fixation method. As a control, oocytes were incubated in SIM for the same time period.

#### Drugs that inhibit the calcium wave

Dissected stage 14 oocytes were incubated in SIM that contained NS8593 for 30 min. The oocytes were then activated in vitro following the same steps as above, with all solutions containing the pre-incubation drug. As a control, oocytes were incubated in SIM (with no AB step) with the drug. Additionally, oocytes were incubated in SIM, followed by AB, followed by SIM with a 1:1000 dilution of either DMSO or ethanol.

### Fixation

Oocytes were incubated 1 ml of 4% paraformaldehyde for 15 min before immediate washing with 0.1% PBST (Abbaszadeh and Gavis, 2016). 10–20 pairs of ovaries were dissected and transferred to room-temperature Schneider’s Insect Medium (SIM). Ovaries were teased apart using dissecting probes until single ovarioles and oocytes were released.

Embryos were transferred into an Eppendorf tube that contains equal parts paraformaldehyde and heptane for 15-30 min, depending on the probes or antibodies used, before the paraformaldehyde phase was removed and replaced with 100% methanol. The sample was then shaken vigorously before all the solution was removed. Embryos were then rinsed with methanol and/or ethanol before being washed in 0.1% PBST (Abbaszadeh and Gavis, 2016).

### Single molecule fluorescence in situ hybridisation (smFISH)

#### Probe design

Custom 20 nucleotide Stellaris FISH Probes were designed against the 3ʹ UTR of *bcd*, coding region of *bcd*, and 32x SunTag by utilising the Stellaris RNA FISH Probe Designer and were the same as designed in (65) (Biosearch Technologies, Inc - www.biosearchtech.com/stellarisdesigner). 32x SunTag and *bcd* 3’UTR Probes were labelled with Quasar 570 and endogenous *bcd* is labelled with Quasar 670 were ordered for smFISH from Biosearch Technologies, Inc (Appendix Tables S2–4).

#### Buffers

Wash buffer was created by mixing the commercially available ‘Wash Buffer A’, de-ionised formamide and nuclease free water in a 2:1:7 ratio or was made using 2% sodium citrate buffer (SSC) + 10% formamide. Hybridisation Buffer was created by mixing ‘Stellaris Hybridisation Buffer’ with de-ionised formamide in a 9:1 ratio.

#### Hybridisation

The hybridisation protocol was adapted from Dufourt et al (2021) and Little et al (2011). Briefly, fixed oocytes or embryos were permeabilised with 0.2% triton X-100 and washed in PBST. When necessary, embryos were blocked in PBST+RNase inhibitor+0.5% ultra pure BSA for 45 min, before being washed in a ‘wash buffer’ solution. The samples were then pre-incubated in hybridisation buffer for 2 h at 37 °C. The hybridisation buffer was removed and replaced by the hybridisation buffer with smFISH probes for overnight incubation at 37 °C, when using anti-GCN4 antibodies in conjunction with smFISH, primary antibodies were incubated overnight in the hybridisation buffer (Bellec et al, 2024). The samples were then washed in hybridisation buffer before repeated washes in prewarmed wash buffer, when using anti-GCN4 antibodies in conjunction with smFISH, secondary antibodies were incubated with the final wash buffer wash, before being washed in 2X SSC. Finally, all samples were washed multiple times in 0.1% PBST.

#### Immunostaining

Nanobody labelling on oocytes or embryos, samples were blocked in a 4% BSA solution and then incubated with nanobodies, either GFP booster or RFP booster, in 0.1% PBST for one hour at room temperature. For anti-GCN4 immunostaining not in conjunction with smFISH, samples were incubated for two hours with the primary antibody, followed by washing with 0.1% PBST and incubation with the secondary antibody for two hours. After incubation, all samples were washed with 0.1% PBST.

Washed samples were transferred to a microscope slide and positioned using a dissecting probe. Excess PBST was removed before a single drop of Slowfade Diamond with DAPI or Prolong Diamond with antifade was added to the sample. A 22 × 22 mm coverslip was lowered onto the sample, and the coverslip was sealed with nail varnish and laid flat in the dark for 24 h before imaging.

#### Imaging

All oocyte imaging was completed on an Olympus FV3000 inverted confocal microscope. Embryo imaging was performed on Olympus FV3000 or Olympus Spinning Disc systems. All images were taken using silicone oil 30x (0.95 NA) or 60x (1.4 NA) objectives. A region of interest was placed around either a whole single oocyte or embryo (30x objective) or a specific region of the oocyte or embryo (60x objective), Images were taken at a resolution of 1024 by 1024, or 2048 by 2048 pixels. 405, 488, 561 and 640 nm lasers were used. Laser power was adjusted to ensure that there was a full range of grey values and that the saturation of pixels was limited.

The images for the quantification of *bcd* foci in the embryo and their colocalisation to translating SunTag, were acquired on an Olympus FV3000 confocal microscope with the following conditions: 60x magnification, 4x zoom, 2 line averaging, slice thickness of 0.4 µm. The regions of high, moderate, and low dense *bcd* foci from the anterior domain and a very low *bcd* region in the posterior were imaged for a total thickness of 10–15 µm.

#### Fixed imaging

Optimised step-size Z-stacks of fixed egg and embryos were collected; all Z-stacks were started at the cortex and extended 10–15 µm into the oocyte or embryo. For colocalisation studies, smFISH, and SunTag translation assays, where maximum resolution was imperative. Imaging parameters were optimised to improve resolution: 60x objective used; pinhole: 1 airy unit; scan speed—400 Hz; 2x line averaging; 2048 by 2048 pixels; and optimised Z-step (300 nm) (Vinter et al, 2021).

### Image analysis

#### Translation quantification

High-resolution image stacks were segmented to identify specific *bcd* foci. We used the trainable Weka segmentation plugin of ImageJ to train and classify the *bcd* foci. A classifier model created from a single image slice was applied to the Z-stack, thus classifying the *bcd* through the whole stack. The segmented particles were analysed using the Fiji particle analysis plugin that provided information on the size, shape, intensity and counts of all particles. The crowded particles that failed to segment properly, which was often the case within highly dense *bcd* regions, were not considered. We also excluded foci with low brightness. The segmented ROIs of *bcd* foci were applied to the anti-GCN4 channel. The mean intensities from anti-GCN4 channel were background corrected, and the positive mean values provided the number of colocalising SunTag to *bcd* particles i.e., these particles were translating *bcd*. The plots were made in Graphpad Prism 8.0.

When automated methods were not accurate for detecting partial colocalisation (Bolte and Cordelieres, 2006), manual analyses were undertaken on images with only partial colocalisation. Images for both channels were thresholded, then an overlay of the threshold outline for channel one (*bcd*) was projected onto the channel two image (anti-GCN4). The number of particles in *bcd* channel that overlapped particles in anti-GCN4 channel was counted, and vice versa. To ensure that this partial colocalisation was not due to chance, images of one channel were inverted and the analysis was completed again.

### Statistical analysis

#### n numbers

10 to 20 females or 30 embryos (biological repeats) were used for each experiment and a global overview of the experimental results was acquired by visualising 20–30 oocytes or 30 embryos for each experimental condition unless stated otherwise. A smaller number of representative oocytes were subjected to high-resolution imaging and analysis as described above. The “n” used in figures represents independent oocyte or embryo samples.

All statistical analyses were performed in R. For all statistical analyses, asterisks represent the following n.s. not significant *P < 0.05, **P < 0.01, ***P < 0.001.

#### Continuous data

Tests for normality were completed before proceeding with the appropriate parametric or non-parametric test. For comparison between two groups, an unpaired Student's t-test (STT) or a Wilcoxon signed-rank test (WSRT) was used.

#### Categoric data

A χ^2^ test (CST) or Fisher’s exact test (FET) was used to determine if there were significant differences between groups. This depended on sample size Fisher’s exact test is preferred for small sample sizes (< 50).

### SDD model

Equations 1–2 were solved in Matlab using custom code as developed in Athilingam et al (2024) and available on the laboratory Github: https://github.com/TimSaundersLab. Here, we included spatially and temporally varying production. This was implemented as step functions at initiation (t = 0). The spatial domain was defined by an exponentially decaying function that matched our experimental observation of translation. Parameters were constrained by experimental measurements, with +20% variation allowed about measured means in parameter fitting. The only exception was β, for which we do not have direct measurements. We provide in the Supplementary Information model output when β decreases at later nuclear cycles.

### Fly lines

Appendix Table S1 outlines all lines used in this study.

## Supplementary information

Appendix

Peer Review File

Movie EV1

Expanded View Figures

## Data Availability

Source data is available in BioImage Archive with accession number S-BIAD3977. The source data of this paper are collected in the following database record: biostudies:S-SCDT-10_1038-S44318-026-00927-5.
