# Supplementary material for: Dynamics of bicoid mRNA localisation and translation dictate morphogen gradient formation
Source: EMBO J. 2026 Oct 10;45(21):7374–404. doi: 10.1038/s44318-026-00927-5 (PMC13728150; doi:10.1038/s44318-026-00927-5)
Supplement: Supplementary file 1 [file 44318_2026_927_MOESM1_ESM.pdf]

Appendix

Dynamics of *bicoid* mRNA localisation and translation dictate morphogen gradient formation

Contents:

Page 2: Appendix Table S1
Page 3: Appendix Table S2
Page 4: Appendix Table S3
Page 5: Appendix Table S4
Page 6: Appendix Table S5
Page 7: Appendix Table S6
Page 8-9: Appendix Table S7
Page 10-11: Appendix Table S8
Page 12: Appendix Table S9

Fly line & genotype	Source and identifier
**Visualisation of proteins**	
**Me31B::GFP**<br>(Endogenous Tag)	(93)<br>BDSC: 51530
**Suntag-Bcd**<br>W; 32xSuntag-Bcd/ Cyo<br>W;10xSunTag-Bcd;bcd^{E1}	Generated here by Saunders lab
**ScFv-msGFP2**<br>nos E/Pr > Sc-FV-msGFP2 (with NLS)	Gift from Mounia Lagha<br>(91)
**ScFv-mNeonGreen**<br>nos E/Pr > Sc-FV-mNeonGreen (no NLS)	Gift from Hilary Ashe
**Mutations and RNAis**	
**endos 1**<br>P(94); $endos^{1}$ /TM3, Sb	Gift from Hélène Rangone<br>(95)
**endos EY01103**<br>P(94); $endos^{EY01103}$ /TM3, Sb	Gift from Hélène Rangone<br>(95)
**endos EY01105**<br>P(94); $endos^{EY01105}$/TM3, Sb	Gift from Hélène Rangone<br>(95)

Appendix Table S1: Summary of the *Drosophila* lines used in this work.

smFISH probes
5'-GAAACTCTCTAACACGCCTC-3'
5'-ACAGTGGTTAACCTAAAGCT-3'
5'-TGGTATTTGTACAATCAGGA-3'
5'-CTTTCTACGCGTAGATATCT-3'
5'-ACGGATCTTAGGACTAGACC-3'
5'-AAACTTCCCTGGGAACCATT-3'
5'-CTGCTGACTAGGCTAGTACA-3'
5'-GATATGCACTGGAATCCGTG-3'
5'-GAGTTAACTGGAGTATCACT-3'
5'-AGCGTATTGCAGGGAAAGTA-3'
5'-CACCCAGATACATCTAAGGC-3'
5'-CATATTCCCGGGCTTTAGTG-3'
5'-TGGCCTCAAATGTAACTGGT-3'
5'-ACTTTCCATGGAATACGCTT-3'
5'-ATTTCCGAAATGTGGGACGA-3'
5'-AGAAGATTTTCTTGCTGGCT-3'
5'-GTACAGTTTTTAGCTATGTC-3'
5'-ATGAGATTACGCCCAAGAGA-3'
5'-ATGTTCGATCTTTAAGGGTA-3'
5'-ACACTTTGGCATAGCATAGA-3'
5'-GCGCAAATGTTTGATTATGT-3'
5'-TTGCTGACTATTCTTGGTCA-3'
5'-ACAAATGGTCTGCATTGATT-3'
5'-TGATAGTTATTCCGTTTGGC-3'
5'-ATGCTCTTCTTAGTGATGTA-3'
5'-ACTTGAGGCCTAACAGATTG-3'
5'-ACAACATCAAAGGTGCAGCA-3'
5'-ATTTACCCGAGTAGAGTAGT-3'

Appendix Table S2: Custom Stellaris® FISH probes for the 3'UTR of *bcd* RNA (46).

smFISH probes 32XSUNTAG
5’- AGTCCTCTTCTTGAAGAGAG-3’
5’- ATCTCTTACTTCATCGAGCT -3’
5’- AGTCCACTTCTCGAGAATAG -3’
5’- AGAACTTTTACTCCATCGCT -3’
5’- AGACCACTTCTTAACGAGAG -3’
5’- GATAGTGGAGCTCTTGCTTC -3’
5’- CACCTCTTCTTGAAGACAGA -3’
5’- TTAATGGTGGATCTCTTGCT -3’
5’- CCACTTCTTAACGAGAGGTT -3’
5’- GTAAACCTTTTACTTCACCG -3’
5’- GTCGTTCTTGATAGTAGACC -3’
5’- TTCATCGCTCTGACTTTTTT -3’
5’- CGCTTCTTAACGAGAGGTTT -3’
5’- ACCTCTTCTCAATGACTCAT -3’
5’- AATCTTTTACTCCAGCGTTC -3’
5’- TGAGGACAGATTCTTGATGG -3’
5’- GTCTGAATTTTTTCCCTCAC-3’
5’- AGGTTCTTGATGGTAGAGCT-3’
5’- GATCCGACTTTTTTCCAAGA -3’
5’- CGACGAGAGATTTTTGATGG -3’
5’- CTTAATGGTAGACCTCTTGC -3’
5’- CCAAGGCCACTTCTTGATAA -3’
5’- AATGGTAGAGCTCTTACTCC -3’
5’- CTCTTCTCAACGAGAGGTTT -3’
5’- CTTCAACGCGCTGAATTCTT -3’
5’- CTCTTCTTGACAACTCGTTT -3’
5’- ATGGTAGAGCTTTTACTCCA -3’
5’- TGGTAGAGCTCTTACTTCAC -3’
5’- AGAGCTCTTACTTCAACGTG -3’
5’- GTGAACCTTTTGCTTCAACG -3’
5’- TCTTGATAGTGGATCTCTTG -3’
5’- CCATCACCACTTCTTAATGA -3’
5’- CTCTCCTCGATAACTCATTT -3’
5’- TAAATCTCTTACTTCACCGG -3’
5’- GGCCTCTTCTCAACAACAGT -3’
5’- TGATTTCTTTCCAAGTCCTA -3’
5’- CTTCTCGAGGAGAGTTTCTT -3’
5’- CCGCTTCTTAACGAGAGATT -3’
5’- CTAATTTTTTTCCATCACCC -3’
5’- CTCCTTGATGAGAGGTTTTT -3’
5’- TTCGCCACTCCTTAATGAAA -3’
5’- AGTGGAGCTTTTGCTTCAAC -3’
5’- GGCCACTTCTCGATAATAGA -3’
5’- ATCTAATTTTTTCCCATCGC-3’
5’- ACGATTCATTCTTGATGGTG -3’
5’- GAATTTTTTCCCTAGACCTT -3’
5’- TAGTGGAGCTCTTGCTTCAA -3’
5’- TCTCTTACTTCATCGTTCTA -3’

Appendix Table S3: Custom Stellaris® FISH probes for the 32xSunTag designed through stellaris probe designer version 4.2.

smFISH probes bcd coding region
5’-GGCAGCGGATGATGGTAAAA-3’
5’-AACTGAAGCTGCGGATGTTG-3’
5’-AGGGATTTCGGAATTGTGGC-3’
5’-CGTTCGCTCATCGAAAAGCA-3’
5’-TGTAGTTGTAGTTTATCGCT-3’
5’-CATCTGGTTGGGCAGATACG-3’
5’-GAGGGAAAGACATCTGGCTT-3’
5’-TAAAAGTGGTGCGGGTGCGA-3’
5’-CAGCTCTGCTATTTGAGAGC-3’
5’-TATCGTCCCTGCAGAAAGTG-3’
5’-GCTAGTTTCGCTGACAGATC-3’
5’-ATATCTTCACCTGGGCTGTG-3’
5’-CGACGCCGACGGTTCTTAAA-3’
5’-CTGATCCGATTGGATCTTGT-3’
5’-TTTCATACCCGGCGAGAGAG-3’
5’-AGCTAAGAGTCTGCAAGCTG-3’
5’-GTGACGGAGTCAAAGCGTTG-3’
5’-TGAATGACTCGCTGTAGTGC-3’
5’-TCCATTGTAGTTGTAGTAGG-3’
5’-CATGTGCATGTGACGATTGG-3’
5’-ATTGACATTGGTCGACCCAG-3’
5’-TTGCTTTTGCTGGAAGTCAA-3’
5’-TTGAAGTTGTAGTCGGCCTC-3’
5’-CGATCGCATGTAGTACGAGC-3’
5’-GGTGTTAATGGCTCGTAGAC-3’
5’-AGACTCGGACTTTCGTCATT-3’
5’-AAGATCTGTAGCGTCGTCTT-3’
5’-CTTGTCCAGACCCTTCAAAG-3’
5’-TTCCTGCTAAGGCTCTTATT-3’
5’-AAATGCCGCTCCACGATTTC-3’
5’-GCGAAGGCTTGCCAAATTTG-3’
5’-TGATATTGGTTCGATTCGCC-3’
5’-TTGCATTATCGTATCCATCG-3’
5’-CGTTCCGATGGGGATTATAC-3’
5’-AGTAGGCAAACTGCGAGTTG-3’

Appendix Table S4: Custom Stellaris® FISH probes for the bcd coding region designed through stellaris probe designer version 4.2.

Parameter	Description	Value	Reference
$\alpha = \alpha_0 + \alpha_1 \cdot \frac{t^n}{t^n + t_0^n}$	*bcd* degradation rate	$\alpha_0 = 1/180$ *min*$^{-1}$<br>$\alpha_1 = 1/10$ *min*$^{-1}$<br>$n = 12,\ t_0 = 120min$	This work and (58)
$\beta \frac{t^m}{t^m + t_1^m}$	*bcd* translation rate	$\beta = 10^{-2}s^{-1}$<br>$m = 4,\ t_1 = 10min$	Fitted
$\beta \frac{t^m}{t^m + t_1^m} \cdot \frac{t_2^p}{t_2^p + t^p}$	*bcd* translation rate, with reduction in n.c. 13	$\beta = 10^{-2}s^{-1}$<br>$m = 4,\ t_1 = 10min$<br>p = 4, $t_2 = 130min$	Fitted
$D(x) = D_0 + D_1 x/L$	Bcd diffusivity	$D_0 = 4\mu m^2 s^{-1}$<br>$D_1 = 3\mu m^2 s^{-1}$	(49, 97)
D_{sdd}	Bcd diffusivity in SDD model	$D_{sdd} = 4\mu m^2 s^{-1}$	(49, 97)
L	Embryo length	$500\mu m$	
μ	Bcd degradation rate	$1/35$min^{-1}	(10)
$f(x)$	Initial *bcd* distribution	Figure 7	This work

Appendix Table S5: Model parameters used in Figure 7 and Figure S9.

Domain-Dorsal surface	Density of *bcd* particles	Sample No./stage	Total *bcd* particles counted	Translated (colocalized to GCN4)	Non-translated	% translated
Anterior	High dense (within 50µm from the anterior pole)	1/n.c.3	8602	8138	464	94.6
		2/n.c.3	8409	8000	409	95.1
		3/n.c.3	5544	4855	689	87.6
	Moderately dense (50 to100 µm from the anterior pole)	1/n.c.3	7351	7174	177	97.6
		2/n.c.3	6846	5551	297	81.1
		3/n.c.3	2720	2517	203	92.5
	Low (~150-200 µm from the anterior pole)	1/n.c.3	197	181	16	91.9
		2/n.c.3	683	536	147	78.5
		3/n.c.3	447	312	135	69.7
Posterior	Very low (~50 µm from the posterior pole)	1/n.c.3	6	2	4	33.3
		2/n.c.3	0	0	0	0
		3/n.c.3	8	2	6	25

Appendix Table S6: Translation of *bcd* in the early Drosophila embryo (related to Figure 3C,C'). The absolute counts of translating *bcd* and their percent translation at the embryo' dorsal surface up to a depth of approximately 20-30µm at four positions along the A-P axis.

Distance from the dorsal surface (μm)	Anterior domain					
	Very dense		Moderately Dense		Low	
	Total *bcd* particles counted	Translated (colocalized to GCN4)	Total *bcd* particles counted	Translated (colocalized to GCN4)	Total *bcd* particles counted	Translated (colocalized to GCN4)
0.0	0	0	0	0	0	0
0.4	0	0	0	0	1	1
0.8	0	0	0	0	4	2
1.2	0	0	2	2	60	45
1,6	60	58	47	43	54	43
2.0	292	267	372	360	81	59
2.4	383	359	447	429	60	42
2.8	418	402	525	505	56	43
3.2	667	655	601	580	68	50
3.6	582	564	491	465	26	16
4.0	388	373	553	533	45	41
4.4	767	729	389	367	36	31
4.8	611	551	323	309	33	27
5.2	280	273	393	381	32	24
5.6	466	454	275	260	15	11
6.0	374	365	271	260	26	23
6.4	379	355	175	164	12	9
6.8	357	336	151	139	13	10
7.2	283	264	123	113	13	12
7.6	288	280	115	103	4	4
8.0	260	246	77	74	7	7
8.4	232	213	92	90	6	6
8.8	177	167	78	70	3	3
9.2	182	173	33	31	3	3
9.6	133	126	29	28	1	1
10.0	113	105	39	36	6	6
10.4	133	127	10	10	0	0
10.8	95	88	15	15	1	1
11.2	97	93	22	20	1	1
11.6	94	86	12	10	0	0
12.0	69	69	18	18	3	3
12.4	75	74	13	13	0	0
12.8	56	55	8	5	0	0
13.2	42	41	12	10	0	0
13.6	56	52	17	14	0	0
14.0			5	4	0	0
14.4			11	8	2	2

14.8			19	12	2	2
15.2			9	7	1	1
15.6			7	7	2	2
16.0			9	7	1	1
16.4			3	3	1	1
16.8			4	4	2	2
17.2			10	7	0	0
17.6			1	0	2	1
18.0			4	4		
18.4			5	4		
18.8			3	3		
19.2			3	3		
19.6			8	5		
20.0			2	2		
20.4			4	3		
20.8			5	4		
21.2			2	2		
21.6			3	2		
22			3	3		

Appendix Table S7: Translation of *bcd* in the early Drosophila embryo (related to Figure S5C-E) The absolute counts of the total *bcd* and the translating *bcd* at the embryo' dorsal surface up to a depth of approximately 20-30μm at three positions in the anterior domain.

Anterior domain (within 50µm from the anterior pole)						
Stages	samples	relative to nuclear position	Total *bcd* counts	Translated *bcd* counts	Non-translated	% translated
n.c.3-4	1		8602	8138	464	94.6
	2		8409	8000	409	95.1
	3		5544	4855	689	87.6
n.c.7-8	1		650	609	41	93.7
	2		993	836	157	84.1
	3		2039	1930	109	94.6
n.c.10-11	1	Apical	475	330	145	70
		Basal	1028	934	94	91
	2	Apical	1354	1219	135	97
		Basal	901	768	133	85
	3	Apical	713	642	71	90
		Basal	1468	1336	132	91
n.c.12	1	Apical	250	156	94	62.4
		Basal	400	271	129	67.8
	2	Apical	682	569	113	83.4
		Basal	1872	1689	183	90
	3	Apical	557	502	55	90
		Basal	1883	1711	174	90.9
n.c.13	1	Apical	338	168	170	49.7
		Basal	150	98	52	65.3
	2	Apical	741	402	339	54.2
		Basal	1501	675	826	44.9
	3	Apical	435	317	118	72.8
		Basal	1061	937	124	88.3
Early n.c.14	1	Apical	261	91	170	34.9
		Basal	183	67	116	36.6
	2	Apical	427	153	274	35.8
		Basal	338	64	274	18.9
	3	Apical				
		Basal				
Late n.c.14	1	Apical	282	42	240	15

		Basal	63	0	63	0
	2	Apical	82	1	81	1
		Basal	136	0	136	0
	3	Apical	216	3	213	1.3
		Basal	255	3	252	1.2

Appendix Table S8: Translation of *bcd* in the anterior domain from the early *Drosophila* blastoderm to the end of n.c.14 (related to Figure 4).
The absolute counts of the total *bcd* , translating *bcd* , and the non-translating *bcd* at the anterior domain of the embryo's dorsal surface.

Distance A-P axis (μm²)			50	100	150	200	250	300	350	400	450	500
n.c.3-4	Sample1	total *bcd* particles	5643	3317	184	16	3	5	2	1	0	0
		bcd Translated	5247	3249	174	12	3	4	1	0	0	0
	Sample2	total *bcd* particles	5772	4549	566	81	0	2	12	2	0	0
		bcd Translated	5600	4367	533	71	0	2	10	2	0	0
	Sample3	total *bcd* particles	979	1259	165	4	0	1	1	1	0	0
		bcd Translated	876	1096	126	3	0	1	1	0	0	0
n.c.13	Sample1	total *bcd* particles	1394	467	203	14	2	0	0	0	0	0
		bcd Translated	1094	419	176	14	2	0	0	0	0	0
	Sample2	total *bcd* particles	401	680	48	1	0	0	0	0	0	0
		bcd Translated	267	350	23	1	0	0	0	0	0	0
	Sample3	total *bcd* particles	2661	2639	248	284	10	2	0	0	0	0
		bcd Translated	1320	1145	147	261	7	0	0	0	0	0
n.c.14	Sample1	total *bcd* particles	1181	824	59	10	6	0	0	0	0	0
		bcd Translated	870	530	37	5	4	0	0	0	0	0
	Sample2	total *bcd* particles	897	183	23	3	0	0	0	0	0	0
		bcd Translated	434	118	15	1	0	0	0	0	0	0
	Sample3	total *bcd* particles	1762	2601	445	57	5	2	0	0	0	0
		bcd Translated	845	752	171	18	1	1	0	0	0	0

Appendix Table S9: Translation of *bcd* across the embryo length from the early *Drosophila* blastoderm to the end of n.c.14 (related to Figure 4).

The absolute counts of the total *bcd* and the translating *bcd* in the increment of 50μm² from the anterior to the posterior end of the embryo's dorsal surface.
