# Supplementary material for: Dynamics of bicoid mRNA localisation and translation dictate morphogen gradient formation
Source: EMBO J. 2026 Oct 10;45(21):7374–404. doi: 10.1038/s44318-026-00927-5 (PMC13728150; doi:10.1038/s44318-026-00927-5)
Supplement: Supplementary file 2 [file 44318_2026_927_MOESM2_ESM.pdf]

THE EMBO JOURNAL

Dynamics of *bicoid* mRNA localisation and translation dictate morphogen gradient formation

Thamarailingam Athilingam, Elise Wilby, Pierre Bensidoun, Antonio Trullo, Morgane Verbrugghe, Jérémy Dufourt, Xingzhu Shi, Mounia Lagha, Timothy Saunders, and Timothy Weil

Corresponding authors: Timothy Weil (tw419@cam.ac.uk) , Timothy Saunders (timothy.saunders@warwick.ac.uk)

Review Timeline:

Transferred from Review Commons:	25th Jan 26
Editorial Decision:	30th Jan 26
Revision Received:	15th May 26
Editorial Decision:	17th Jun 26
Revision Received:	9th Jul 26
Accepted:	26th Aug 26

Editor: Ieva Gailite

Transaction Report:

Review COMMONS

This manuscript was transferred to The EMBO Journal following peer review at Review Commons.

(Note: With the exception of the correction of typographical or spelling errors that could be a source of ambiguity, letters and reports are not edited. Depending on transfer agreements, referee reports obtained elsewhere may or may not be included in this compilation. Referee reports are anonymous unless the Referee chooses to sign their reports.)

Review #1

1. Evidence, reproducibility and clarity:

Evidence, reproducibility and clarity (Required)

In this paper the authors use the Suntag system to visualise bcd mRNA translation in the Drosophila embryo. They elucidate the relationship between bcd mRNA translation and P body localisation. In the oocyte, bcd mRNAs are localised in P bodies and translationally repressed, but upon egg activation bcd mRNAs are released from P bodies and translated. In addition, during mid-nc14, bcd mRNAs become localised to embryonic P bodies and degraded. The authors use their data to modify the Synthesis, Diffusion, Degradation model of Bcd gradient formation, which recapitulates the Bcd gradient detected experimentally.

Overall, I think the data are of high quality and support the authors' conclusions. I only have minor comments, as follows:

Fig 1B - add arrows showing mRNAs being translated or not (the latter mentioned in line 113 is not so easy to see).

Fig 2A - add a sentence explaining why 1,6HD, 2,5HD and NaCl disrupt P bodies.

Fig 4C - explain in the legend what the white lines drawn over the image represent. And why is there such an obvious distinction in the staining where suddenly the DAPI is much more evident (is the image from tile scans)?

Line 215 - 'We did not see any significant differences in the translation of bcd based on their position, however, there appears an enhanced translation of bcd localised basally to the nuclei (Figure S5).' Since the difference is not significant, I do not think the authors should conclude that translation is enhanced basally.

Line 218: 'The interphase nuclei and their subsequent mitotic divisions appeared to displace bcd towards the apical surface (Figure S6B).' Greater explanation is needed in the legend to Fig S6B to support this statement as the data just seem to show a nuclear division - I would have thought an apical-basal view is needed to conclude this.

Fig 5B - the authors compare Bcd protein distribution across developmental time. However, in the early time points cytoplasmic Bcd is measured (presumably as it does not appear nuclear until nc8 onwards) and compare the distribution to nuclear Bcd intensities from nc9 onwards. Is most/all of the Bcd protein nuclear localised form nc9 to validate the nuclear quantitation? Does the distribution look the same if total Bcd protein is measured per volume rather than just the nuclear signal? Are the authors assuming a constant fast rate of nuclear import?

Line 259 - 'We then asked if considering the spatiotemporal pattern of bcd translation' - the authors should clarify what new information was included in the model. Similarly in line 286,

'By including more realistic bcd mRNA translation' - what does this actually mean? In line 346, 'We see that the original SDD model .... was too simple.' It would be nice to compare the outputs from the original vs modified SDD models to support the statement that the original model was too simple.

Fig S1A - clarify what the difference is between the 2 +HD panels shown.

Fig S2E - the graph axis label/legend says it is intensity/molecule. Since intensity/molecule is higher in the anterior for bcd RNAs, is this because there are clumps of mRNAs (in which case it's actually intensity/puncta)?

Fig S4 - I think this line is included in error: '(B) The line plots of bcd spread on the Dorsal vs. Ventral surfaces.'
In B, D, E - is the plot depth from the dorsal surface? I would have preferred to see actual mRNA numbers rather than normalised mRNAs. In Fig S4D moderate, from 10um onwards there are virtually no mRNA counts based on the normalised value, but what is the actual number? The equivalent % translated data in Fig S4E look noisy so I wonder if this is due to there being a tiny mRNA number. The same is true for Figs S4D, E 10um+ in the low region.

Referees cross-commenting

I think the concerns raised by reviewers 2 and 3 are valid, and that it is feasible for the authors to address all the reviewers' concerns in order to improve the manuscript.

2. Significance:

Significance (Required)

General assessment

Strengths are: 1) the data are of high quality; 2) the study advances the field by directly visualising Bcd mRNA translation during early Drosophila development; 3) the data showing re-localisation of bcd mRNAs to P bodies nc14 provides new mechanistic insight into its degradation; 4) a new SDD model for Bcd gradient formation is presented. Limitations of the study are: 1) there was already strong evidence (but no direct demonstration) that bcd mRNA translation was associated with release from P bodies at egg activation; 2) it is not totally clear to me how exactly the modified SDD model varies from the original one both in terms of parameters included and model output.

Advance

The advance is conceptual, technical and mechanistic.

Audience

The results will be important to a broad range of researchers interested in the formation of

developmental morphogen gradients and the post-transcriptional regulation of gene expression, particularly the relationship with P bodies.

My expertise

Wetlab developmental biologist

3. How much time do you estimate the authors will need to complete the suggested revisions:

Estimated time to Complete Revisions (Required)

(Decision Recommendation)

Less than 1 month

4. *Review Commons* values the work of reviewers and encourages them to get credit for their work. Select 'Yes' below to register your reviewing activity at Web of Science Reviewer Recognition Service (formerly Publons); note that the content of your review will not be visible on Web of Science.

Web of Science Reviewer Recognition

Yes

Review #2

1. Evidence, reproducibility and clarity:

Evidence, reproducibility and clarity (Required)

In this manuscript by Athilingam et al., the authors are studying the translation of the morphogen Bicoid (Bcd), which is in anterior-posterior patterning of the blastoderm Drosophila embryo. They have used an array of sunTag elements in the 5' UTR of Bcd to detect the localization of translation. They found that, not only is Bcd not translated until egg activation, but it can only be translated at the anterior pole, even though bcd mRNA has a broader spatial distribution.

In general, the paper uses a cutting-edge methodology to address one of the foundational questions of the best-studied morphogen gradient: namely, what is the spatial distribution of the Bcd source? Together with the dynamics of its spreading (which they addressed in a separate study in 2024) and Bcd degradation, their results point to a modified form of the synthesis/diffusion/degradation (SDD) model of Bcd gradient formation, which they have analyzed in the final subsection of the results. However, there are several major issues that erode the validity and impact of the paper, most of which can be put into the category of vague explanations, missing information, or contradictory statements, making it hard to understand/verify what conclusions can be drawn. This is also coupled with vague figures and captions. We describe these, and a few minor issues, in detail below:

- Line 114: The authors claim to have validated the SunTag using a fluorescent reporter, but do not show any data. Ref 60 is a general reference to the SunTag, and not the Bcd results in this paper. Perhaps place their data into a supplemental figure or movie?
- Line 128 and Fig. 1E: The claim that bcd becomes dispersed is difficult to verify by looking at the image. The language could also be more precise. What does it mean to lose tight association? Perhaps the authors could quantify the distribution, and summarize it by a length scale parameter? This is one of the main claims of the paper (cf. Line 23 of the abstract) but it is described vaguely and tersely here.
- Line 146, Fig. 1G: This is a really important figure in the paper, but it is confusing because it seems the authors use the word "translation," when they mean "presence of Bcd protein." In other places in the paper, the authors give the impression that "bcd translation" means translation in progress (assayed by the colocalization of GCN4 and bcd mRNA). However, in Fig. 1G, the focus is only on GCN4. Detecting Bcd protein only at the anterior does not mean that translation happens only at the anterior (e.g., diffusion or spatially-restricted degradation could be in play).

It would also be helpful to show a plot with quantification of Bcd detection (or translation) on the y-axis and a continuous AP coordinate on the x-axis, instead of just two points (anterior and posterior poles, the latter of which is uninteresting because observing no Bcd at the posterior pole is expected).

Another issue with Fig. 1G is that the A and P panels presumably have different brightness and contrast. If not, just from looking at the A and P panels, the conclusion would be that Bcd protein is diffuse (and abundant) in the posterior and concentrated into puncta in the anterior. The authors should either make the brightness and contrast consistent or state that the P panel had a much higher brightness than the A panel.

- Line 176: This section is very confusing, because at this point the authors already addressed the spatial localization of translation in Fig. 1G,H (see my above comment). However, here it seems the authors have switched the definition of translation back to "translation in progress." Therefore, the confusion here could be eliminated by addressing the above point.
- Line 185: The sentence here is seemingly contradictory: "most...within 100 microns" implies that at least some are beyond 100 microns, while the sentence ends with "[none]...more than 100 microns." The language could perhaps be altered to be less vague/contradictory.
- Line 204: It would be really nice to have quantification of the translation events, such as curves of rate of translation as a function of a continuous AP coordinate, and a curve for each nc.

- Line 209 and Fig 4C: The authors use the terms "intensity of translation events" or "translation intensity" without clearly defining them. From the figure (specifically from the y-axis label), it looks like the authors are quantifying the intensity per molecule (which is not clearly the same thing as "translation intensity"), but it would be nice if that were stated explicitly.

In addition, the authors again quantify only two points. This is a continuously frustrating part of the manuscript, which applies to nearly all figures where the authors looked only at two points in space. At a typical sample size of N = 3, it seems well within time constraints to image at multiple points along the AP axis.

Furthermore, it sounds like the authors are saying the "translation intensity" is the same in anterior and the posterior, which is counterintuitive. The expectation is that translation would be undetectable at the posterior end, in part because bcd mRNA would not be present. (Note that this expectation is even acknowledged by the authors on Line 185, which I comment on above, and also on Line 197). There should also be very low levels of Bcd protein (possibly undetectable) at the posterior pole. As such, the authors should explain how they think their claim of the same "translation intensities" in the anterior vs posterior fits into the bigger picture of what we know about Bcd and what they have already stated in the manuscript. They should also explain how they observed enough molecules to quantify at the posterior end. The authors should also disclose how many points are in each box in the boxplot. For example, the sample size is N = 3 embryos. In just three embryos, how many bcd/GCN4 colocalizations did the authors observe at the posterior end of the embryo?

- Line 215: The sentence that starts on this line seems self-contradictory: I cannot tell whether or not there is a difference in translation based on position.
- Line 229: Long-ranged is a relative term. From the graph, one could state there is some spatial extent to the mRNA gradient, so it is unclear what the authors mean when they say it is not "long-ranged." Could the mRNA gradient be quantified, such as with a spatial length scale? This would provide more information for readers to make their own conclusions about whether it is long-ranged.
- Line 230: When the authors claim the Bcd gradient is steeper earlier, a quantification of the spatial extent (exponential decay length scale) would be appropriate. Indeed, lambda as a function of time would be beneficial. It should also be placed in context of earlier papers that claim the spatial length scale is constant.
- Lines 235-236: The two sentences that start on these two lines are vague and seemingly contradictory. The first sentence says there is a spatial shift, but the second sentence sounds like it is saying there is no spatial change. The language could be more precise to explain the conclusions.

Minor issues/typos (still must be addressed for content):

- Line 81: Probably meant "evolutionarily conserved"
- Figure 1 legend: part B says "from 15 samples" but also says N = 20. Which is it, or do these numbers refer to different things?
- Line 217: migration of what?

- Line 228: "early embryo" is vague. The authors should give specific time points or nuclear cycle numbers.
- Line 301: Other locations in the paper say 75 microns or 100 microns.
- Fig. 5: all images should be oriented such that the dorsal midline is on the upper half of the embryo/image.
- Fig. 5B: There are light tan and/or light orange curves (behind the bold curves) that are not explained.
- Fig. 5C: the plot says "normalized" but nowhere do the authors describe what the curves are normalized to. There is also no explanation for what the broad areas of light color correspond to.

Referees cross-commenting

This is Reviewer 2. Yes, I am enthusiastic about the work: it is a much needed set of experiments and it fits well into the overall goal of quantitatively understanding the processes that establish the Bcd gradient. My main concern(s) about this paper is the loose and vague way they described their experiments and the interpretations. My hope is they will use the revision as an opportunity to more precisely explain their work.

Other than that, I am in agreement with the other reviewers on the need to revise for clarity and publish this important work.

2. Significance:

Significance (Required)

The results, if upheld, are highly significant, as they are foundational measurements addressing a longstanding question of how morphogen gradients are formed, using Bcd (the foundational morphogen gradient) as a model. They also address fundamental questions in genetics and molecular biology: namely, control of mRNA distribution and translation.

3. How much time do you estimate the authors will need to complete the suggested revisions:

Estimated time to Complete Revisions (Required)

(Decision Recommendation)

Between 1 and 3 months

4. *Review Commons* values the work of reviewers and encourages them to get credit for their work. Select 'Yes' below to register your reviewing activity at Web of Science

Reviewer Recognition Service (formerly Publons); note that the content of your review will not be visible on Web of Science.

Web of Science Reviewer Recognition

Yes

Review #3

1. Evidence, reproducibility and clarity:

Evidence, reproducibility and clarity (Required)

This is a review of "Dynamics of bicoid mRNA localization and translation dictate morphogen gradient formation" by Athilingam et al. In this manuscript, the authors perform quantification of mRNA localization and translation of bicoid, spanning oogenesis through the maternal to zygotic transition, yielding a definitive characterization of Bicoid gradient formation. The experiments, analysis, and interpretation are on the whole performed rigorously. I very much enjoyed this paper, partly for incorporating the aspects of bcd regulation during oogenesis, which compared to embryonic function of bcd is relatively under-studied. Also valuable is improving the characterization of how bcd expression is shut down at NC14. I have several major comments for revision, and a few minor comments. I should stress that none of the major comments are terrible but are intended to improve the impact/readability/flow of this nice manuscript. With the exception of a straightforward immunostaining experiment, all major comments constitute reworking of the model or the text.

Major Comments:

1) It is not evident from the main results and methods text that the new SDD model incorporates the phenomenon reported in figure 4B. From my reading, the parameter beta accounts for the Bcd translation rate, which according to figure 7B(ii) effectively switches from off to on around fertilization and thereafter remains constant. Figure 4B shows that the fraction of bcd mRNA engaged in translation decreases beginning around NC12/13, and this is one of the more powerful results that comes from monitoring translation in addition to RNA localization/abundance/stability. My expectation based on figure 4B would be that parameter beta should decrease over time beginning around 90-100 minutes and approach zero by ~150 minutes. This rate could be fit to the experimental data that yields figure 4B. The modeling should be repeated while including this information.

2) The presentation of the SDD model should be expanded to address how well the characteristic decay length fits A) measured Bcd protein distributions, B) measured at different nuclear cycles.

This would strengthen the claim that the new SDD model better captures gradient dynamics given the addition of translation and RNA distribution. These experimental data already exist as reported in Figure 5. In the current Figure 7, panels D and D' add little to the story and could be moved to a supplement if the authors want to include it (in any case, please fix the typo on the time axis of fig 7D' to read "hours"). The model per cell cycle and the comparison of experimental and modeled decay lengths could replace current D and D'.

3) The exposition of the manuscript would benefit significantly by including a section either in the introduction or the appropriate section of the results that defines the competing models for gradient formation. In the current version, these models are only cited, and the key details only come out late (e.g., lines 302 onward, in the Discussion). Nevertheless, some of the results are presented as if in dialog with these models, but it reads as a one-sided conversation. For instance: Figure 3. The undercurrent in this figure is the RNA-gradient model. In the context of this model, the results clearly show that translation of bcd is restricted to the anterior. Without this context, Figure 3 could read as a fairly unremarkable observation that translation occurs wherever there is mRNA. Restructuring the manuscript to explicitly name competing models and to address how experimental results support or detract from each competing model would greatly enhance the impact of the exposition.

4A) Related to point 3: The entire results text surrounding Figure 2 should be revised to include more detail about A) what specific hypotheses are being tested; and B) to critically evaluate the limitations of the experimental approaches used to evaluate these hypotheses. Hexanediol and high salt conditions are not named explicitly in the text, but the text touts these as "chemicals" that "disrupt P-body integrity." This implies that the treatments are specific to P-bodies. Neither of these approaches are only disrupting P Body integrity. This does not invalidate this approach, but the manuscript needs to state what hypothesis HD and NaCl treatment addresses, and acknowledge the caveats of the approach (such as the non-specificity and the assumptions about the mechanism of action for HD).

4B) Continuing the comment above: it is good that the authors checked that HD and NaCl treatment does not cause egg activation. But no one outside of the field of Drosophila egg activation knows what the 2-minute bleach test is and shouldn't have to delve into the literature to understand this sentence. Please explain in one sentence that "if eggs are activated, then x happens following a short exposure to bleach (citations). We exposed HD and NaCl treated eggs to bleach and observed... ."

4C) Continuing the comment above: The section of the results related to the endos mutation needs additional information. It is not apparent to the average reader how the endos mutation results in changes in RNP granules, nor what the expected outcome of such an effect would "further test the model" set up by the HD and NaCl experiments. The average reader needs more hand-holding throughout this entire section (related to figure 2) to follow the exposition of the results.

4D) Continuing the comment above: The average reader also needs a better explanation of what hypothesis is being tested in Figure 1 with the pharmacological inhibition of calcium.

5) It is unclear why Bcd translation could not be measured in the endos mutant background, but it would be necessary to measure Bcd translation in the endos background. If genotypically it is not possible/inconvenient to invoke the suntag reporter in the endos background, would it not be sufficient to immunostain against Bcd itself? Different Bcd antisera have recently been reported and distributed by the Wieschaus and the Zeitlinger groups.

6) Figure 4 overall is glorious, but there is a problem with panel C. What are the white lines? Why does the intensity for the green and magenta channel change abruptly in the middle of the embryo?

7) It is noted that neither the methods section or the supplement does not contain any mention of how the modeling was performed. How was parameter beta fit? At least a brief section should be added to the methods describing how beta was fit (pending adjustments suggested in comment 1 above). A platinum-level addition would include a modeling supplement that reports the sensitivity of model outcomes to changes in parameters.

Minor Comments:

- Line 28: "Source-Diffusion-Degradation" should be changed to "Synthesis-..."
- Line 39: "blastocyst" should be "blastoderm stage embryo".
- Line 81: "P bodies are an evolutionarily cytoplasmic RNP granule." is "conserved" missing here?
- Throughout the manuscript, there should be better reporting of the imaged genotypes and whether the suntag is being visualized by indirect immunostaining of fixed tissues or through an encoded nanobody-GFP fusion.
- Figure 1G: Why is the background staining so different across conditions? Is this a normalization artifact?
- Figure 2 legend: what is +Sch in the x-axis labels of figure 2B? The legend says that 2B is the quantification of the data in 2A, but there is no (presumed control) +Sch image in 2A.
- Figure 5A largely repeats information presented in figure 4A. Please consider moving to a supplement. Also, please re-orient embryos to follow the convention that dorsal-most surfaces be presented on the top of the displayed images.
- The lower-case roman numerals referred to in the text for figure 7B are not included in the corresponding figure panel.
- Figure 7C y-axis typo (concentration).
- Line 222: "make a long-range functional gradient": more accurate to say, "but also marks mature, Bcd protein which resolves in the expected long-range gradient."
- Methods: Please check that all buffers referred to as acronyms are both compositionally defined in the reagents table, and that full names are written out at the time of first mention in the presented order. For instance, Schneider's media is referred to a few times before defining the acronym about midway through the methods section.

Referees cross-commenting

OK, We've been asked to comment on each others' reviews. I am reviewer 3. We have not been asked, as far as I can tell, to come up with a consensus review.

Overall, I feel that we are all generally enthusiastic about this manuscript. From most to least enthusiastic, we have reviewer 1, 3, and finally 2. But all three of us are apparently advocating positively and encouraging revision and clarification because, as we all agree, these results are important to publish.

Consensus Strengths:

1. The experimental approach is elegant, rigorous, and innovative, especially the real-time visualization of Bcd translation.
2. The data provide new mechanistic insight into when and where bcd is translated and how this changes over developmental time.
3. The relocalization of bcd mRNAs to P bodies during nc14 and the implications for RNA degradation are particularly compelling.
4. The manuscript establishes a path toward refining reaction-diffusion models of morphogen gradients using direct measurements of translation dynamics.

I agree with all of Reviewer 1's minor points.

I agree with Reviewer 2's points about:

- Showing the SunTag validation data using the fluorescent reporter.
- Clarifying the noted "translation" vs. "protein" issues. This bothered me too, but I wasn't able to articulate the issue as well as done here. This major issue summarizes several of the Reviewer's comments.
- Generally tightening the precision with which the results are discussed.

Overall: we have all provided favorable reviews that require mostly tightening of the text, showing some control datasets, maybe quantifying more points across the AP axis, and presenting the SDD model more comprehensively (comparing with old/translation-agnostic model, reporting characteristic decay lengths at different nuclear cycles, incorporating the reported change in translation rate across nuclear cycles (if this survives the clarification of what 'translation' means per Reviewer 2's comments), and perhaps providing more methodological detail on how parameters were fit).

2. Significance:

Significance (Required)

The importance of this study is at several levels. For the developmental biologist, it addresses important mechanisms of translational control and RNA stability over the functional lifetime of a single, critical biological cue that governs embryonic patterning. Not only do the experiments provide quantification of these features, but also point to likely candidates (P-bodies) for gating bcd's translation in the narrow window between egg activation and cellular blastoderm. For the biophysically-inclined, this adds critical quantitative information of translational state that allows for further refining computational models for how this manifestation of a reaction-diffusion

system actually comes together in a complex biological context.

The primary audience for this work will be the two groups above: developmental biologists and scientists interested in the quantitative modeling of biological phenomena.

3. How much time do you estimate the authors will need to complete the suggested revisions:

Estimated time to Complete Revisions (Required)

(Decision Recommendation)

Between 1 and 3 months

4. *Review Commons* values the work of reviewers and encourages them to get credit for their work. Select 'Yes' below to register your reviewing activity at Web of Science Reviewer Recognition Service (formerly Publons); note that the content of your review will not be visible on Web of Science.

Web of Science Reviewer Recognition

Yes

Reviewer #1

Evidence, reproducibility and clarity

In this paper the authors use the Suntag system to visualise bcd mRNA translation in the Drosophila embryo. They elucidate the relationship between bcd mRNA translation and P body localisation. In the oocyte, bcd mRNAs are localised in P bodies and translationally repressed, but upon egg activation bcd mRNAs are released from P bodies and translated. In addition, during mid-nc14, bcd mRNAs become localised to embryonic P bodies and degraded. The authors use their data to modify the Synthesis, Diffusion, Degradation model of Bcd gradient formation, which recapitulates the Bcd gradient detected experimentally.

Overall, I think the data are of high quality and support the authors' conclusions.

We thank the Reviewer for their positive assessment and the helpful suggestions below.

I only have minor comments, as follows:

- Fig 1B - add arrows showing mRNAs being translated or not (the latter mentioned in line 113 is not so easy to see).

We have magnified the inset of the colocalisation in the right column; we added arrows and arrowheads to differentiate colocalised and non-colocalised *bcd* with translating SunTag.

This is the edited part of Figure 1:

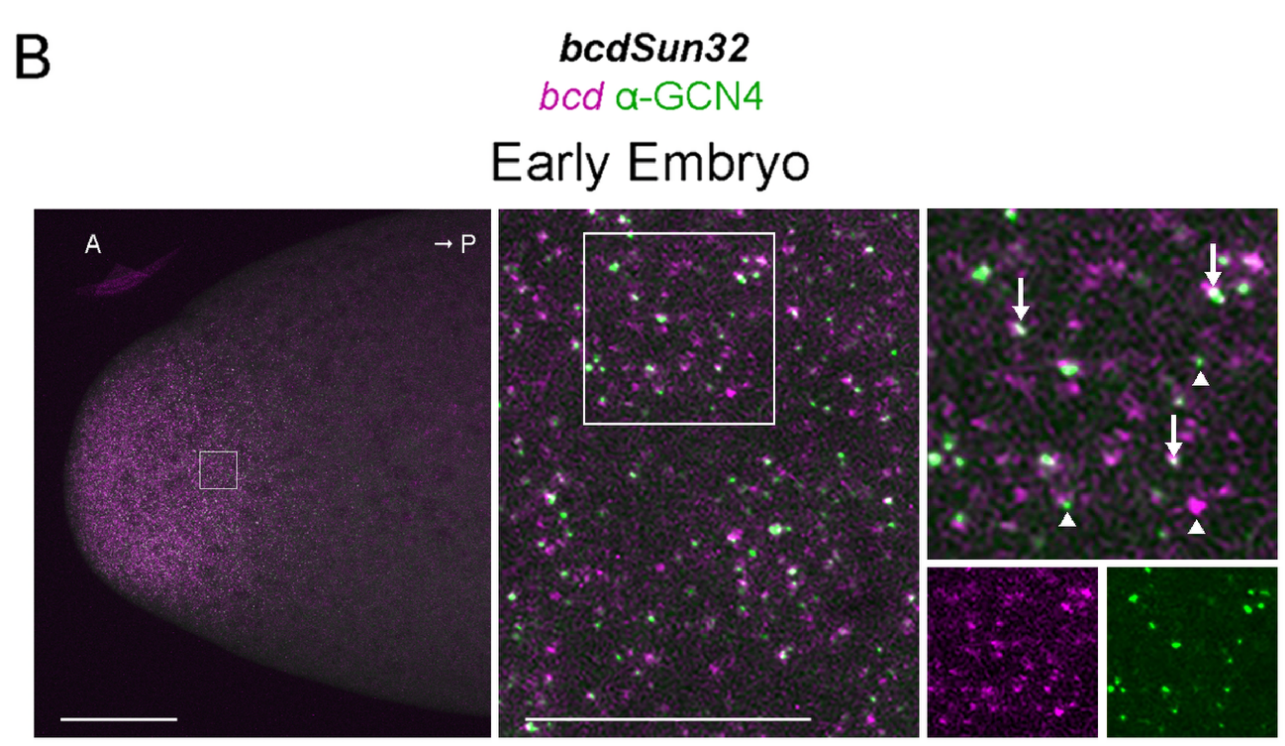

- Fig 2A - add a sentence explaining why 1,6HD, 2,5HD and NaCl disrupt P bodies.

We have added the information on the use of 1,6HD, 2,5HD, and NaCl to disrupt P-bodies as below.

Revised line 158: "To further show that *bcd* storage in P bodies is required for translational repression, we treated mature eggs with chemicals known to disrupt RNP granule integrity (31, 37, 69-72). Previous work has shown that the physical properties of P bodies in mature *Drosophila* oocytes can be shifted from an arrested to a more liquid-like state by addition of the aliphatic alcohol hexanediol (HD) (Sankaranarayanan et al., 2021, Ribbeck and Görlich, 2002; Kroschwald et al., 2017). While 1,6 HD has been widely used to probe the physical state

of phase-separated condensates both *in vivo* and *in vitro* (Alberti et al., 2019; McSwiggen et al., 2019; Gao et al., 2022), in some cells it appears to have unwanted cellular consequences (Ulianov et al., 2021). These include a potentially lethal cellular consequences that may indirectly affect the ability of condensates to form (Kroschwald et al., 2017) and wider cellular implications thought to alter the activity of kinases (Düster et al., 2021). While we did not observe any noticeable cellular issues in mature *Drosophila* oocytes with 1,6 HD, we also used 2,5 HD, known to be less problematic in most tissues (Ulianov et al., 2021) and the monovalent salt sodium chloride (NaCl), which changes electrostatic interactions (Sankaranarayanan et al., 2021)."

- Fig 4C - explain in the legend what the white lines drawn over the image represent. And why is there such an obvious distinction in the staining where suddenly the DAPI is much more evident (is the image from tile scans)?

Figure 4C is the tile scan image of a n.c.10 embryo and the white line classified the image into four quadrants. We used this image to quantify the extent of *bcd* (magenta) colocalisation to SunTag (green) in the anterior and posterior domains of the embryo in the bar graph shown in panel C'. There is a formatting error in the image. We will correct this in the revised version. We will also include the details of white lines in the legends. Finally, based on further reviewer comments, in the revised version this data is shifted to the supplementary information.

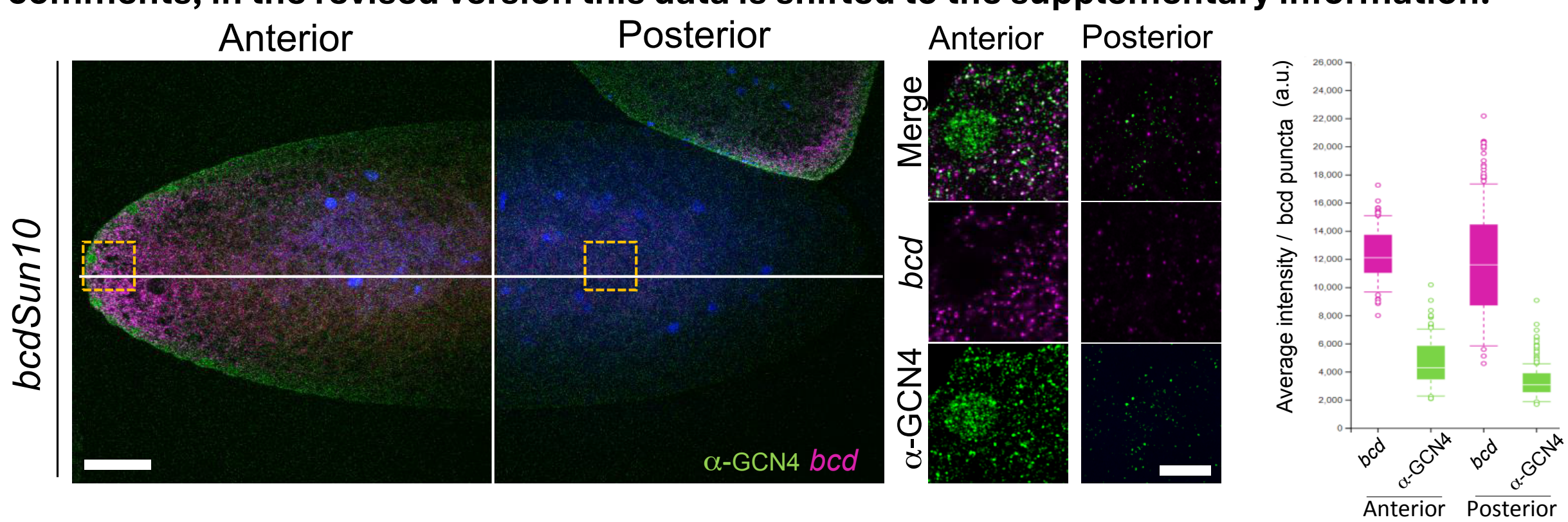

- Line 215 - 'We did not see any significant differences in the translation of bcd based on their position, however, there appears an enhanced translation of bcd localised basally to the nuclei (Figure S5).' Since the difference is not significant, I do not think the authors should conclude that translation is enhanced basally.

We agree with the reviewer. In this preliminary revision we have changed this statement to: "We did not see any differences in the translation of *bcd* based on their position with respect to the nuclei position (Figure S5)" (revised line 238-239).

- Line 218: 'The interphase nuclei and their subsequent mitotic divisions appeared to displace bcd towards the apical surface (Figure S6B).' Greater explanation is needed in the legend to Fig S6B to support this statement as the data just seem to show a nuclear division - I would have thought an apical-basal view is needed to conclude this.

We have rearranged this figure and shown in clarity the apical-basal view of the blastoderm nuclei and the displacement of *bcd* from the surface of the blastoderm in Figure S8 as shown below.

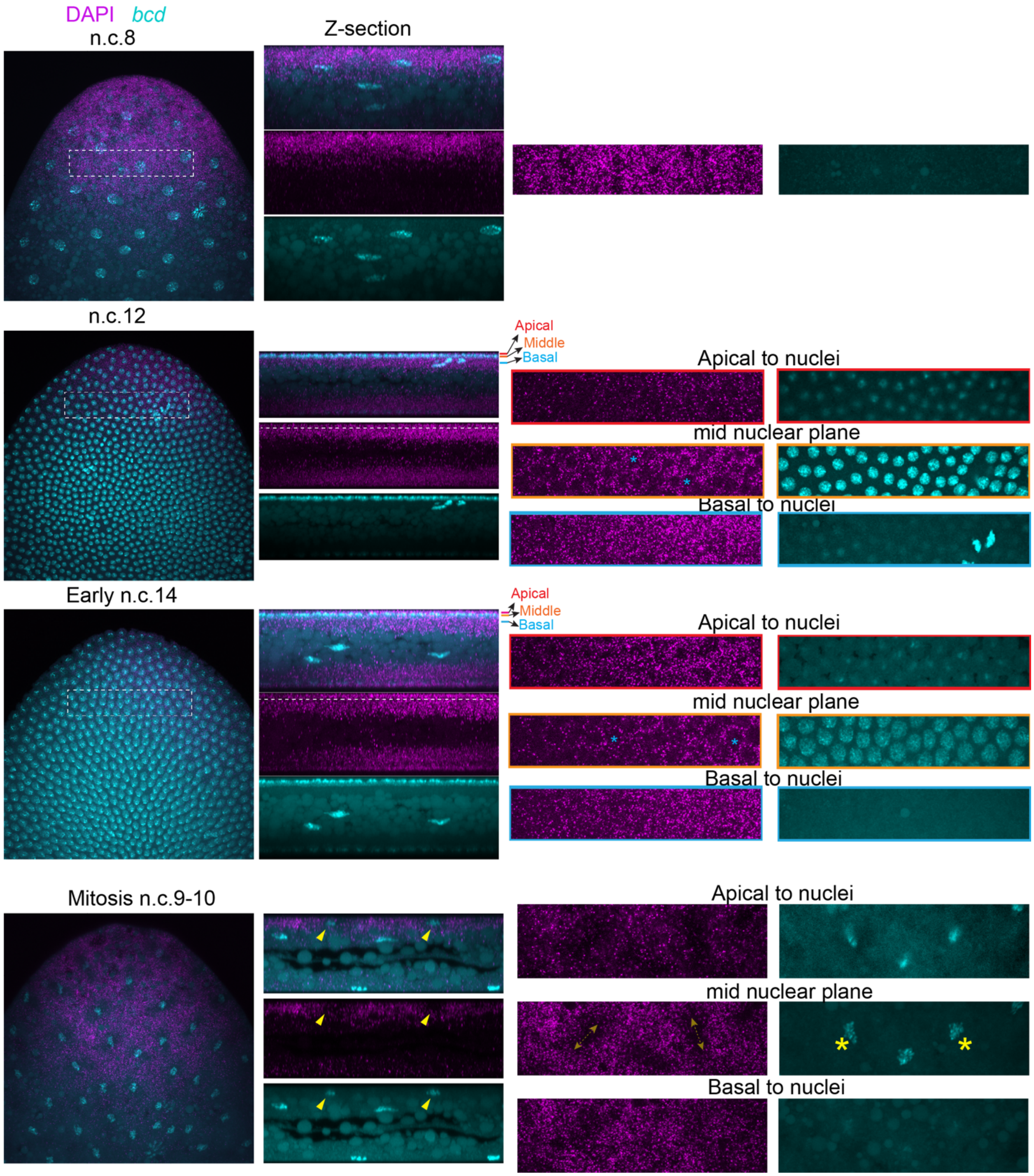

New Figure S8: n.c.8 - pre-cortical migration; n.c.12,14- post cortical migration; Mitosis stages of n.c.9-10. The cortical interphase nuclei at n.c. 12,14 displaces *bcd*. The nuclear area (DAPI, cyan) does not show any *bcd* particles (magenta) indicated by blue stars. The mitotic nuclei

(yellow arrowheads, yellow stars) displace *bcd* along the plane of nuclear division (doubled headed yellow arrows).

- Fig 5B - the authors compare Bcd protein distribution across developmental time. However, in the early time points cytoplasmic Bcd is measured (presumably as it does not appear nuclear until nc8 onwards) and compare the distribution to nuclear Bcd intensities from nc9 onwards. Is most/all of the Bcd protein nuclear localised form nc9 to validate the nuclear quantitation? Does the distribution look the same if total Bcd protein is measured per volume rather than just the nuclear signal? Are the authors assuming a constant fast rate of nuclear import?

From n.c.8 onwards, the Bcd signal in interphase nuclei builds up, with the nuclear intensity becoming very high compared to cytoplasmic Bcd. However, we do see significant Bcd signal in the cytoplasm (*i.e.,* above background). In earlier work, gradients of the nuclear Bcd and nuclear-import mutant Bcd overlapped closely (Figure 1B, Grimm et al., 2010). This essentially suggests the nuclear Bcd gradient reflects the corresponding gradient of cytoplasmic Bcd. Further, the nuclear import of Bcd occurs rapidly after photobleaching (Gregor et al., 2007). Based on these observations, and our own measurements, prior to n.c. 9, the cytoplasmic gradient is likely a good approximation of the overall shape, whereas post n.c. 9 the Bcd signal is largely nuclear localised. Further, the overall profile is not dependent on the nuclear volume.

- Line 259 - 'We then asked if considering the spatiotemporal pattern of bcd translation' - the authors should clarify what new information was included in the model. Similarly in line 286, 'By including more realistic bcd mRNA translation' - what does this actually mean? In line 346, 'We see that the original SDD model .... was too simple.' It would be nice to compare the outputs from the original vs modified SDD models to support the statement that the original model was too simple.

We will improve the linking of the results to the model. The important point is that when and where Bcd production occurs is more faithfully used, compared with previous approximations. By including more realistic production domains, we can replicate the observed Bcd gradient within the SDD paradigm without resorting to more complex models.

- Fig S1A - clarify what the difference is between the 2 +HD panels shown.

The two +HD panels at stage 14 indicate that upon the addition of HD, there are no particles in 70% of the embryos, and 30% show reduced particles. We will add this information to the figure legend.

- Fig S2E - the graph axis label/legend says it is intensity/molecule. Since intensity/molecule is higher in the anterior for bcd RNAs, is this because there are clumps of mRNAs (in which case it's actually intensity/puncta)?

The density of mRNA is very high in the anterior pole; there is a chance that more than one *bcd* particle is within the imaged puncta (due to optical resolution limitations). We will change the y-axis to average intensity per molecule to average intensity per puncta.

- Fig S4 - I think this line is included in error: '(B) The line plots of bcd spread on the Dorsal vs. Ventral surfaces.'

Yes, we will correct this in the revision.

- In B, D, E - is the plot depth from the dorsal surface? I would have preferred to see actual mRNA numbers rather than normalised mRNAs. In Fig S4D moderate, from 10um onwards there are virtually no mRNA counts based on the normalised value, but what is the actual number? The equivalent % translated data in Fig S4E look noisy so I wonder if this is due to there being a tiny mRNA number. The same is true for Figs S4D, E 10um+ in the low region.

Beyond 10um from the dorsal surface, the number of bcdsun10 counts is very low. It becomes negligible at the moderate and low domains. We will attach the actual counts of mRNA in all these domains as a supplementary table in the revised version.

Significance
General assessment
Strengths are: 1) the data are of high quality; 2) the study advances the field by directly visualising Bcd mRNA translation during early Drosophila development; 3) the data showing re-localisation of bcd mRNAs to P bodies nc14 provides new mechanistic insight into its degradation; 4) a new SDD model for Bcd gradient formation is presented. Limitations of the study are: 1) there was already strong evidence (but no direct demonstration) that bcd mRNA translation was associated with release from P bodies at egg activation; 2) it is not totally clear to me how exactly the modified SDD model varies from the original one both in terms of parameters included and model output.

This is the first direct demonstration of the translation of *bcd* mRNA released as a single mRNA from P bodies. Previously, we have shown that P bodies disruption releases single *bcd* from the condensates (31). We have captured a comprehensive understanding of the status of individual *bcd* translation events, from their release from P bodies at the end of oocyte maturation until the end of blastoderm formation.

The underlying SDD model – that of localised production, diffusion, and degradation – is still the same (up to spatially varying diffusion). Yet the model as originally formulated did not fit all aspects of the data, especially with regards to the system dynamics. Here, we demonstrate that by including more accurate approximations of when and where Bcd is produced, we can explain the formation of the Bcd morphogen gradient without recourse to any further mechanism.

Reviewer #2
Evidence, reproducibility and clarity

In this manuscript by Athilingam et al., the authors are studying the translation of the morphogen Bicoid (Bcd), which is in anterior-posterior patterning of the blastoderm Drosophila embryo. They have used an array of sunTag elements in the 5' UTR of Bcd to detect the localization of translation.

They found that, not only is Bcd not translated until egg activation, but it can only be translated at the anterior pole, even though bcd mRNA has a broader spatial distribution.

In general, the paper uses a cutting-edge methodology to address one of the foundational questions of the best-studied morphogen gradient: namely, what is the spatial distribution of the Bcd source? Together with the dynamics of its spreading (which they addressed in a separate study in 2024) and Bcd degradation, their results point to a modified form of the synthesis/diffusion/degradation (SDD) model of Bcd gradient formation, which they have analyzed in the final subsection of the results. However, there are several major issues that erode the validity and impact of the paper, most of which can be put into the category of vague explanations, missing information, or contradictory statements, making it hard to understand/verify what conclusions can be drawn. This is also coupled with vague figures and captions. We describe these, and a few minor issues, in detail below:

We thank the Reviewer for their detailed assessment and recognition of the paper achievements. We agree that the linking of results can be improved and we address these comments below.

- Line 114: The authors claim to have validated the SunTag using a fluorescent reporter, but do not show any data. Ref 60 is a general reference to the SunTag, and not the Bcd results in this paper. Perhaps place their data into a supplemental figure or movie?

To show the validation of our *bcdSun32* line, we have composed a new Figure S1 that shows the translating *bcdSun32* (magenta) colocalising to the ScFV-mSGFP2 (green). Yellow arrowheads in the zoom (right panel) points to the translating *bcdSun32* (magenta) and red arrowheads points to the untranslated *bcdSun32*. In addition, we have also shown the validation of *bcdSun32* with the anti-GCN4 staining in the main Figure 1B.

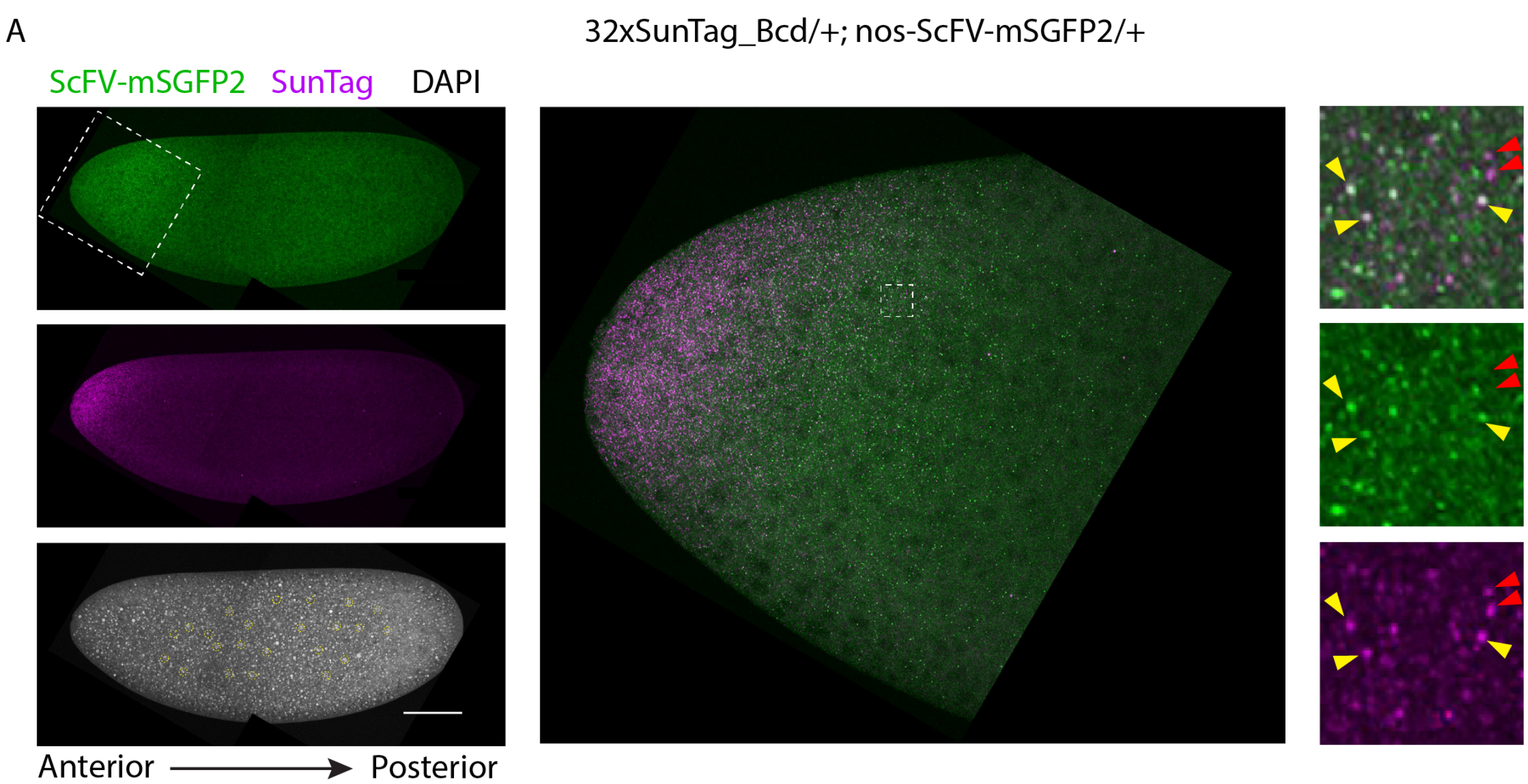

Further, we have dedicated supplementary Figure S3 (previously Figure S2) for the validation of our *bcdSun10* construct. Briefly, *bcdSun10* is inserted into att40 site of chr.2. We did a rescue experiment, where *bcdSun10* rescued the lethality of homozygous *bcdE1* null mutant. We then performed a colocalisation experiment using smFISH, where we demonstrated that almost all *bcd* in the anterior pole are of type *bcdSun10*. We targeted specific fluorescent FISH probes against 10xSunTag sequence (magenta, Figure S2A) and bcd coding sequence (magenta, Figure S2A). Upon colocalisation, we found ~90% of the mRNA are of *bcdSun10* type. The remaining 10% could likely be contributed by the noise level (Figure S2B). We will make sure these points are clear in the revised manuscript.

- Line 128 and Fig. 1E: The claim that bcd becomes dispersed is difficult to verify by looking at the image. The language could also be more precise. What does it mean to lose tight association? Perhaps the authors could quantify the distribution, and summarize it by a length scale parameter? This is one of the main claims of the paper (cf. Line 23 of the abstract) but it is described vaguely and tersely here.

We have changed the text from, "We also confirmed that *bcd* becomes dispersed, losing its tight association with the anterior cortex (Figure 1E) (31)" to, "We also confirmed that *bcd* is released from the anterior cortex at egg activation (Figure 1E) (31, 21)." (Revised line 131).

The release of *bcd* mRNA at egg activation was first shown in 2008 (Ref 21, Figure 4, D-E) and again in 2021 (Ref 31, Figure 7 B and E). The main point in line 127-128, "P bodies disassembled and *bcd* was no longer colocalised with P bodies" and the novel aspect of line 23 is "translation observed". The distribution of *bcd* mRNA after egg activation was not the point of this section. We have improved the writing in the revision to make this clearer.

- Line 146, Fig. 1G: This is a really important figure in the paper, but it is confusing because it seems the authors use the word "translation," when they mean "presence of Bcd protein." In other places in the paper, the authors give the impression that "bcd translation" means translation in progress (assayed by the colocalization of GCN4 and bcd mRNA). However, in Fig. 1G, the focus is only on GCN4. Detecting Bcd protein only at the anterior does not mean that translation happens only at the anterior (e.g., diffusion or spatially-restricted degradation could be in play).

In Figure 1G, we have shown only the "translated" Bcd by staining with a-GCN4. We have changed line 146 from, "Consistent with previous findings, we only observed *bcd* translation at the anterior of the activated egg and early embryo (Figure 1G-H) (3, 68)" to, "Consistent with previous findings, we only observed the presence of Bcd protein at the anterior of the activated egg and early embryo (Figure 1G-H) (3, 68). (Revised line 151-153). We will use "translating *bcd*" or "*bcd* in translation" where we show colocalisation of *bcd* with BcdSun10 or BcdSun32 elsewhere in the manuscript.

We did not mean to claim that translation occurred only in the anterior pole. We show that the abundance of *bcd* is very high in the anterior pole (in agreement with previous work) and that this is where the majority of observed translation events took place. Indeed, we have also shown that posteriorly localised mRNAs have the same BcdSun10 intensity per *bcd* puncta from the posterior pole (Figure 3B & 4C' and Figure S2 E), but these are much fewer in number.

- It would also be helpful to show a plot with quantification of Bcd detection (or translation) on the

y-axis and a continuous AP coordinate on the x-axis, instead of just two points (anterior and posterior poles, the latter of which is uninteresting because observing no Bcd at the posterior pole is expected).

In Figure 1G,H, our aim was to test whether release from P bodies allowed for *bcd* mRNA to be translated. We used the presence of Bcd protein at the anterior domain of the oocytes to show this. The posterior pole was included as an internal control. To show the spatial distribution of *bcd* mRNA and its translation, we used early blastoderm (Figure 3, Figure S4).

- Another issue with Fig. 1G is that the A and P panels presumably have different brightness and contrast. If not, just from looking at the A and P panels, the conclusion would be that Bcd protein is diffuse (and abundant) in the posterior and concentrated into puncta in the anterior. The authors should either make the brightness and contrast consistent or state that the P panel had a much higher brightness than the A panel.

We agree with this shortcoming. We have now added the following to Figure 1 legend to clarify this observation. "G: Representative fixed 10 µm Z-stack images (from 10 samples) showing BcdSun32 protein (anti-GCN4) is only present at the anterior of an *in vitro* activated egg or early embryo 30-minute post fertilization. BcdSun32 protein is not detected in these samples at the posterior pole (image contrast increased to highlight the lack of distinct particles at the posterior). BcdSun32 protein is also not detected at the anterior or posterior of a mature oocyte or an *in vitro* activated egg incubated with NS8953 (images have the contrast increased to highlight the lack of distinct particles). Scale bar: 20 mm; zoom 2 mm." (Revised line 623).

- Line 176: This section is very confusing, because at this point the authors already addressed the spatial localization of translation in Fig. 1G,H (see my above comment). However, here it seems the authors have switched the definition of translation back to "translation in progress." Therefore, the confusion here could be eliminated by addressing the above point.

In the revised version, we will use Bcd protein when shown with anti-GCN4 staining. We will use "translating *bcd*" or "*bcd* in translation" where we show colocalisation of *bcd* with a-GCN4 (BcdSun10 or BcdSun32). We will change this in the corresponding text.

- Line 185: The sentence here is seemingly contradictory: "most...within 100 microns" implies that at least some are beyond 100 microns, while the sentence ends with "[none]...more than 100 microns." The language could perhaps be altered to be less vague/contradictory.

We will clarify this in the revised version. There are few particles visible beyond 100 um. In the lower panel of Figure 3B, the posterior domain shows few particles. However, their actual number compared to *bcd* counts within the 100 um is negligible (Figure3C). Nonetheless, the few *bcd* particles we observe do seem to be under translation (quantified in Figure 4C' and Figure S2E).

- Line 204: It would be really nice to have quantification of the translation events, such as curves of rate of translation as a function of a continuous AP coordinate, and a curve for each nc.

In the revised version we will provide the results quantifying the translation events across the anterior- posterior axis. This will provide a clarity to the presence of *bcd* and their translation in the posterior domain with time.

Our colocalisation analysis is semi-automated. It includes an automated counting of the individual *bcd* particle counts and a manual judgement of the colocalised BcdSun10 protein (distinct spots, above noise) to *bcd* particles (Figure S3D). The *bcd* particle counts ran into thousands in each cyan square box (measuring 50um radius and ~ 20um deep from the dorsal surface). We selected three such boxes covering 150um (continuously) from the anterior pole across A-P axis and 20um deep of the flattened embryo mounts across D-V axis (Figure 3A-C, Figure S4). We have also scanned scarce particles in the posterior; however, *bcd* counts are very low compared to the anterior. Further, in Figure 4 we have repeated the same technique to measure translation of *bcd* particles in embryos at different nuclear cycles.

We have also shown continuous intensity measurements of *bcd* particles with their respective BcdSun10 gradient in Figure 5 across the A-P axis at different nuclear cycles. Here, we know BcdSun10 intensity is not only from the "translating" *bcd* (colocalised BcdSun10 to *bcd* particles) but also from the translated BcdSun10 freely diffusing (non-colocalised BcdSun10 to *bcd* particles). As asked by the reviewer, in the revised version we will add *bcd* counts and their translation status from anterior to posterior axis for each of the nuclear cycles.

In our future work, we planned to generate MS2 tagged *bcdSun10* to measure the rates of translation in live across all nuclear cycles.

- Line 209 and Fig 4C: The authors use the terms "intensity of translation events" or "translation intensity" without clearly defining them. From the figure (specifically from the y-axis label), it looks like the authors are quantifying the intensity per molecule (which is not clearly the same thing as "translation intensity"), but it would be nice if that were stated explicitly.

In the relevant result section, we have changed the results text to "the intensity of translation events" for explaining the results of Figure 4C'.

- In addition, the authors again quantify only two points. This is a continuously frustrating part of the manuscript, which applies to nearly all figures where the authors looked only at two points in space. At a typical sample size of N = 3, it seems well within time constraints to image at multiple points along the AP axis.

In addition to the quantification shown at the anterior and posterior locations of the embryo in the Figure 3 and 4, we will show in the revised version, the quantification of translation events across all locations from the anterior to the posterior. We will use three embryos for each nuclear cycle from n.c.1 to 14.

- Furthermore, it sounds like the authors are saying the "translation intensity" is the same in anterior and the posterior, which is counterintuitive. The expectation is that translation would be undetectable at the posterior end, in part because bcd mRNA would not be present. (Note that this expectation is even acknowledged by the authors on Line 185, which I comment on above, and also on Line 197). There should also be very low levels of Bcd protein (possibly undetectable) at the posterior pole. As such, the authors should explain how they think their claim of the same

"translation intensities" in the anterior vs posterior fits into the bigger picture of what we know about Bcd and what they have already stated in the manuscript. They should also explain how they observed enough molecules to quantify at the posterior end. The authors should also disclose how many points are in each box in the boxplot. For example, the sample size is N = 3 embryos. In just three embryos, how many bcd/GCN4 colocalizations did the authors observe at the posterior end of the embryo?

In n.c.4 in Figure3, we saw few *bcd* particles in the posterior. However, at n.c.10 in Figure 4C' the number of posterior *bcd* particles are higher than at the early stages. We have quantified them in Figure 4C'. We will clarify this from the new set of quantification we are undertaking now to quantify translation across the A-P axis in the revision.

Finally, we will also provide the number of *bcd* particle counts and their colocalisation with a-GCN4 as a supplementary table.

- Line 215: The sentence that starts on this line seems self-contradictory: I cannot tell whether or not there is a difference in translation based on position.

We have not observed any difference in the translation of *bcd* particles depending on the position along the Z-axis. We will edit this in our revised version.

- Line 229: Long-ranged is a relative term. From the graph, one could state there is some spatial extent to the mRNA gradient, so it is unclear what the authors mean when they say it is not "long-ranged." Could the mRNA gradient be quantified, such as with a spatial length scale? This would provide more information for readers to make their own conclusions about whether it is long-ranged.

We have quantified the *bcd* mRNA gradient for each n.c. (Figure 5B-C); absolute *bcd* intensities in Figure 5B, left panel and the normalised intensities in Figure 5C. The length of the mRNA spread appears constant with the half-length maximum of ~75um across all nuclear cycles. Our conclusion of a long ranged Bcd gradient is based on the comparisons of the half-length maximum measurements of *bcd* particles and BcdSun10 (Figure 5D).

- Line 230: When the authors claim the Bcd gradient is steeper earlier, a quantification of the spatial extent (exponential decay length scale) would be appropriate. Indeed, lambda as a function of time would be beneficial. It should also be placed in context of earlier papers that claim the spatial length scale is constant.

We will show this effectively from the live movies of *bcdSun10*/nanos-scFv-sGFP2 in the revised version.

- Lines 235-236: The two sentences that start on these two lines are vague and seemingly contradictory. The first sentence says there is a spatial shift, but the second sentence sounds like it is saying there is no spatial change. The language could be more precise to explain the conclusions.

We agree with the reviewer. We will edit this in revision.

Minor issues/typos (still must be addressed for content):

- Line 81: Probably meant "evolutionarily conserved"

Yes, we have changed, "P bodies are an evolutionarily cytoplasmic RNP granule" to, "P bodies are an evolutionarily conserved cytoplasmic RNP granule."(Revised line 84-85).

- Figure 1 legend: part B says "from 15 samples" but also says N = 20. Which is it, or do these numbers refer to different things?

We have edited this from, "early embryo (from 15 samples)" to, "early embryo (from 20 samples)". (Revised line 602).

- Line 217: migration of what?

Edited to "cortical nuclear migration".

- Line 228: "early embryo" is vague. The authors should give specific time points or nuclear cycle numbers.

Edited to "nuclear cycles 1-8".

- Line 301: Other locations in the paper say 75 microns or 100 microns.

We will make the changes. It is 100 um.

- Fig. 5: all images should be oriented such that the dorsal midline is on the upper half of the embryo/image.

We will flip the image to match.

- Fig. 5B: There are light tan and/or light orange curves (behind the bold curves) that are not explained.

It is the standard deviation. This will be explained.

- Fig. 5C: the plot says "normalized" but nowhere do the authors describe what the curves are normalized to. There is also no explanation for what the broad areas of light color correspond to.

Normalised to the *bcd* intensity maxima. This will be explained.

Significance
The results, if upheld, are highly significant, as they are foundational measurements addressing a longstanding question of how morphogen gradients are formed, using Bcd (the foundational morphogen gradient) as a model. They also address fundamental questions in genetics and molecular biology: namely, control of mRNA distribution and translation.

We thank reviewer 2 for highlighting the importance of our work in the field. We are confident that we address the issues raised by Reviewer 2 with the new set of quantifications we are currently working on.

Reviewer #3

Evidence, reproducibility and clarity

This is a review of "Dynamics of bicoid mRNA localization and translation dictate morphogen gradient formation" by Athilingam et al. In this manuscript, the authors perform quantification of mRNA localization and translation of bicoid, spanning oogenesis through the maternal to zygotic transition, yielding a definitive characterization of Bicoid gradient formation. The experiments, analysis, and interpretation are on the whole performed rigorously. I very much enjoyed this paper, partly for incorporating the aspects of bcd regulation during oogenesis, which compared to embryonic function of bcd is relatively under-studied. Also valuable is improving the characterization of how bcd expression is shut down at NC14. I have several major comments for revision, and a few minor comments. I should stress that none of the major comments are terrible but are intended to improve the impact/readability/flow of this nice manuscript. With the exception of a straightforward immunostaining experiment, all major comments constitute reworking of the model or the text.

Major Comments:

1) It is not evident from the main results and methods text that the new SDD model incorporates the phenomenon reported in figure 4B. From my reading, the parameter beta accounts for the Bcd translation rate, which according to figure 7B(ii) effectively switches from off to on around fertilization and thereafter remains constant. Figure 4B shows that the fraction of bcd mRNA engaged in translation decreases beginning around NC12/13, and this is one of the more powerful results that comes from monitoring translation in addition to RNA localization/abundance/stability. My expectation based on figure 4B would be that parameter beta should decrease over time beginning around 90-100 minutes and approach zero by ~150 minutes. This rate could be fit to the experimental data that yields figure 4B. The modeling should be repeated while including this information.

This is a good observation. Currently, the reduced rate of *bcd* translation is modelled by incorporating an increased rate of *bcd* mRNA degradation. Of course, this could also be reduced by a change in the rate of translation directly. As stated already, the beta parameter is the least well characterised. In the revision, we will include a model where beta changes but not the mRNA degradation rate. We will improve the discussion to make this point clearer.

2) The presentation of the SDD model should be expanded to address how well the characteristic decay length fits A) measured Bcd protein distributions, B) measured at different nuclear cycles. This would strengthen the claim that the new SDD model better captures gradient dynamics given the addition of translation and RNA distribution. These experimental data already exist as reported in Figure 5. In the current Figure 7, panels D and D' add little to the story and could be moved to a supplement if the authors want to include it (in any case, please fix the typo on the time axis of fig

7D' to read "hours"). The model per cell cycle and the comparison of experimental and modeled decay lengths could replace current D and D'.

Originally, we kept discussion of the SDD model only to core points. It is clear from all Reviewers that expanding this discussion is important. In the revision, we will refocus Figure 7 on describing new results that we can learn. As outlined in the responses above, this paper reveals an important insight: the SDD model – with suitable modifications such as temporally restricted Bcd production – can explain all observed properties of Bcd gradient formation. Other mechanisms – such as *bcd* mRNA gradients – are not required.

3) The exposition of the manuscript would benefit significantly by including a section either in the introduction or the appropriate section of the results that defines the competing models for gradient formation. In the current version, these models are only cited, and the key details only come out late (e.g., lines 302 onward, in the Discussion). Nevertheless, some of the results are presented as if in dialog with these models, but it reads as a one-sided conversation. For instance: Figure 3. The undercurrent in this figure is the RNA-gradient model. In the context of this model, the results clearly show that translation of bcd is restricted to the anterior. Without this context, Figure 3 could read as a fairly unremarkable observation that translation occurs wherever there is mRNA. Restructuring the manuscript to explicitly name competing models and to address how experimental results support or detract from each competing model would greatly enhance the impact of the exposition.

We thank the reviewer for this suggestion. We will add the current models of Bcd gradient formation in the introduction section and will change the narrative of results in the section explaining the models.

4A) Related to point 3: The entire results text surrounding Figure 2 should be revised to include more detail about A) what specific hypotheses are being tested; and B) to critically evaluate the limitations of the experimental approaches used to evaluate these hypotheses. Hexanediol and high salt conditions are not named explicitly in the text, but the text touts these as "chemicals" that "disrupt P-body integrity." This implies that the treatments are specific to P-bodies. Neither of these approaches are only disrupting P Body integrity. This does not invalidate this approach, but the manuscript needs to state what hypothesis HD and NaCl treatment addresses, and acknowledge the caveats of the approach (such as the non-specificity and the assumptions about the mechanism of action for HD).

We have made the following edits to resolve this point. Revised line 158: "To further show that *bcd* storage in P bodies is required for translational repression, we treated mature eggs with chemicals known to disrupt RNP granule integrity (31, 37, 69-72). Previous work has shown that the physical properties of P bodies in mature *Drosophila* oocytes can be shifted from an arrested to a more liquid-like state by addition of the aliphatic alcohol hexanediol (HD) (Sankaranarayanan et al., 2021, Ribbeck and Görlich, 2002; Kroschwald et al., 2017). While 1,6 HD has been widely used to probe the physical state of phase-separated condensates both *in vivo* and *in vitro* (Alberti et al., 2019; McSwiggen et al., 2019; Gao et al., 2022), in some cells it appears to have unwanted cellular consequences (Ulianov et al., 2021). These include a potentially lethal cellular consequences that may indirectly affect the ability of condensates to form (Kroschwald et al., 2017) and wider cellular implications thought to alter the activity of kinases (Düster et al., 2021). While we did not observe any noticeable cellular issues in mature

***Drosophila* oocytes with 1,6 HD, we also used 2,5 HD, known to be less problematic in most tissues (Ulianov et al., 2021) and the monovalent salt sodium chloride (NaCl), which changes electrostatic interactions (Sankaranarayanan et al., 2021)."**

4B) Continuing the comment above: it is good that the authors checked that HD and NaCl treatment does not cause egg activation. But no one outside of the field of Drosophila egg activation knows what the 2-minute bleach test is and shouldn't have to delve into the literature to understand this sentence. Please explain in one sentence that "if eggs are activated, then x happens following a short exposure to bleach (citations). We exposed HD and NaCl treated eggs to bleach and observed… ."

We have made the following edits to resolve this point. Revised line 174: "After treating mature eggs with these solutions, we observed BcdSun32 protein in the oocyte anterior (Figure 2A-B). One caveat to this experiment could be that treating mature eggs with these chemicals results in egg activation which would in turn generate Bcd protein. To eliminate this possibility, we first screened for phenotypic egg activation markers, including swelling and a change in the chorion (73). We also applied the classic approach of bleaching eggs for two minutes which causes lysis of unactivated eggs (74). All chemically treated eggs failed this bleaching test meaning they were not activated (74). While we unable to rule out non-specific actions of these treatments, these experiments corroborate that storage in P bodies that adopt an arrested physical state is crucial to maintain *bcd* translational repression (31)."

4C) Continuing the comment above: The section of the results related to the endos mutation needs additional information. It is not apparent to the average reader how the endos mutation results in changes in RNP granules, nor what the expected outcome of such an effect would "further test the model" set up by the HD and NaCl experiments. The average reader needs more hand-holding throughout this entire section (related to figure 2) to follow the exposition of the results.

We have made the following edits to resolve this point. Edited line 185: "Finally, we used a genetic manipulation to change the physical state of P bodies in mature oocytes. Mutations in *Drosophila* Endosulfine (Endos), which is part of the conserved phosphoprotein α-endosulfine (ENSA) family (75), caused a liquid-like P body state after oocyte maturation, similar to that observed with chemical treatment (Figure 2C) (31). This temporal effect matched the known roles of Endos as the master regulator of oocyte maturation (75, 76). *endos* mutant oocytes lost the colocalisation of *bcd* mRNA and P bodies, concurrent with P bodies becoming less viscous during oocyte maturation (Figure 2D, Figure S1). Particle size and position analysis showed that *bcd* mRNA prematurely exhibits an embryo distribution in these mutants (Figure 2E). Due to genetic and antibody constraints, we are unable to test for translation of *bcd* in the *endos* mutant. However, it follows that *bcd* observed in this diffuse distribution outside of P bodies would be translationally active (Figure 2E-F)."

4D) Continuing the comment above: The average reader also needs a better explanation of what hypothesis is being tested in Figure 1 with the pharmacological inhibition of calcium.

We have made the following edits to resolve this point. Revised line 138: "We next sought to maintain the relationship between *bcd* mRNA and P bodies through egg activation. This would act as a control to further test if colocalisation of *bcd* to P bodies was necessary for its translational repression. Previous work has shown that a calcium wave is required at egg

activation for further development (references to add Kaneuchi et al., 2015; York-Anderson et al., 2019; Hu and Wolfner, 2019). Chemical treatment with NS8593 disrupts this calcium wave, while other phenotypic markers of egg activation are still observed (58). Using NS8593 to disrupt the calcium wave in the activated egg, we show P bodies are retained during *ex vivo* egg activation (Figure 1E). In these treated eggs, *bcd* mRNA remains colocalised with the retained P bodies (Figure 1F). Based on these results and previous observations (31, 66), we hypothesised that the loss of colocalisation between *bcd* and P bodies correlates with *bcd* translation."

5) It is unclear why Bcd translation could not be measured in the endos mutant background, but it would be necessary to measure Bcd translation in the endos background. If genotypically it is not possible/inconvenient to invoke the suntag reporter in the endos background, would it not be sufficient to immunostain against Bcd itself? Different Bcd antisera have recently been reported and distributed by the Wieschaus and the Zeitlinger groups.

We have recently received the Bcd antibody from the Zeitlinger group. This has not been shown to work for immunostaining. It remains unclear if it will be successful in this capacity, but we are currently testing it and will include this experiment in the revision if successful.

6) Figure 4 overall is glorious, but there is a problem with panel C. What are the white lines? Why does the intensity for the green and magenta channel change abruptly in the middle of the embryo?

These white lines divide the embryo into 4 compartments. We used this method to quantify the intensity of Bcd translation with respect to the *bcd* puncta. We will correct this image as there is a problem in formatting.

7) It is noted that neither the methods section or the supplement does not contain any mention of how the modeling was performed. How was parameter beta fit? At least a brief section should be added to the methods describing how beta was fit (pending adjustments suggested in comment 1 above). A platinum-level addition would include a modeling supplement that reports the sensitivity of model outcomes to changes in parameters.

We apologise for this omission and will include full methodological details in the revision.

Minor Comments:

- Line 28: "Source-Diffusion-Degradation" should be changed to "Synthesis-..."

We will edit in the revised version.

- Line 39: "blastocyst" should be "blastoderm stage embryo".

We will edit in the revised version.

- Line 81: "P bodies are an evolutionarily cytoplasmic RNP granule." is "conserved" missing here?

We will edit in the revised version.

- Throughout the manuscript, there should be better reporting of the imaged genotypes and whether the suntag is being visualized by indirect immunostaining of fixed tissues or through an encoded nanobody-GFP fusion.

We will explain in detail in the revised version.

- Figure 1G: Why is the background staining so different across conditions? Is this a normalization artifact?

We agree with this shortcoming. We have now added the following to the figure legend to clarify this observation. "G: Representative fixed 10 μm Z-stack images (from 10 samples) showing BcdSun32 protein (anti-GCN4) is only present at the anterior of an *in vitro* activated egg or early embryo 30-minute post fertilization. BcdSun32 protein is not detected in these samples at the posterior pole (image contrast increased to highlight the lack of distinct particles at the posterior). BcdSun32 protein is also not detected at the anterior or posterior of a mature oocyte or an *in vitro* activated egg incubated with NS8953 (images have the contrast increased to highlight the lack of distinct particles). Scale bar: 20 mm; zoom 2 mm." (Revised line 623).

- Figure 2 legend: what is +Sch in the x-axis labels of figure 2B? The legend says that 2B is the quantification of the data in 2A, but there is no (presumed control) +Sch image in 2A.

Thank you for this suggestion we have added the data to Figure 2A:

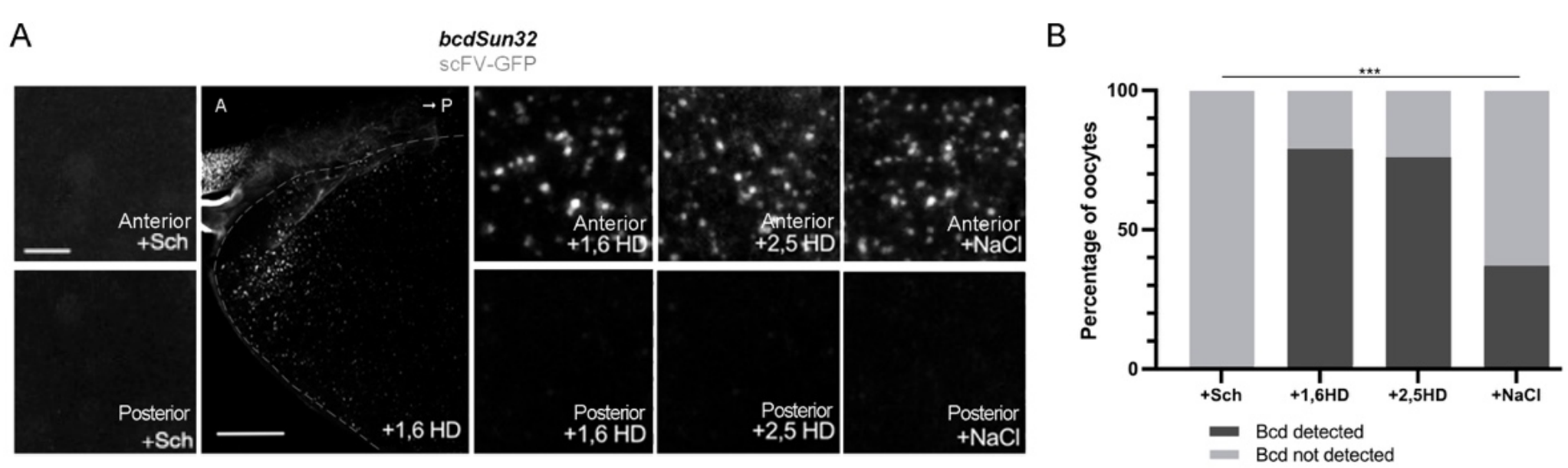

- Figure 5A largely repeats information presented in figure 4A. Please consider moving to a supplement. Also, please re-orient embryos to follow the convention that dorsal-most surfaces be presented on the top of the displayed images.

Thank you for this suggestion. We will consider moving Figure 5A to the supplementary.

- The lower-case roman numerals referred to in the text for figure 7B are not included in the corresponding figure panel.

We will edit in the revised version.

- Figure 7C y-axis typo (concentration).

We will edit in the revised version.

- Line 222: "make a long-range functional gradient": more accurate to say, "but also marks mature, Bcd protein which resolves in the expected long-range gradient."

We will edit in the revised version.

- Methods: Please check that all buffers referred to as acronyms are both compositionally defined in the reagents table, and that full names are written out at the time of first mention in the presented order. For instance, Schneider's media is referred to a few times before defining the acronym about midway through the methods section.

We have added to Figure 2B: "Quantification of experiments shown in A. The number of oocytes that displayed Bcd protein at the anterior as measured by the presence of BcdSun32 at the anterior of the oocyte, but not the posterior. Schneider's Insect Medium (+Sch) used as a negative control. N = 30 oocytes for each treatment. Scale bar: 5 um." (Revised line 646).

Significance

The importance of this study is at several levels. For the developmental biologist, it addresses important mechanisms of translational control and RNA stability over the functional lifetime of a single, critical biological cue that governs embryonic patterning. Not only do the experiments provide quantification of these features, but also point to likely candidates (P-bodies) for gating bcd's translation in the narrow window between egg activation and cellular blastoderm. For the biophysically-inclined, this adds critical quantitative information of translational state that allows for further refining computational models for how this manifestation of a reaction-diffusion system actually comes together in a complex biological context.

The primary audience for this work will be the two groups above: developmental biologists and scientists interested in the quantitative modeling of biological phenomena.

1st Editorial Decision **30th Jan 2026**

Dear Dr. Weil,

Thank you for submitting your manuscript for consideration by the EMBO Journal. I have now read your manuscript, the reviewer comments and your response to them. Based on our editorial assessment, the positive evaluations by the reviewers, and your revision outline, I would like to invite you to submit a revised version of the manuscript along the lines indicated in your revision plan.

We generally allow three months as standard revision time. Should you foresee a problem in meeting this three-month deadline, please let us know in advance in order to arrange an extension.

As a matter of policy, competing manuscripts published during this period will not negatively impact on our assessment of the conceptual advance presented by your study. However, please contact me as soon as possible upon publication of any related work to discuss the appropriate course of action.

When preparing your letter of response to the referees' comments, please bear in mind that this will form part of the Review Process File and will therefore be available online to the community. For more details on our Transparent Editorial Process, please visit our website: https://link.springer.com/partners/embo-press/editorial-policies#Peer%20review. Please also see the attached instructions for further guidelines on preparation of the revised manuscript.

Please feel free to contact me if you have any further questions regarding the revision. Thank you for the opportunity to consider your work for publication. I look forward to receiving your revised manuscript.

With kind regards,

Ieva Gailite

Ieva Gailite, PhD
Senior Scientific Editor
The EMBO Journal
Meyerhofstrasse 1
D-69117 Heidelberg
Tel: +4962218891309
i.gailite@embojournal.org

Instructions for preparing your revised manuscript:

Read our guidance for manuscript revisions and related editorial policies: https://link.springer.com/journal/44318/submission-guidelines#cms-Revised-submissions

Please make sure you upload a letter of response to the referees' comments together with the revised manuscript.

Please also check that the title and abstract of the manuscript are brief, yet explicit, even to non-specialists.

When assembling figures, please refer to our figure preparation guideline in order to ensure proper formatting and readability in print as well as on screen:
https://media.springernature.com/original/springer-cms/rest/v1/content/27825798/data/v1

At EMBO Press we ask authors to provide source data for the main manuscript figures. You will receive a separate email with instructions for providing source data with your revised manuscript, including how to upload and organize the files.

IMPORTANT: When you send the revision we will require
- a point-by-point response to the referees' comments, with a detailed description of the changes made (as a word file).
- a word file of the manuscript text.
- individual production quality figure files (one file per figure)
- a complete author checklist
- Expanded View files (replacing Supplementary Information)
- a Reagents and Tools Table as part of the Methods section

Please remember: Digital image enhancement is acceptable practice, as long as it accurately represents the original data and conforms to community standards. If a figure has been subjected to significant electronic manipulation, this must be noted in the figure legend or in the 'Materials and Methods' section. The editors reserve the right to request original versions of figures and the original images that were used to assemble the figure.

We realize that it is difficult to revise to a specific deadline. In the interest of protecting the conceptual advance provided by the work, we recommend a revision within 3 months (30th Apr 2026). Please discuss the revision progress ahead of this time with the editor if you require more time to complete the revisions. Use the link below to submit your revision:

Link Not Available

***

Rev_Com_number: RC-2025-03245
New_manu_number: EMBOJ-2026-123675-T
Corr_author: Weil
Title: Dynamics of bicoid mRNA localisation and translation dictate morphogen gradient formation

We thank the reviewers for their detailed and constructive comments. Below, we address each point in blue. Corresponding changes in the accompanying supplemental manuscript entitled "Revised Manuscript Markup" are highlighted in green.

Reviewer #1

Evidence, reproducibility and clarity

In this paper the authors use the Suntag system to visualise bcd mRNA translation in the Drosophila embryo. They elucidate the relationship between bcd mRNA translation and P body localisation. In the oocyte, bcd mRNAs are localised in P bodies and translationally repressed, but upon egg activation bcd mRNAs are released from P bodies and translated. In addition, during mid-nc14, bcd mRNAs become localised to embryonic P bodies and degraded. The authors use their data to modify the Synthesis, Diffusion, Degradation model of Bcd gradient formation, which recapitulates the Bcd gradient detected experimentally.

Overall, I think the data are of high quality and support the authors' conclusions.

We thank the Reviewer for their positive assessment and the helpful suggestions below.

I only have minor comments, as follows:

- Fig 1B - add arrows showing mRNAs being translated or not (the latter mentioned in line 113 is not so easy to see).

We have magnified the inset of the colocalisation in the right column; we have added arrows and arrowheads to differentiate colocalised and non-colocalised *bcd* with translating SunTag.

This is the edited part of Figure 1:

B

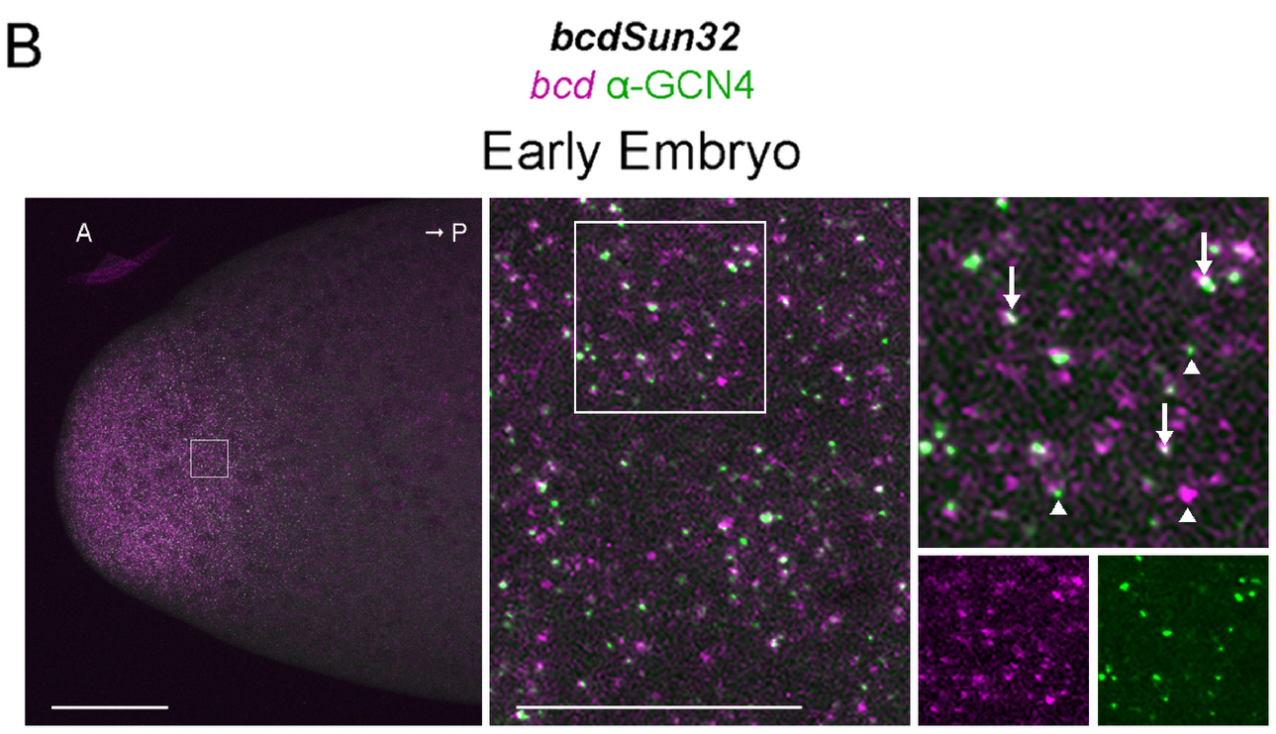

- Fig 2A - add a sentence explaining why 1,6HD, 2,5HD and NaCl disrupt P bodies.

We have added the information on the use of 1,6HD, 2,5HD, and NaCl to disrupt P-bodies on lines 158-169.

- Fig 4C - explain in the legend what the white lines drawn over the image represent. And why is there such an obvious distinction in the staining where suddenly the DAPI is much more evident (is

the image from tile scans)?

The original Figure 4C was the tile scan image of a n.c.10 embryo and the white line classified the image into four quadrants. We used this image to quantify the extent of *bcd* (magenta) colocalisation to SunTag (green) in the anterior and posterior domains of the embryo in the bar graph shown in panel C'. We have corrected a formatting error in the image and included the details of white lines in the legends. Based on further reviewer comments, in the revised version this data is shifted to the supplementary information (Figure S6).

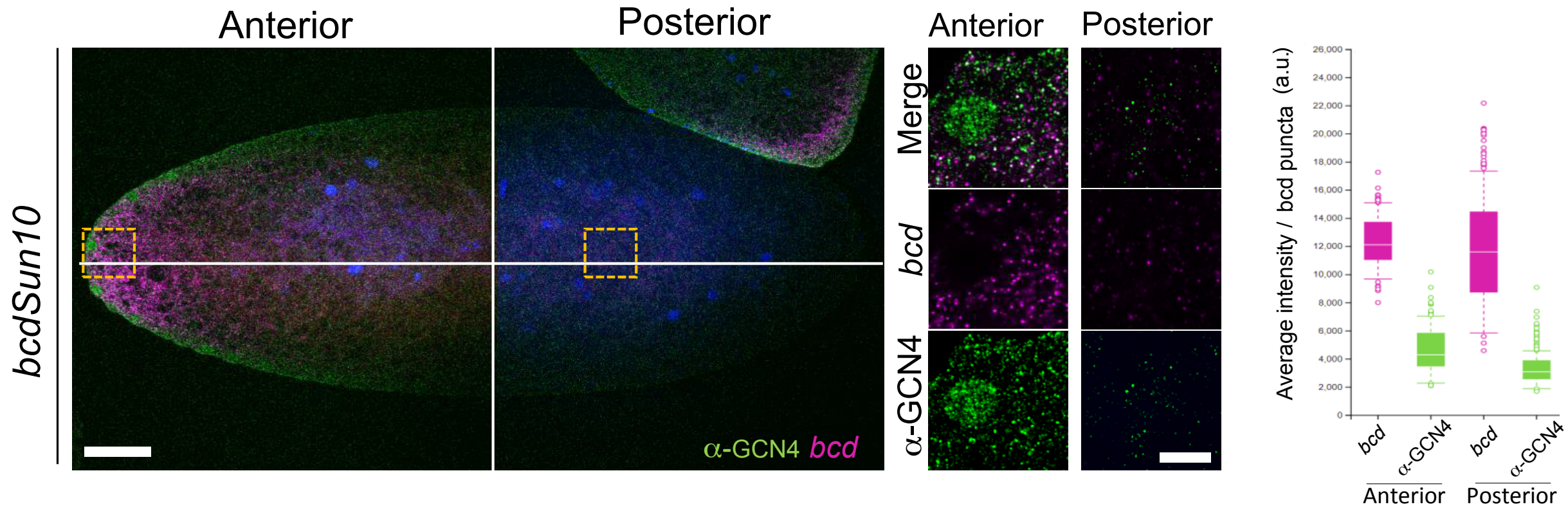

- Line 215 - 'We did not see any significant differences in the translation of bcd based on their position, however, there appears an enhanced translation of bcd localised basally to the nuclei (Figure S5).' Since the difference is not significant, I do not think the authors should conclude that translation is enhanced basally.

We agree with the reviewer. We have changed this statement to: "We did not see any differences in the translation of *bcd* based on their position with respect to the nuclei position (Figure S7)" (revised line 242-243).

- Line 218: 'The interphase nuclei and their subsequent mitotic divisions appeared to displace bcd towards the apical surface (Figure S6B).' Greater explanation is needed in the legend to Fig S6B to support this statement as the data just seem to show a nuclear division - I would have thought an apical-basal view is needed to conclude this.

We have rearranged this figure and shown in clarity the apical-basal view of the blastoderm nuclei and the displacement of *bcd* from the surface of the blastoderm in (new) Figure S7 as shown below.

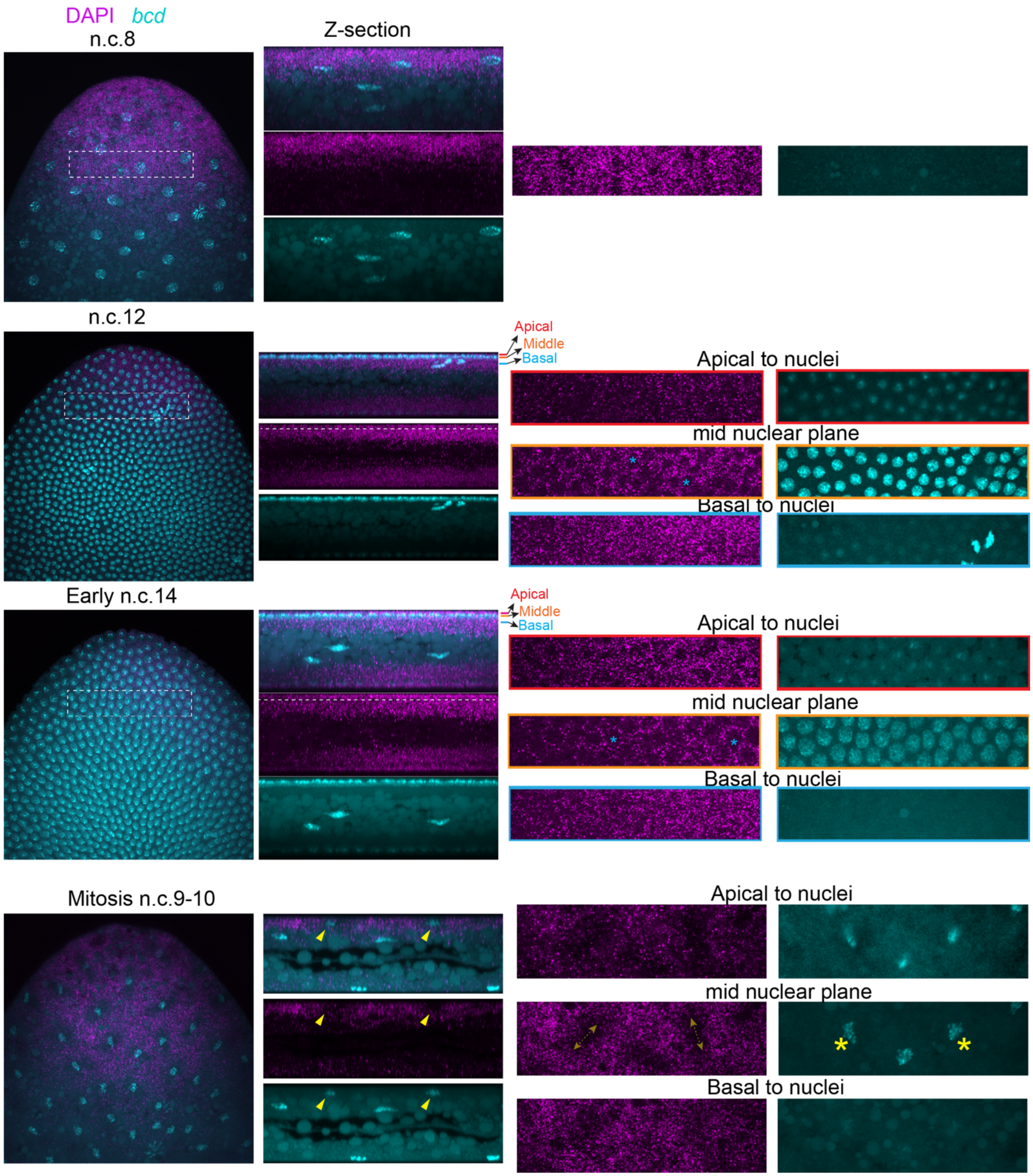

New Figure S7: n.c.8 - pre-cortical migration; n.c.12,14- post cortical migration; Mitosis stages of n.c.9-10. The cortical interphase nuclei at n.c. 12,14 displaces *bcd*. The nuclear area (DAPI, cyan) does not show any *bcd* particles (magenta) indicated by blue stars. The mitotic nuclei (yellow arrowheads, yellow stars) displace *bcd* along the plane of nuclear division (doubled headed yellow arrows).

- Fig 5B - the authors compare Bcd protein distribution across developmental time. However, in the early time points cytoplasmic Bcd is measured (presumably as it does not appear nuclear until nc8 onwards) and compare the distribution to nuclear Bcd intensities from nc9 onwards. Is most/all of

the Bcd protein nuclear localised form nc9 to validate the nuclear quantitation? Does the distribution look the same if total Bcd protein is measured per volume rather than just the nuclear signal? Are the authors assuming a constant fast rate of nuclear import?

From n.c. 8 onwards, the Bcd signal in interphase nuclei builds up, with the nuclear intensity becoming very high compared to cytoplasmic Bcd. However, we do see significant Bcd signal in the cytoplasm (*i.e.,* above background). In earlier work, gradients of the nuclear Bcd and nuclear-import mutant Bcd overlapped closely (Figure 1B, Grimm et al., 2010). This essentially suggests the nuclear Bcd gradient reflects the corresponding gradient of cytoplasmic Bcd. Further, the nuclear import of Bcd occurs rapidly after photobleaching (Gregor et al., 2007). Based on these observations, and our own measurements, prior to n.c. 9, the cytoplasmic gradient is likely a good approximation of the overall shape, whereas post n.c. 9 the Bcd signal is largely nuclear localised. Further, the overall profile is not dependent on the nuclear volume. We have revised Figures 4 and 5 to include more detail on the spread of the Bcd gradient.

- Line 259 - 'We then asked if considering the spatiotemporal pattern of bcd translation' - the authors should clarify what new information was included in the model. Similarly in line 286, 'By including more realistic bcd mRNA translation' - what does this actually mean?
We have clarified this on lines 310-311.

In line 346, 'We see that the original SDD model .... was too simple.' It would be nice to compare the outputs from the original vs modified SDD models to support the statement that the original model was too simple.
We have improved the linking of the results to the model (revised lines 321-344). The important point is that when and where Bcd production occurs is more faithfully used, compared with previous approximations. By including more realistic production domains, we can replicate the observed Bcd gradient within the SDD paradigm without resorting to more complex models. In Figure S9C, we show a comparison of the SDD and modified SDD models.

- Fig S1A - clarify what the difference is between the 2 +HD panels shown.

The two +HD panels at stage 14 indicate that upon the addition of HD, there are no particles in 70% of the embryos, and 30% show reduced particles. This information has been included in the figure legend of figure 2s. “There are no particles in 70% of the embryos (left 5% 1,6 hexanediol stage 14 panel), and 30% show reduced particles (right 5% 1,6 hexanediol stage 14 panel).”

- Fig S2E - the graph axis label/legend says it is intensity/molecule. Since intensity/molecule is higher in the anterior for bcd RNAs, is this because there are clumps of mRNAs (in which case it's actually intensity/puncta)?

The density of mRNA is very high in the anterior pole; there is a chance that more than one *bcd* particle is within the imaged puncta (due to optical resolution limitations). We have changed the y-axis from average intensity per molecule to average intensity per puncta (Figure S6).

- Fig S4 - I think this line is included in error: '(B) The line plots of bcd spread on the Dorsal vs. Ventral surfaces.'

This is now corrected.

- In B, D, E - is the plot depth from the dorsal surface? I would have preferred to see actual mRNA numbers rather than normalised mRNAs. In Fig S4D moderate, from 10um onwards there are virtually no mRNA counts based on the normalised value, but what is the actual number? The equivalent % translated data in Fig S4E look noisy so I wonder if this is due to there being a tiny mRNA number. The same is true for Figs S4D, E 10um+ in the low region.

Beyond 10µm from the dorsal surface, the number of bcdsun10 counts is very low. It becomes negligible at the moderate and low domains. We now attach the actual counts of mRNA in all these domains as supplementary tables (Table S6-8) in the revised version.

Significance

General assessment

Strengths are: 1) the data are of high quality; 2) the study advances the field by directly visualising Bcd mRNA translation during early Drosophila development; 3) the data showing re-localisation of bcd mRNAs to P bodies nc14 provides new mechanistic insight into its degradation; 4) a new SDD model for Bcd gradient formation is presented. Limitations of the study are: 1) there was already strong evidence (but no direct demonstration) that bcd mRNA translation was associated with release from P bodies at egg activation; 2) it is not totally clear to me how exactly the modified SDD model varies from the original one both in terms of parameters included and model output.

This is the first direct demonstration of the translation of *bcd* mRNA released as a single mRNA from P bodies. Previously, we have shown that P bodies disruption releases single *bcd* from the condensates (31). We have captured a comprehensive understanding of the status of individual *bcd* translation events, from their release from P bodies at the end of oocyte maturation until the end of blastoderm formation.

The underlying SDD model – that of localised production, diffusion, and degradation – is still the same (up to spatially varying diffusion). Yet the model as originally formulated did not fit all aspects of the data, especially with regards to the system dynamics. Here, we demonstrate that by including more accurate approximations of when and where Bcd is produced, we can explain the formation of the Bcd morphogen gradient without recourse to any further mechanism. We have clarified this on revised lines 331-344 and 406-412.

Reviewer #2

Evidence, reproducibility and clarity

In this manuscript by Athilingam et al., the authors are studying the translation of the morphogen Bicoid (Bcd), which is in anterior-posterior patterning of the blastoderm Drosophila embryo. They have used an array of sunTag elements in the 5' UTR of Bcd to detect the localization of translation. They found that, not only is Bcd not translated until egg activation, but it can only be translated at the anterior pole, even though bcd mRNA has a broader spatial distribution.

In general, the paper uses a cutting-edge methodology to address one of the foundational questions of the best-studied morphogen gradient: namely, what is the spatial distribution of the Bcd source? Together with the dynamics of its spreading (which they addressed in a separate study

in 2024) and Bcd degradation, their results point to a modified form of the synthesis/diffusion/degradation (SDD) model of Bcd gradient formation, which they have analyzed in the final subsection of the results. However, there are several major issues that erode the validity and impact of the paper, most of which can be put into the category of vague explanations, missing information, or contradictory statements, making it hard to understand/verify what conclusions can be drawn. This is also coupled with vague figures and captions. We describe these, and a few minor issues, in detail below:

We thank the Reviewer for their detailed assessment and recognition of the paper achievements. We agree that the linking of results can be improved and we address these comments below.

- Line 114: The authors claim to have validated the SunTag using a fluorescent reporter, but do not show any data. Ref 60 is a general reference to the SunTag, and not the Bcd results in this paper. Perhaps place their data into a supplemental figure or movie?

To show the validation of our *bcdSun32* line, we have composed a new Figure S1 that shows the translating *bcdSun32* (magenta) colocalising to the ScFV-mSGFP2 (green). Yellow arrowheads in the zoom (right panel) points to the translating *bcdSun32* (magenta) and red arrowheads points to the untranslated *bcdSun32*. In addition, we have also shown the validation of *bcdSun32* with the anti-GCN4 staining in the main Figure 1B.

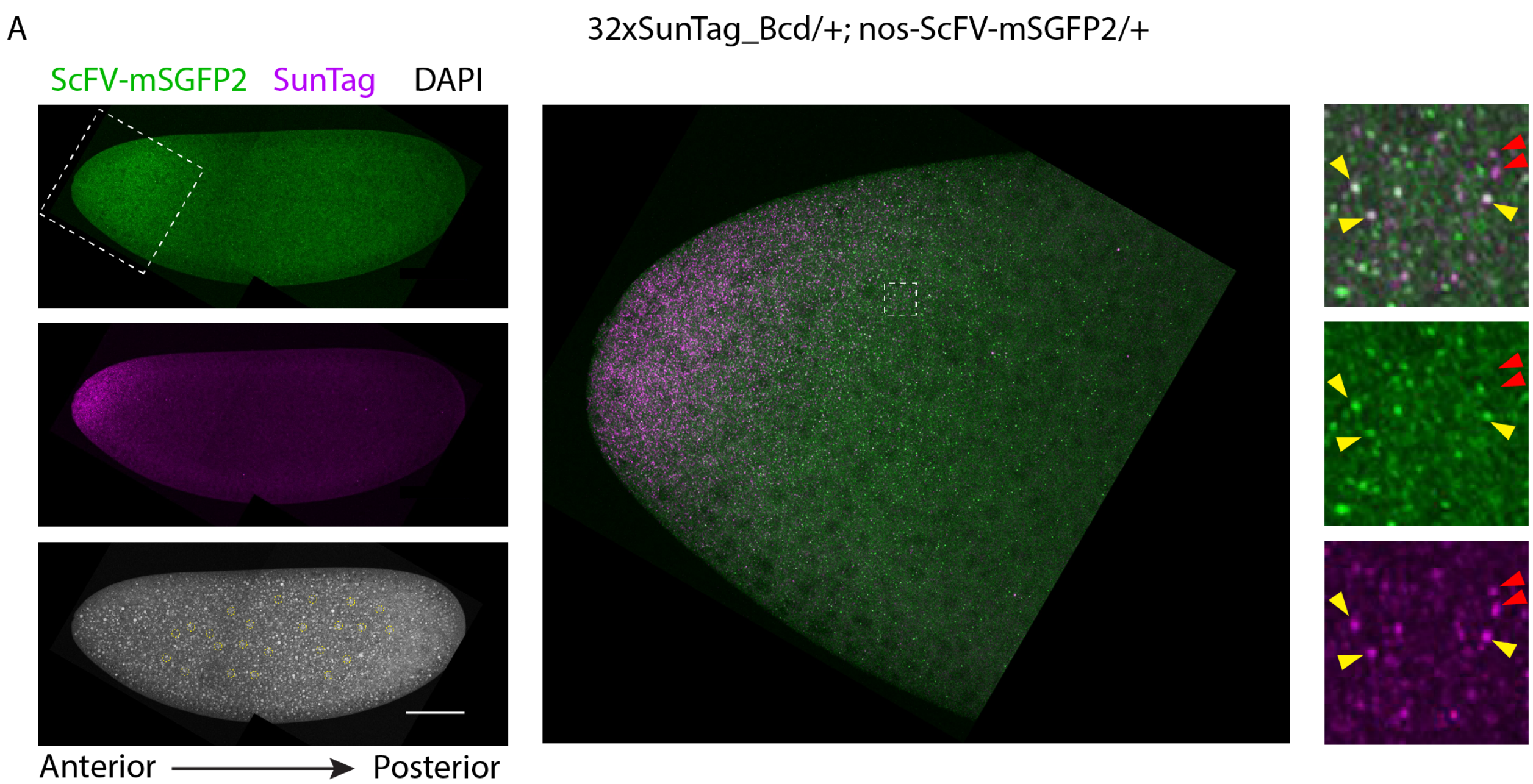

Further, we have dedicated Supplementary Figure S3 (previously Figure S2) for the validation of our *bcdSun10* construct. Briefly, *bcdSun10* is inserted into att40 site of chr.2. We did a rescue experiment, where *bcdSun10* rescued the lethality of homozygous *bcdE1* null mutant. We then performed a colocalisation experiment using smFISH, where we demonstrated that almost all *bcd* in the anterior pole are of type *bcdSun10*. We targeted specific fluorescent FISH probes against 10xSunTag sequence (magenta, Figure S3A) and bcd coding sequence (green, Figure S3A). Upon colocalisation, we found ~90% of the mRNA are of *bcdSun10* type. The remaining 10% could likely be contributed by the noise level (Figure S3B).

- Line 128 and Fig. 1E: The claim that bcd becomes dispersed is difficult to verify by looking at the image. The language could also be more precise. What does it mean to lose tight association? Perhaps the authors could quantify the distribution, and summarize it by a length scale parameter? This is one of the main claims of the paper (cf. Line 23 of the abstract) but it is described vaguely and tersely here.

We have changed the text from, "We also confirmed that *bcd* becomes dispersed, losing its tight association with the anterior cortex (Figure 1E) (31)" to, "We also confirmed that *bcd* is released from the anterior cortex at egg activation (Second panel, Figure 1E) (33)." (revised lines 133-135).

The release of *bcd* mRNA at egg activation was first shown in 2008 (Ref 21, Figure 4, D-E) and again in 2021 (Ref 31, Figure 7 B and E). The main point in line 132-133, "P bodies disassembled and *bcd* was no longer colocalised with P bodies" and the novel aspect of line 23 is "translation observed". The distribution of *bcd* mRNA after egg activation was not the point of this section. We have improved the writing in the revision to make this clearer.

- Line 146, Fig. 1G: This is a really important figure in the paper, but it is confusing because it seems the authors use the word "translation," when they mean "presence of Bcd protein." In other places in the paper, the authors give the impression that "bcd translation" means translation in progress (assayed by the colocalization of GCN4 and bcd mRNA). However, in Fig. 1G, the focus is only on GCN4. Detecting Bcd protein only at the anterior does not mean that translation happens only at the anterior (e.g., diffusion or spatially-restricted degradation could be in play).

In Figure 1G, we have shown only the "translated" Bcd by staining with a-GCN4. We have changed line 146 from, "Consistent with previous findings, we only observed *bcd* translation at the anterior of the activated egg and early embryo (Figure 1G-H) (3, 68)" to, "Consistent with previous findings, we only observed the presence of Bcd protein at the anterior of the activated egg and early embryo (Figure 1G-H) (3, 71) (revised lines 151-153). We now use "translating *bcd*" or "*bcd* in translation" where we show colocalisation of *bcd* with BcdSun10 or BcdSun32 elsewhere in the manuscript.

We did not mean to claim that translation occurred only in the anterior pole. We show that the abundance of *bcd* is very high in the anterior pole (in agreement with previous work) and that this is where the majority of observed translation events took place. Indeed, we have also shown that posteriorly localised mRNAs have the same BcdSun10 intensity per *bcd* puncta from the posterior pole (Figure 3B & 4C' and Figure S2 E), but these are much fewer in number.

- It would also be helpful to show a plot with quantification of Bcd detection (or translation) on the y-axis and a continuous AP coordinate on the x-axis, instead of just two points (anterior and posterior poles, the latter of which is uninteresting because observing no Bcd at the posterior pole is expected).

In Figure 1G,H, our aim was to test whether release from P bodies allowed for *bcd* mRNA to be translated. We used the presence of Bcd protein at the anterior domain of the oocytes to show this. The posterior pole was included as an internal control. To show the spatial distribution of *bcd* mRNA and its translation, we used early blastoderm (Figure 4 Figure S5).

- Another issue with Fig. 1G is that the A and P panels presumably have different brightness and

contrast. If not, just from looking at the A and P panels, the conclusion would be that Bcd protein is diffuse (and abundant) in the posterior and concentrated into puncta in the anterior. The authors should either make the brightness and contrast consistent or state that the P panel had a much higher brightness than the A panel.

We agree with this shortcoming. We have now added the following to Figure 1 legend to clarify this observation. "G: Representative fixed 10 µm Z-stack images (from 10 samples) showing BcdSun32 protein (anti-GCN4) is only present at the anterior of an *in vitro* activated egg or early embryo 30-minute post fertilization. BcdSun32 protein is not detected in these samples at the posterior pole (image contrast increased to highlight the lack of distinct particles at the posterior). BcdSun32 protein is also not detected at the anterior or posterior of a mature oocyte or an *in vitro* activated egg incubated with NS8953 (images have the contrast increased to highlight the lack of distinct particles). Scale bar: 20 µm; zoom 2 µm." (Revised figure legend of Figure 1).

- Line 176: This section is very confusing, because at this point the authors already addressed the spatial localization of translation in Fig. 1G,H (see my above comment). However, here it seems the authors have switched the definition of translation back to "translation in progress." Therefore, the confusion here could be eliminated by addressing the above point.

In the revised version, we now use Bcd protein when shown with anti-GCN4 staining. We use "translating *bcd*" or "*bcd* in translation" where we show colocalisation of *bcd* with a-GCN4 (BcdSun10 or BcdSun32).

- Line 185: The sentence here is seemingly contradictory: "most...within 100 microns" implies that at least some are beyond 100 microns, while the sentence ends with "[none]...more than 100 microns." The language could perhaps be altered to be less vague/contradictory.

In the lower panel of Figure 3B, the posterior domain shows some particles. However, their number compared to *bcd* counts within 100 µm of the anterior pole is negligible (Figure 3C). Nonetheless, the few *bcd* particles we observe do seem to be under translation (quantified in Figure 4C' and Figure S6). We have clarified this in the revised manuscript (revised lines 224-237).

- Line 204: It would be really nice to have quantification of the translation events, such as curves of rate of translation as a function of a continuous AP coordinate, and a curve for each nc.

We now provide the results quantifying the translation events across the anterior- posterior axis for n.c. 0-4, 13 and 14 (Figure 4C). Once there has been significant Bcd Suntag production, we cannot easily extract a curve for the translation events as the protein signal adds to the translation signal.

Our colocalisation analysis is semi-automated. It includes an automated counting of the individual *bcd* particle counts and a manual judgement of the colocalised BcdSun10 protein (distinct spots, above noise) to *bcd* particles (Figure S4D). The *bcd* particle counts ran into thousands in each cyan square box (measuring 50µm radius and ~ 20µm deep from the dorsal surface). We selected three such boxes covering 150µm (continuously) from the anterior pole across A-P axis and 20µm deep of the flattened embryo mounts across D-V axis (Figure 3A-C, Figure S5). We have also scanned scarce particles in the posterior; however, *bcd* counts are very low compared to the anterior.

Further, in Figure 4 we repeated the same technique to measure translation of *bcd* particles in embryos at different nuclear cycles.

We have also shown continuous intensity measurements of *bcd* particles with their respective BcdSun10 gradient in Figure 4 across the A-P axis at different nuclear cycles. Here, we know BcdSun10 intensity is not only from the "translating" *bcd* (colocalised BcdSun10 to *bcd* particles) but also from the translated BcdSun10 freely diffusing (non-colocalised BcdSun10 to *bcd* particles). As asked by the reviewer, in the revised version we have included *bcd* counts and their translation status from anterior to posterior axis for each of the nuclear cycles (Tables S6-8).

In future work, we plan to generate MS2 tagged *bcdSun10* to measure the rates of translation in live across all nuclear cycles but this lies beyond the current manuscript.

- Line 209 and Fig 4C: The authors use the terms "intensity of translation events" or "translation intensity" without clearly defining them. From the figure (specifically from the y-axis label), it looks like the authors are quantifying the intensity per molecule (which is not clearly the same thing as "translation intensity"), but it would be nice if that were stated explicitly.

We have changed the results text to "the intensity of translation events" for explaining the results of Figure S6' (revised line 235-236).

- In addition, the authors again quantify only two points. This is a continuously frustrating part of the manuscript, which applies to nearly all figures where the authors looked only at two points in space. At a typical sample size of N = 3, it seems well within time constraints to image at multiple points along the AP axis.

In the revised manuscript, we quantify translation events from anterior to posterior poles, with three embryos analysed (Figure 4C). We focused on semi-automated identification of translation events to ensure that identification was robust. As pointed out above, due to the Bcd::10Suntag protein also being fluorescent, this interferes with a simple quantification, especially at later stages.

- Furthermore, it sounds like the authors are saying the "translation intensity" is the same in anterior and the posterior, which is counterintuitive. The expectation is that translation would be undetectable at the posterior end, in part because bcd mRNA would not be present. (Note that this expectation is even acknowledged by the authors on Line 185, which I comment on above, and also on Line 197). There should also be very low levels of Bcd protein (possibly undetectable) at the posterior pole. As such, the authors should explain how they think their claim of the same "translation intensities" in the anterior vs posterior fits into the bigger picture of what we know about Bcd and what they have already stated in the manuscript. They should also explain how they observed enough molecules to quantify at the posterior end. The authors should also disclose how many points are in each box in the boxplot. For example, the sample size is N = 3 embryos. In just three embryos, how many bcd/GCN4 colocalizations did the authors observe at the posterior end of the embryo?

In n.c. 4 in Figure 3, we saw few *bcd* particles in the posterior. We counted them for n.c.10 and quantified them in Figure S6. Our new measurements (highlighted above) have enabled us to more clearly clarify this (revised lines 229-237). Finally, we will also provide the number of *bcd* particle

counts and their colocalisation with a-GCN4 as Supplementary Tables 6-8.

- Line 215: The sentence that starts on this line seems self-contradictory: I cannot tell whether or not there is a difference in translation based on position.

We have not observed any difference in the translation of *bcd* particles depending on the position along the Z-axis (revised lines 242-243).

- Line 229: Long-ranged is a relative term. From the graph, one could state there is some spatial extent to the mRNA gradient, so it is unclear what the authors mean when they say it is not "long-ranged." Could the mRNA gradient be quantified, such as with a spatial length scale? This would provide more information for readers to make their own conclusions about whether it is long-ranged.

We have quantified the *bcd* mRNA gradient for each n.c. (Figure 5A-C; absolute *bcd* intensities in Figure 5A, left panel and the normalised intensities in Figure 5B. The length of the mRNA spread appears constant with the half-length maximum of ~75um across all nuclear cycles. Our conclusion of a long ranged Bcd gradient is based on the comparisons of the half-length maximum measurements of *bcd* particles and BcdSun10 (Figure 5D). We provide quantification of the decay length in Figure S8D.

However, we agree with the reviewer that that the phrase “long-ranged” is open to interpretation. Therefore, we have removed this terminology and given the lengths explicitly in the text where appropriate (e.g., lines 365-366).

- Line 230: When the authors claim the Bcd gradient is steeper earlier, a quantification of the spatial extent (exponential decay length scale) would be appropriate. Indeed, lambda as a function of time would be beneficial. It should also be placed in context of earlier papers that claim the spatial length scale is constant.

We have rewritten this section to avoid confusion. The measured decay length is consistent across n.c. 4 to 13 (new Figure S8D). In the original manuscript, we were making the point that the spread of Bcd was more restricted to the anterior in earlier cycles but recognise that this wasn’t clear.

- Lines 235-236: The two sentences that start on these two lines are vague and seemingly contradictory. The first sentence says there is a spatial shift, but the second sentence sounds like it is saying there is no spatial change. The language could be more precise to explain the conclusions.

We have revised lines 270-275.

Minor issues/typos (still must be addressed for content):

- Line 81: Probably meant "evolutionarily conserved"

Yes, we have changed, “P bodies are an evolutionarily cytoplasmic RNP granule” to, “P bodies are an evolutionarily conserved cytoplasmic RNP granule” (revised lines 84-85).

- Figure 1 legend: part B says "from 15 samples" but also says N = 20. Which is it, or do these

numbers refer to different things?

We have edited this from, “early embryo (from 15 samples)” to, “early embryo (from 20 samples)”. (Revised line 626).

- Line 217: migration of what?

Edited to “cortical nuclear migration”.

- Line 228: "early embryo" is vague. The authors should give specific time points or nuclear cycle numbers.

Edited to “nuclear cycles 1-8”.

- Line 301: Other locations in the paper say 75 microns or 100 microns.

We have made sure that scales are consistent. It is 100 um.

- Fig. 5: all images should be oriented such that the dorsal midline is on the upper half of the embryo/image.

We have now flipped the image to match.

- Fig. 5B: There are light tan and/or light orange curves (behind the bold curves) that are not explained.

It is the standard deviation and we have now clarified this.

- Fig. 5C: the plot says "normalized" but nowhere do the authors describe what the curves are normalized to. There is also no explanation for what the broad areas of light color correspond to.

Normalised to the *bcd* intensity maxima. We have now clarified this.

Significance
The results, if upheld, are highly significant, as they are foundational measurements addressing a longstanding question of how morphogen gradients are formed, using Bcd (the foundational morphogen gradient) as a model. They also address fundamental questions in genetics and molecular biology: namely, control of mRNA distribution and translation.

We thank reviewer 2 for highlighting the importance of our work in the field. We are confident that we have addressed the issues raised by Reviewer 2 with the new set of quantifications.

Reviewer #3
Evidence, reproducibility and clarity
This is a review of "Dynamics of bicoid mRNA localization and translation dictate morphogen gradient formation" by Athilingam et al. In this manuscript, the authors perform quantification of mRNA localization and translation of bicoid, spanning oogenesis through the maternal to zygotic transition, yielding a definitive characterization of Bicoid gradient formation. The experiments,

analysis, and interpretation are on the whole performed rigorously. I very much enjoyed this paper, partly for incorporating the aspects of bcd regulation during oogenesis, which compared to embryonic function of bcd is relatively under-studied. Also valuable is improving the characterization of how bcd expression is shut down at NC14. I have several major comments for revision, and a few minor comments. I should stress that none of the major comments are terrible but are intended to improve the impact/readability/flow of this nice manuscript. With the exception of a straightforward immunostaining experiment, all major comments constitute reworking of the model or the text.

Major Comments:

1) It is not evident from the main results and methods text that the new SDD model incorporates the phenomenon reported in figure 4B. From my reading, the parameter beta accounts for the Bcd translation rate, which according to figure 7B(ii) effectively switches from off to on around fertilization and thereafter remains constant. Figure 4B shows that the fraction of bcd mRNA engaged in translation decreases beginning around NC12/13, and this is one of the more powerful results that comes from monitoring translation in addition to RNA localization/abundance/stability. My expectation based on figure 4B would be that parameter beta should decrease over time beginning around 90-100 minutes and approach zero by ~150 minutes. This rate could be fit to the experimental data that yields figure 4B. The modeling should be repeated while including this information.

This is a good observation. Currently, the reduced rate of *bcd* translation is modelled by incorporating an increased rate of *bcd* mRNA degradation. Of course, this could also be reduced by a change in the rate of translation directly. As stated already, the beta parameter is the least well characterised. In the SI (Figure S9D), we now include a model where beta changes but not the mRNA degradation rate. We have improved the discussion to make this point clearer (revised lines 406-412). We note that to uncover the details here is a substantial project in its own right, as we need to optimise the live imaging still further – a project currently ongoing in the lab – but we briefly mention now in the manuscript (revised lines 250-254).

2) The presentation of the SDD model should be expanded to address how well the characteristic decay length fits A) measured Bcd protein distributions, B) measured at different nuclear cycles. This would strengthen the claim that the new SDD model better captures gradient dynamics given the addition of translation and RNA distribution. These experimental data already exist as reported in Figure 5. In the current Figure 7, panels D and D' add little to the story and could be moved to a supplement if the authors want to include it (in any case, please fix the typo on the time axis of fig 7D' to read "hours"). The model per cell cycle and the comparison of experimental and modeled decay lengths could replace current D and D'.

Originally, we kept discussion of the SDD model only to core points. In the revision, we have improved comparison with experimental data. We have moved Figures 7D-D’ to Figure S9A.

As outlined in the responses above, this paper reveals an important insight: the SDD model – with suitable modifications such as temporally restricted Bcd production – can explain all observed properties of Bcd gradient formation. Other mechanisms – such as *bcd* mRNA gradients – are not required. Our adapted SDD model fits the gradient profile well across early development, with largest difference at early time points (new Figure 7D).

We now include comparison with the SDD model (Figure S9C) (revised line 328).

We now include in Figure S8D quantification of the decay length of Bcd in our experiments. We show in Figure S8C an example fitting of the *bcd* mRNA profile with a Hill function.

3) The exposition of the manuscript would benefit significantly by including a section either in the introduction or the appropriate section of the results that defines the competing models for gradient formation. In the current version, these models are only cited, and the key details only come out late (e.g., lines 302 onward, in the Discussion). Nevertheless, some of the results are presented as if in dialog with these models, but it reads as a one-sided conversation. For instance: Figure 3. The undercurrent in this figure is the RNA-gradient model. In the context of this model, the results clearly show that translation of bcd is restricted to the anterior. Without this context, Figure 3 could read as a fairly unremarkable observation that translation occurs wherever there is mRNA. Restructuring the manuscript to explicitly name competing models and to address how experimental results support or detract from each competing model would greatly enhance the impact of the exposition.

We thank the reviewer for this suggestion. We have added the current models of Bcd gradient formation in the introduction section (revised lines 50-55) and have changed the narrative of results in the model section to make our conclusions clearer regarding the different models (lines 331-344).

4A) Related to point 3: The entire results text surrounding Figure 2 should be revised to include more detail about A) what specific hypotheses are being tested; and B) to critically evaluate the limitations of the experimental approaches used to evaluate these hypotheses. Hexanediol and high salt conditions are not named explicitly in the text, but the text touts these as "chemicals" that "disrupt P-body integrity." This implies that the treatments are specific to P-bodies. Neither of these approaches are only disrupting P Body integrity. This does not invalidate this approach, but the manuscript needs to state what hypothesis HD and NaCl treatment addresses, and acknowledge the caveats of the approach (such as the non-specificity and the assumptions about the mechanism of action for HD).

We have made the following edits to resolve this point. Revised lines 158-169: "To further show that *bcd* storage in P bodies is required for translational repression, we treated mature eggs with chemicals known to disrupt RNP granule integrity. (33, 39, 72) (33, 73-75). Previous work has shown that the physical properties of P bodies in mature *Drosophila* oocytes can be shifted from an arrested to a more liquid-like state by addition of the aliphatic alcohol hexanediol (HD) (76-78). While 1,6 HD has been widely used to probe the physical state of phase-separated condensates both *in vivo* and *in vitro* (79-81), in some cells it appears to have unwanted cellular consequences (82). These include a potentially lethal cellular consequences that may indirectly affect the ability of condensates to form (78) and wider cellular implications thought to alter the activity of kinases (83). While we did not observe any noticeable cellular issues in mature *Drosophila* oocytes with 1,6 HD, we also used 2,5 HD, known to be less problematic in most tissues (82) and the monovalent salt sodium chloride (NaCl), which changes electrostatic interactions (76)."

4B) Continuing the comment above: it is good that the authors checked that HD and NaCl treatment does not cause egg activation. But no one outside of the field of Drosophila egg

activation knows what the 2-minute bleach test is and shouldn't have to delve into the literature to understand this sentence. Please explain in one sentence that "if eggs are activated, then x happens following a short exposure to bleach (citations). We exposed HD and NaCl treated eggs to bleach and observed... ."

We have made the following edits to resolve this point. Revised lines 171-180: "After treating mature eggs with these solutions, we observed BcdSun32 protein in the oocyte anterior (Figure 2A-B). One caveat to this experiment could be that treating mature eggs with these chemicals results in egg activation which would in turn generate Bcd protein. To eliminate this possibility, we first screened for phenotypic egg activation markers, including swelling and a change in the chorion (84). We also applied the classic approach of bleaching eggs for two minutes which causes lysis of unactivated eggs (85). All chemically treated eggs failed this bleaching test meaning they were not activated (85). While we unable to rule out non-specific actions of these treatments, these experiments corroborate that storage in P bodies that adopt an arrested physical state is crucial to maintain *bcd* translational repression (33)."

4C) Continuing the comment above: The section of the results related to the endos mutation needs additional information. It is not apparent to the average reader how the endos mutation results in changes in RNP granules, nor what the expected outcome of such an effect would "further test the model" set up by the HD and NaCl experiments. The average reader needs more hand-holding throughout this entire section (related to figure 2) to follow the exposition of the results.

We have made the following edits to resolve this point. Revised lines 182-193: "Finally, we used a genetic manipulation to change the physical state of P bodies in mature oocytes. Mutations in *Drosophila* Endosulfine (Endos), which is part of the conserved phosphoprotein α-endosulfine (ENSA) family (86), caused a liquid-like P body state after oocyte maturation, similar to that observed with chemical treatment (Figure 2C) (33). This temporal effect matched the known roles of Endos as the master regulator of oocyte maturation (86, 87). *endos* mutant oocytes lost the colocalisation of *bcd* mRNA and P bodies, concurrent with P bodies becoming less viscous during oocyte maturation (Figure 2D, Figure S1). Particle size and position analysis showed that *bcd* mRNA prematurely exhibits an embryo distribution in these mutants (Figure 2E). Due to genetic and antibody constraints, we are unable to test for translation of *bcd* in the *endos* mutant. However, it follows that *bcd* observed in this diffuse distribution outside of P bodies would be translationally active (Figure 2E-F)."

4D) Continuing the comment above: The average reader also needs a better explanation of what hypothesis is being tested in Figure 1 with the pharmacological inhibition of calcium.

We have made the following edits to resolve this point. Revised lines 140-148: "We next sought to maintain the relationship between *bcd* mRNA and P bodies through egg activation. This would act as a control to further test if colocalisation of *bcd* to P bodies was necessary for its translational repression. Previous work has shown that a calcium wave is required at egg activation for further development (30, 68, 69). Chemical treatment with NS8593 disrupts this calcium wave, while other phenotypic markers of egg activation are still observed (58). Using NS8593 to disrupt the calcium wave in the activated egg, we show P bodies are retained during *ex vivo* egg activation (Figure 1E). In these treated eggs, *bcd* mRNA remains colocalised with the retained P bodies (Figure 1F) (33, 70), we hypothesised that the loss of colocalisation between *bcd* and P bodies correlates with *bcd* translation."

5) It is unclear why Bcd translation could not be measured in the endos mutant background, but it would be necessary to measure Bcd translation in the endos background. If genotypically it is not possible/inconvenient to invoke the suntag reporter in the endos background, would it not be sufficient to immunostain against Bcd itself? Different Bcd antisera have recently been reported and distributed by the Wieschaus and the Zeitlinger groups.

We tested the Bcd antibody that was kindly shared by the Zeitlinger group. This has not been shown to work for immunostaining. It was successful in labelling Bcd in the cellularised embryo, ~ 3 hour (panel A). However, there was no distinguishable signal observed in 0-2 hour embryos (panel B and C). The images displayed in B and C have been enhanced to show that the fluorescence detected was not localised to the anterior and appeared to be imaging noise. Despite this, we tested the antibody on Endos mutant stage 14 egg chambers (panel D, enhanced signal). We did not detect any clear Bcd protein in these samples. However, this does not mean that *bcd* mRNA has not been translated in these samples because the positive control (panel B and C) also failed to detect protein. Therefore, we have not included any of this in the manuscript, as it is inconclusive.

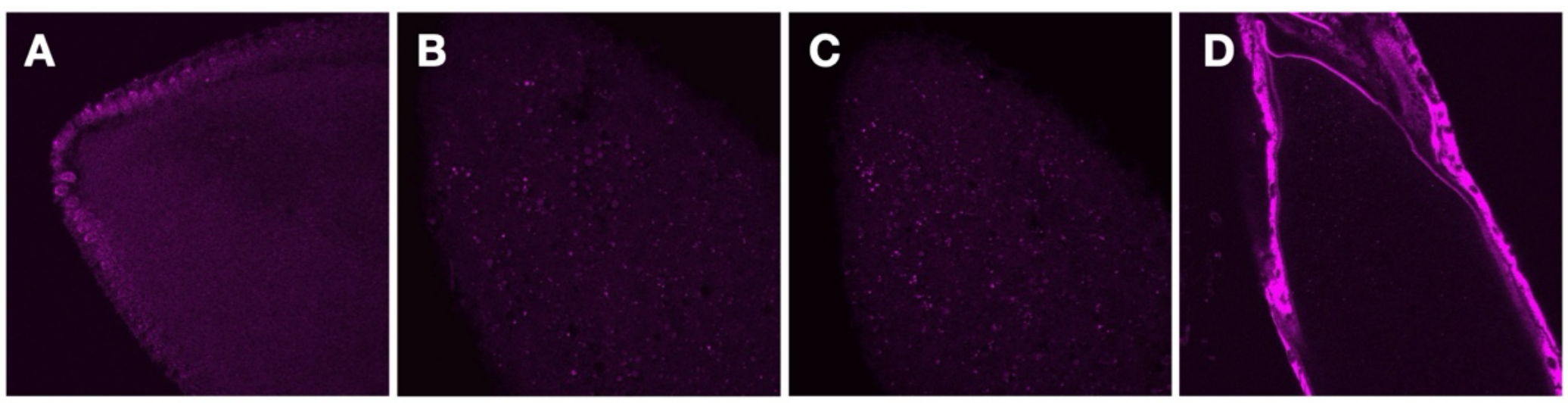

Reviewer Figure: Bcd antibody staining of *Drosophila* embryos (A-C) and stage 14 egg chamber (D).

6) Figure 4 overall is glorious, but there is a problem with panel C. What are the white lines? Why does the intensity for the green and magenta channel change abruptly in the middle of the embryo?

These white lines divide the embryo into 4 compartments. We used this method to quantify the intensity of Bcd translation with respect to the *bcd* puncta. We have corrected this image as there was a formatting issue and it is now in the Supplementary (Figure S6).

7) It is noted that neither the methods section or the supplement does not contain any mention of how the modeling was performed. How was parameter beta fit? At least a brief section should be added to the methods describing how beta was fit (pending adjustments suggested in comment 1 above). A platinum-level addition would include a modeling supplement that reports the sensitivity of model outcomes to changes in parameters.

We now include methodological details in the revised Methods, with extra material in Figure S9. Further, we will post the code onto our GitHub page: https://github.com/TimSaundersLab

Minor Comments:

- Line 28: "Source-Diffusion-Degradation" should be changed to "Synthesis-..."

We thank the reviewer for spotting this! We have revised.

- Line 39: "blastocyst" should be "blastoderm stage embryo".

We have updated.

- Line 81: "P bodies are an evolutionarily cytoplasmic RNP granule." is "conserved" missing here?

We have updated.

- Throughout the manuscript, there should be better reporting of the imaged genotypes and whether the suntag is being visualized by indirect immunostaining of fixed tissues or through an encoded nanobody-GFP fusion.

We have clarified details where appropriate, including in figure legends.

- Figure 1G: Why is the background staining so different across conditions? Is this a normalization artifact?

We agree with this shortcoming. We have now added the following to the figure legend to clarify this observation (revised lines 647-653). "G: Representative fixed 10 µm Z-stack images (from 10 samples) showing BcdSun32 protein (anti-GCN4) is only present at the anterior of an *in vitro* activated egg or early embryo 30-minute post fertilization. BcdSun32 protein is not detected in these samples at the posterior pole (image contrast increased to highlight the lack of distinct particles at the posterior). BcdSun32 protein is also not detected at the anterior or posterior of a mature oocyte or an *in vitro* activated egg incubated with NS8953 (images have the contrast increased to highlight the lack of distinct particles). Scale bar: 20 µm; zoom 2 µm."

- Figure 2 legend: what is +Sch in the x-axis labels of figure 2B? The legend says that 2B is the quantification of the data in 2A, but there is no (presumed control) +Sch image in 2A.

Thank you for this suggestion we have added the data to Figure 2A:

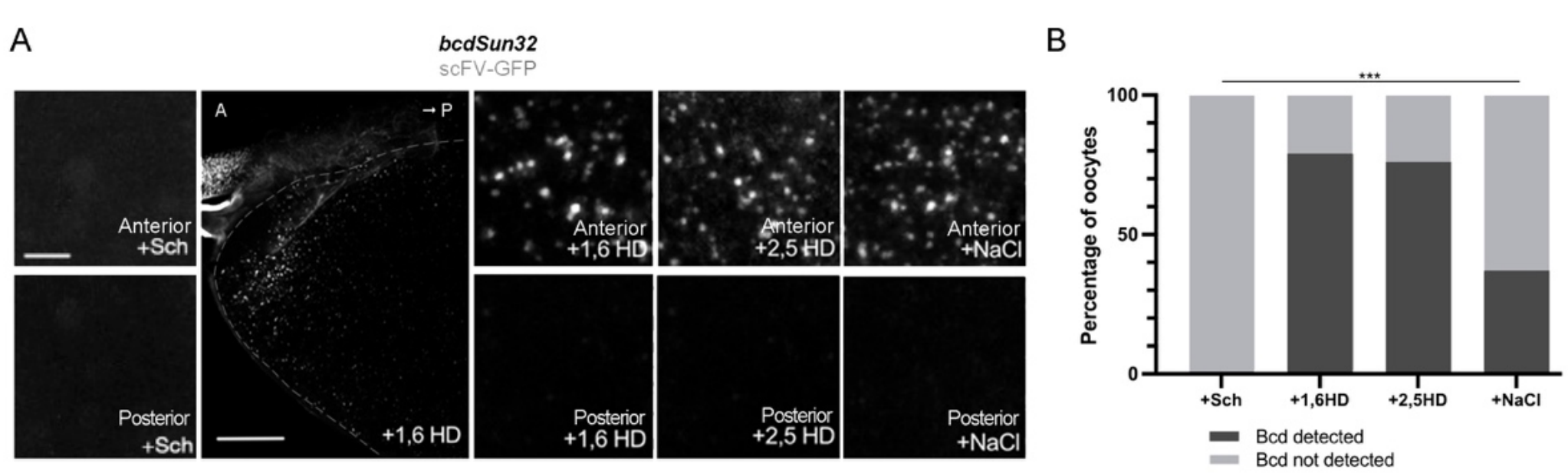

- Figure 5A largely repeats information presented in figure 4A. Please consider moving to a supplement. Also, please re-orient embryos to follow the convention that dorsal-most surfaces be presented on the top of the displayed images.

We agree that the data is largely repetitive, and so we have moved to a Supplementary Figure. This has improved readability by reducing the size of Figure 5.

- The lower-case roman numerals referred to in the text for figure 7B are not included in the corresponding figure panel.

We have edited in the revised version.

- Figure 7C y-axis typo (concentration).

Thank you for spotting this!

- Line 222: "make a long-range functional gradient": more accurate to say, "but also marks mature, Bcd protein which resolves in the expected long-range gradient."

We have edited this – see also comment from Reviewer 2.

- Methods: Please check that all buffers referred to as acronyms are both compositionally defined in the reagents table, and that full names are written out at the time of first mention in the presented order. For instance, Schneider's media is referred to a few times before defining the acronym about midway through the methods section.

We have added the following to Figure 2 legend, A. "Schneider's Insect Medium (+Sch) used as a control" (revised lines 665-666) and B. "Quantification of experiments shown in A. The number of oocytes that displayed Bcd protein at the anterior as measured by the presence of BcdSun32 at the anterior of the oocyte, but not the posterior. Schneider's Insect Medium (+Sch) used as a control. N = 30 oocytes for each treatment. Scale bar: 5 um" (revised lines 670-673).

Significance

The importance of this study is at several levels. For the developmental biologist, it addresses important mechanisms of translational control and RNA stability over the functional lifetime of a single, critical biological cue that governs embryonic patterning. Not only do the experiments provide quantification of these features, but also point to likely candidates (P-bodies) for gating bcd's translation in the narrow window between egg activation and cellular blastoderm. For the biophysically-inclined, this adds critical quantitative information of translational state that allows for further refining computational models for how this manifestation of a reaction-diffusion system actually comes together in a complex biological context.

The primary audience for this work will be the two groups above: developmental biologists and scientists interested in the quantitative modeling of biological phenomena.

Dear Dr. Weil,

Thank you for submitting a revised version of your manuscript to The EMBO journal. Your study has now been seen by two of the original referees, who find that their previous concerns have been sufficiently addressed and now recommend publication of the manuscript after minor revisions.

In addition to the remaining reviewer requests, there are a few editorial points that need to be addressed before I can extend the official acceptance of the manuscript:

1. Please note that it is The EMBO Journal policy for the transcript of the editorial process (containing referee reports and your response letters) to be published as an online supplement to each paper. If you should prefer removal of any referee-only figures included in the point-by-point response(s), e.g. because they may still be used for future publication or because they have been reproduced from published work by others, please do let us know immediately via response email.
More information is available here: https://www.embopress.org/transparent-process#Review_Process
2. Emails to the author Elise L Wilby (elw63@cam.ac.uk) was returned. Please check for correctness.
3. Please spell out the complete names of the authors on the manuscript title page, they should be listed here in the form in which they should be published.
4. Please ensure that the list of funders entered into our system is complete and accurate; the list will be linked to the corresponding database in PubMed.
5. Please place the Methods section after the Discussion and the Figure Legends section after the References.
6. CRediT has replaced the traditional author contributions section because it offers a systematic, machine-readable author contributions format that allows for more effective research assessment. Please remove the Authors Contributions from the manuscript and use the free text boxes beneath each contributing author's name in our online submission system to add specific details on the author's contribution. More information is available in our guide to authors.
7. Please add a "Disclosure and competing interests statement" section after "Acknowledgements".
8. Please update references according to The EMBO Journal style - it should be alphabetical; where there are more than 10 authors on a paper, the first 10 should be listed, followed by 'et al.'
9. We require a Data Availability Section at the end of Materials and Methods. As far as I can see, no data deposition in external databases is needed for this paper. If I am correct, then please state in this section: This study includes no data deposited in external repositories. Further information can be found at https://link.springer.com/partners/embo-press/editorial-policies#Data%20availability%20statement.
10. Please remove the Reagents and Tools table from the manuscript and upload as a separate file it as a separate file choosing the file type "Reagent Table".
11. Figure panel 2G is not mentioned in the manuscript text, please check and correct.
12. There is a PDF with supplementary figures and tables. These can either be made Expanded View (EV) figures and tables or remain in the PDF as Appendix figures and tables. In the latter case, a table of contents should be added to the PDF including page numbers, and the nomenclature should be corrected to "Appendix Figure S1" etc. and "Appendix Table S1" etc.
If you choose to turn the figures into EV figures, they should be cited as 'Figure EV1, Figure EV2" etc. in the text and their respective legends should be included in the main text after the legends of regular figures. EV Tables should be uploaded as individual files with their legends provided in a separate tab in case of .xls files.
13. At EMBO Press we ask authors to provide source data for the main manuscript figures. You had received a separate email on February 2, 2026, with instructions for providing source data with your revised manuscript, including how to upload and organise the files. Please submit these files with the final version.
14. During our standard image check, we noticed possible reuse between the following figure panels:
- Within Figure 1G activated egg/early embryo panels - these images could be derived from the same sample.
- Between Figure 6A (early NC-14 DAPI) and Appendix Figure S7 (n.c. 12).
- Between Appendix Figure S2A and Appendix Figure S2B, stage 13 panels.
Please check - if this was intentional, please clearly state this in the corresponding figure legends.
15. Our data editors have flagged the following issues in figure legends that need correcting:
- Please define the annotated p values *** as well as provide their exact values in the legend of figures 1F,H; 2B,D.
- Please provide the exact p values in the legends of 2F,G; 4B; 6D-F.
- Please indicate the statistical test used for data analysis in the legends of figures 4B; 6D-F.
- Please note that the box plots need to be defined in terms of minima, maxima, centre, bounds of box and whiskers, and percentile in the legends of figures 6E,F.
- Please define the error bars in the legends of 2F,G; 5C,D.
- Please define the measure of centre for the error bars in the legends of figures 4B,C.
- Please define the white arrowheads and arrows in the legend of figure 1B.
- Please define the yellow and red arrowheads are not defined in the legend of figure 3A.
16. Papers published in The EMBO Journal are accompanied online by a 'Synopsis' to enhance discoverability of the manuscript. It consists of A) a short (1-2 sentences) summary of the findings and their significance, B) 3-4 bullet points highlighting key results (the highlights currently included in the manuscript can be used here) and C) a synopsis image that is 550x300 pixels large (width x height, jpeg or png format). You can either show a model or key data in the synopsis image.

Please note that the image size is rather small and that text needs to be readable at the final size.

Please let me know if you have any questions regarding any of these points. You can use the link below to upload the revised files.

Thank you again for giving us the chance to consider your manuscript for The EMBO Journal. I look forward to receiving the final version.

With best wishes,

Ieva

Ieva Gailite, PhD
Senior Scientific Editor
The EMBO Journal
Meyerhofstrasse 1
D-69117 Heidelberg
Tel: +4962218891309
i.gailite@embojournal.org

Revision to The EMBO Journal should be submitted online within 90 days, unless an extension has been requested and approved by the editor; please click on the link below to submit the revision online before 15th Sep 2026:

Link Not Available

Referee #2:

I appreciate the authors' attention to the reviewers' comments. The manuscript is greatly improved in its clarity and readability. Furthermore, the new quantification they performed is also useful. However, I did notice that two of my comments seem to have been accidentally left unaddressed (even though the rebuttal letter claims they were addressed). In particular:
- Line 217: migration of what? [Now line 243]. The authors claim that they changed this to "cortical nuclear migration", but it was not changed.
- Line 228: "early embryo" is vague. The authors should give specific time points or nuclear cycle numbers. [Now line 263]. The authors claim that they changed this to "nuclear cycles 1-8", but it was not changed.
I trust these two changes can be made easily in their final submission without the need to review the manuscript further.

Referee #3:

I have reviewed the revised manuscript and my prior comments and find that the authors have satisfactorily addressed my outstanding issues. I recommend publication.

***
Rev_Com_number: RC-2025-03245
New_manu_number: EMBOJ-2026-123675R
Corr_author: Weil
Title: Dynamics of bicoid mRNA localisation and translation dictate morphogen gradient formation

The two points from reviewer 2 should be straight-forward.

- Line 217: migration of what? [Now line 243]. The authors claim that they changed this to "cortical nuclear migration", but it was not changed.

In the current line 252 we have changed the line "…. noticed that upon migration of nuclei to the cortex, *bcd* localisation…."

- Line 228: "early embryo" is vague. The authors should give specific time points or nuclear cycle numbers. [Now line 263]. The authors claim that they changed this to "nuclear cycles 1-8", but it was not changed.

In the current line 273, we changed the line "….The extent of *bcd* mRNA was localised to the anterior during n.c.1 to 8, with some dispersal post cortical nuclear migration (Figure 5B)…."

14. During our standard image check, we noticed possible reuse between the following figure panels:
- Within Figure 1G activated egg/early embryo panels - these images could be derived from the same sample.

These were not derived from the same sample.

- Between Figure 6A (early NC-14 DAPI) and Appendix Figure S7 (n.c. 12).

We have changed Appendix Figure S7 with a new sample for n.c.12.

- Between Appendix Figure S2A and Appendix Figure S2B, stage 13 panels.

These are the same image and that information has been added to the figure legend.

15. Our data editors have flagged the following issues in figure legends that need correcting:
- Please define the annotated p values *** as well as provide their exact values in the legend of figures 1F,H; 2B,D.

We have added p-values, in the legends of figures wherever required.

- Please provide the exact p values in the legends of 2F,G; 4B; 6D-F.

The P-values are given in the legends of 2F,G; 4B; 6D-F.

- Please indicate the statistical test used for data analysis in the legends of figures 4B; 6D-F.

The details of the statistical tests are given in the legends of 4B; 6D-F.

- Please note that the box plots need to be defined in terms of minima, maxima, centre, bounds of box and whiskers, and percentile in the legends of figures 6E,F.

The details are provided in the legends of figures 6E,F.

- Please define the error bars in the legends of 2F,G; 5C,D.

Edited figure 5C and D legends for clarity.

- Please define the measure of centre for the error bars in the legends of figures 4B,C.

Edited figure 4 legends to include the details of the plot.

- Please define the white arrowheads and arrows in the legend of figure 1B.

Added the details in the legend of figure 1B about the arrows and arrowheads.

- Please define the yellow and red arrowheads are not defined in the legend of figure 3A.

Added the detail of the orange and red arrowheads in the legend of figure 3A.

2nd Revision - Editorial Decision **26th Aug 2026**

Dear Dr. Weil,

Thank you for addressing the editorial and formatting requests in the final version of your manuscript. I am now pleased to inform you that your manuscript has been accepted for publication. Congratulations on a nice study!

Before we forward your manuscript to the publishers, there remain a few points that I need your input on:
1. I still need from you a brief synopsis text consisting of a short (1-2 sentences) summary paragraph of the findings and their significance, followed by 3-4 bullet points highlighting key results.
2. We also need a synopsis image that is 550x300 pixels large (width x height, jpeg or png format), which can show either a model of the findings or key data.
3. I would propose to somewhat simplify the description of the combined violin-box plots in the legend for panels 6E-F. The description could be along the following lines: "In the box-and-whisker plot, the box extends from the 25th to the 75th percentile, the whiskers show the minimum and maximum values, and the line within the box represents the median. The overlaid violin plot indicates the distribution of the data, with minima and maxima of the shaded area representing the data range." Please correct as needed - I will be happy to include the description in the figure legend for you. it is not required to list the number of the data points within each category.

Your manuscript will be processed for publication by EMBO Press. It will be copy edited and you will receive page proofs prior to publication. Please note that you will be contacted by Springer Nature Author Services to complete licensing and payment information.

You may qualify for financial assistance for your publication charges - either via a Springer Nature fully open access agreement or an EMBO initiative. Check your eligibility: https://link.springer.com/journal/44318/how-to-publish-with-us

Should you be planning a Press Release on your article, please notify the production team via this form in order to coordinate publication and release dates: https://forms.cloud.microsoft/e/eH4A8JVP6g

I look forward to hearing from you on the remaining requests soon, after which we will be able to proceed with the transfer of your manuscript for typesetting.

Thank you for this interesting contribution to The EMBO Journal!

Best wishes,

Ieva

Ieva Gailite, PhD
Senior Scientific Editor
The EMBO Journal
Meyerhofstrasse 1
69117 Heidelberg
i.gailite@embojournal.org

*****
Please note that it is The EMBO Journal policy for the transcript of the editorial process (containing referee reports and your response letters) to be published as an online supplement to each paper. If you should prefer removal of any referee-only figures included in the point-by-point response(s), e.g. because they may still be used for future publication or because they have been reproduced from published work by others, please do let us know immediately via response email.
More information is available here: https://link.springer.com/partners/embo-press/editorial-policies#Peer%20review

EMBO Press Author Checklist

Corresponding Author Name: Timothy T Weil	
Journal Submitted to: The EMBO Journal	
Manuscript Number: EMBOJ-2026-123675R	

USEFUL LINKS FOR COMPLETING THIS FORM
The EMBO Journal - Author Guidelines
EMBO Reports - Author Guidelines
Molecular Systems Biology - Author Guidelines
EMBO Molecular Medicine - Author Guidelines

Reporting Checklist for Life Science Articles (updated January
This checklist is adapted from Materials Design Analysis Reporting (MDAR) Checklist for Authors. MDAR establishes a minimum set of requirements in transparent reporting in the life sciences (see Statement of Task: 10.31222/osf.io/9sm4x). Please follow the journal's guidelines in preparing your
Please note that a copy of this checklist will be published alongside your article.

Abridged guidelines for figures
1. Data
The data shown in figures should satisfy the following conditions:

- the data were obtained and processed according to the field's best practice and are presented to reflect the results of the experiments in an accurate and unbiased manner.
- ideally, figure panels should include only measurements that are directly comparable to each other and obtained with the same assay.
- plots include clearly labeled error bars for independent experiments and sample sizes. Unless justified, error bars should not be shown for technical
- if n<5, the individual data points from each experiment should be plotted. Any statistical test employed should be justified.
- Source Data should be included to report the data underlying figures according to the guidelines set out in the authorship guidelines on Data

2. Captions
Each figure caption should contain the following information, for each panel where they are relevant:

- a specification of the experimental system investigated (eg cell line, species name).
- the assay(s) and method(s) used to carry out the reported observations and measurements.
- an explicit mention of the biological and chemical entity(ies) that are being measured.
- an explicit mention of the biological and chemical entity(ies) that are altered/varied/perturbed in a controlled manner.
- the exact sample size (n) for each experimental group/condition, given as a number, not a range;
- a description of the sample collection allowing the reader to understand whether the samples represent technical or biological replicates (including how many animals, litters, cultures, etc.).
- a statement of how many times the experiment shown was independently replicated in the laboratory.
- definitions of statistical methods and measures:
  - common tests, such as t-test (please specify whether paired vs. unpaired), simple $\chi 2$ tests, Wilcoxon and Mann-Whitney tests, can be unambiguously identified by name only, but more complex techniques should be described in the methods section;
  - are tests one-sided or two-sided?
  - are there adjustments for multiple comparisons?
  - exact statistical test results, e.g., P values = x but not P values < x;
  - definition of 'center values' as median or average;
  - definition of error bars as s.d. or s.e.m.

Please complete ALL of the questions below.
Select "Not Applicable" only when the requested information is not relevant for your study.

Materials

Newly Created Materials	Information included in the manuscript?	In which section is the information available? (Reagents and Tools Table, Materials and Methods, Figures, Data Availability Section)
New materials and reagents need to be available; do any restrictions apply?	Yes	Materials and Methods

Antibodies	Information included in the manuscript?	In which section is the information available? (Reagents and Tools Table, Materials and Methods, Figures, Data Availability Section)
For **antibodies** provide the following information:<br>- Commercial antibodies: RRID (if possible) or supplier name, catalogue number and or/clone number<br>- Non-commercial: RRID or citation	Yes	Materials and Methods

DNA and RNA sequences	Information included in the manuscript?	In which section is the information available? (Reagents and Tools Table, Materials and Methods, Figures, Data Availability Section)
**Short novel DNA or RNA including primers, probes**: provide the sequences.	Yes	Data Availability Section, Materials and Methods

Cell materials	Information included in the manuscript?	In which section is the information available? (Reagents and Tools Table, Materials and Methods, Figures, Data Availability Section)
**Cell lines:** Provide species information, strain. Provide accession number in repository **OR** supplier name, catalog number, clone number, and/**OR** RRID.	Not Applicable	
**Primary cultures:** Provide species, strain, sex of origin, genetic modification status.	Not Applicable	
Report if the cell lines were recently **authenticated** (e.g., by STR profiling) and tested for mycoplasma contamination.	Not Applicable	

Experimental animals	Information included in the manuscript?	In which section is the information available? (Reagents and Tools Table, Materials and Methods, Figures, Data Availability Section)
**Laboratory animals or Model organisms:** Provide species, strain, sex, age, genetic modification status. Provide accession number in repository **OR** supplier name, catalog number, clone number, **OR** RRID.	Yes	Materials and Methods
**Animal observed in or captured from the field:** Provide species, sex, and age where possible.	Not Applicable	
Please detail **housing and husbandry conditions**.	Yes	Materials and Methods

Plants and microbes	Information included in the manuscript?	In which section is the information available? (Reagents and Tools Table, Materials and Methods, Figures, Data Availability Section)
**Plants:** provide species and strain, ecotype and cultivar where relevant, unique accession number if available, and source (including location for collected wild specimens).	Not Applicable	
**Microbes:** provide species and strain, unique accession number if available, and source.	Not Applicable	

Human research participants	Information included in the manuscript?	In which section is the information available? (Reagents and Tools Table, Materials and Methods, Figures, Data Availability Section)
If collected and within the bounds of privacy constraints report on age, sex and gender or ethnicity for all study participants.	Not Applicable	

Core facilities	Information included in the manuscript?	In which section is the information available? (Reagents and Tools Table, Materials and Methods, Figures, Data Availability Section)
If your work benefited from core facilities, was their service mentioned in the acknowledgments section?	Yes	

Design

Study protocol	Information included in the manuscript?	In which section is the information available? (Reagents and Tools Table, Materials and Methods, Figures, Data Availability Section)
If study protocol has been **pre-registered, provide DOI in the manuscript**. For clinical trials, provide the trial registration number **OR** cite DOI.	Not Applicable	
Report the **clinical trial registration number** (at ClinicalTrials.gov or equivalent), where applicable.	Not Applicable	

Laboratory protocol	Information included in the manuscript?	In which section is the information available? (Reagents and Tools Table, Materials and Methods, Figures, Data Availability Section)
Provide DOI OR other citation details if **external detailed step-by-step protocols** are available.	Yes	Materials and Methods

Experimental study design and statistics	Information included in the manuscript?	In which section is the information available? (Reagents and Tools Table, Materials and Methods, Figures, Data Availability Section)
Include a statement about **sample size** estimate even if no statistical methods were used.	Yes	Figures
Were any steps taken to minimize the effects of subjective bias when allocating animals/samples to treatment (e.g. **randomization procedure**)? If yes, have they been described?	Yes	Data Availability Section, Materials and Methods
Include a statement about **blinding** even if no blinding was done.	Yes	Data Availability Section, Materials and Methods
Describe **inclusion/exclusion criteria** if samples or animals were excluded from the analysis. Were the criteria pre-established?<br><br>If sample or data points were omitted from analysis, report if this was due to attrition or intentional exclusion and provide justification.	Not Applicable	
For every figure, are **statistical tests** justified as appropriate? Do the data meet the assumptions of the tests (e.g., normal distribution)? Describe any methods used to assess it. Is there an estimate of variation within each group of data? Is the variance similar between the groups that are being statistically compared?	Yes	Figures, Materials and Methods

Sample definition and in-laboratory replication	Information included in the manuscript?	In which section is the information available? (Reagents and Tools Table, Materials and Methods, Figures, Data Availability Section)
In the figure legends: state number of times the experiment was **replicated** in laboratory.	Yes	Figures
In the figure legends: define whether data describe **technical or biological replicates**.	Yes	Figures

Ethics

Ethics	Information included in the manuscript?	In which section is the information available? (Reagents and Tools Table, Materials and Methods, Figures, Data Availability Section)
Studies involving **human participants**: State details of **authority granting ethics approval** (IRB or equivalent committee(s), provide reference number for approval.	Not Applicable	
Studies involving **human participants**: Include a statement confirming that **informed consent** was obtained from all subjects and that the experiments conformed to the principles set out in the WMA Declaration of Helsinki and the Department of Health and Human Services Belmont Report.	Not Applicable	
Studies involving **human participants: For** publication of **patient photos**, include a statement confirming that consent to publish was obtained.	Not Applicable	
Studies involving experimental **animals**: State details of **authority granting ethics approval** (IRB or equivalent committee(s), provide reference number for approval. Include a statement of compliance with ethical regulations.	Not Applicable	
Studies involving **specimen and field samples:** State if relevant **permits** obtained, provide details of authority approving study; if none were required, explain why.	Not Applicable	

Dual Use Research of Concern (DURC)	Information included in the manuscript?	In which section is the information available? (Reagents and Tools Table, Materials and Methods, Figures, Data Availability Section)
Could your study fall under dual use research restrictions? Please check biosecurity documents and list of **select agents and toxins** (CDC): https://www.selectagents.gov/sat/list.htm	Not Applicable	
If you used a select agent, is the security level of the lab appropriate and reported in the manuscript?	Not Applicable	
If a study is subject to dual use research of concern regulations, is the name of the **authority granting approval and reference number** for the regulatory approval provided in the manuscript?	Not Applicable	

Reporting

The MDAR framework recommends adoption of discipline-specific guidelines, established and endorsed through community initiatives. Journals have their own policy about requiring specific guidelines and recommendations to complement MDAR.

Adherence to community standards	Information included in the manuscript?	In which section is the information available? (Reagents and Tools Table, Materials and Methods, Figures, Data Availability Section)
State if relevant guidelines or checklists (e.g., **ICMJE, MIBBI, ARRIVE, PRISMA**) have been followed or provided.	Not Applicable	
For **tumor marker prognostic studies,** we recommend that you follow the **REMARK** reporting guidelines (see link list at top right). See author guidelines, under 'Reporting Guidelines'. Please confirm you have followed these guidelines.	Not Applicable	
For **phase II and III randomized controlled trials**, please refer to the **CONSORT** flow diagram (see link list at top right) and submit the CONSORT checklist (see link list at top right) with your submission. See author guidelines, under 'Reporting Guidelines'. Please confirm you have submitted this list.	Not Applicable	

Data Availability

Data availability	Information included in the manuscript?	In which section is the information available? (Reagents and Tools Table, Materials and Methods, Figures, Data Availability Section)
Have **primary datasets** been deposited according to the journal's guidelines (see 'Data Deposition' section) and the respective accession numbers provided in the Data Availability Section?	Not Applicable	
Were **human clinical and genomic datasets** deposited in a public access-controlled repository in accordance to ethical obligations to the patients and to the applicable consent agreement?	Not Applicable	
Are **computational models** that are central and integral to a study available without restrictions in a machine-readable form? Were the relevant accession numbers or links provided?	Yes	Data Availability Section, Materials and Methods
If publicly available data were reused, provide the respective **data citations in the reference list**.	Not Applicable	
