# Supplementary material for: Dynamics of bicoid mRNA localisation and translation dictate morphogen gradient formation
Source: EMBO J. 2026 Oct 10;45(21):7374–404. doi: 10.1038/s44318-026-00927-5 (PMC13728150; doi:10.1038/s44318-026-00927-5)
Supplement: Supplementary file 3 [file 44318_2026_927_MOESM4_ESM.pdf]

Expanded View Figures

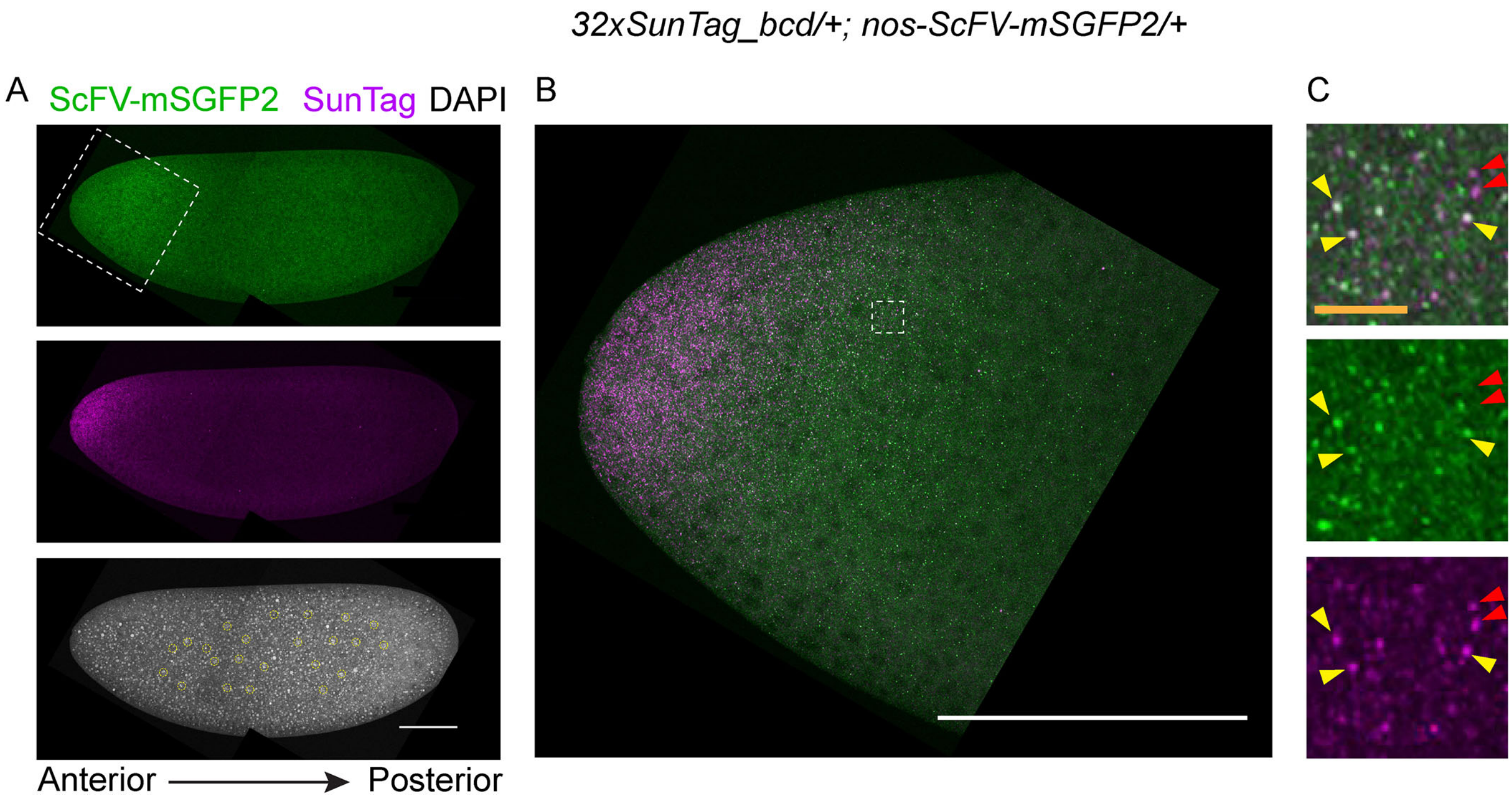

Figure EV1. (Related to Fig. 1): Detection of *bcd* translation using nos-ScFv-mSGFP2.

(**A**) Fixed n.c.4 *32xSunTag bcd/nos-ScFv-mSGFP2* embryos detecting colocalised and non-colocalised ScFv to *bcd*. (Top) ScFv-mSGFP2 (Green), (middle) *bcd* (Magenta), and (lower) Dapi (Grey). (**B**) Magnification of the anterior domain from (**A**). (**C**) Further magnified imaged showing the translating (yellow arrowheads) and non-translating (red arrowheads) *bcd* particles. Scale bars: (**A**) = 100 µm; (**B**) = 100 µm; (**C**) = 5 µm.

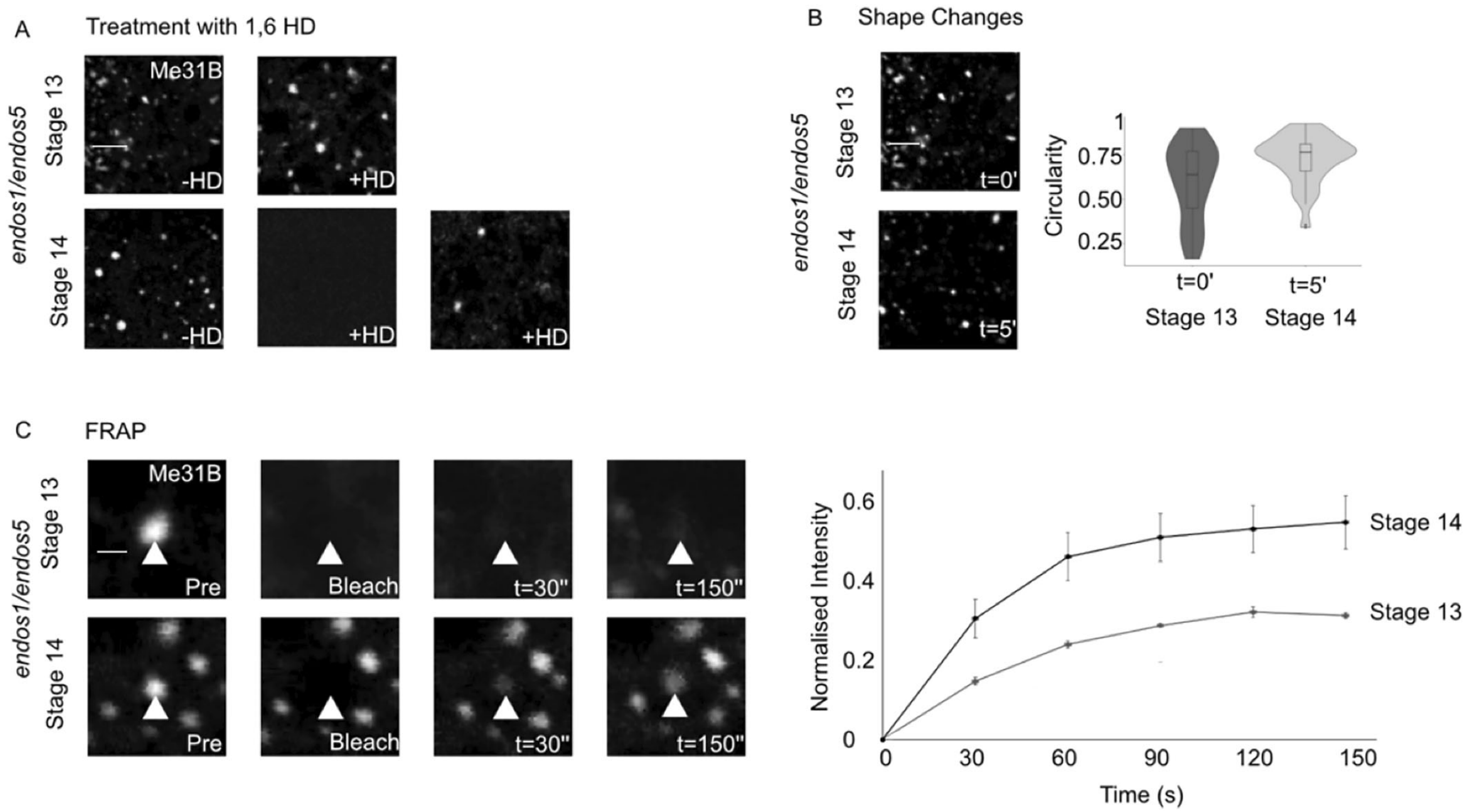

Figure EV2. (Related to Fig. 2): P bodies in Endos mutant are less viscous after oocyte maturation.

(**A**) Treatment of Endos mutant oocytes with 5% 1,6 hexanediol fails to disperse stage 13 oocyte P bodies within 20 min. The same treatment in stage 14 oocytes leads to almost complete P body dispersal consistent with a more liquid-like nature. There are no particles in 70% of the embryos (left 5% 1,6 hexanediol stage 14 panel), and 30% show reduced particles (right 5% 1,6 hexanediol stage 14 panel). $N = 10$ ooyctes for each stage. (**B**) Live imaging of Endos mutant oocytes during maturation followed by analysis of P body shape as measured by circularity. Wilcoxon signed-rank test, ** $p<0.01$, $N = 3$ ooyctes for each stage. Appendix Figure S2A and S2B, stage 13 panels are the same. (**C**) Fluorescence Recovery After Photobleaching (FRAP) of P bodies in Endos backgrounds, shows minimal recovery in stage 13 oocytes. After oocyte maturation there is a higher recovery of fluorescence intensity in stage 14 oocytes, suggesting the condensates are less viscous as they exhibit more exchange with the cytoplasm. $N = 5$ ooyctes for each stage. Scale bars: (**A**, **B**) = 5 µm; (**C**) = 2 µm.

© The Author(s)

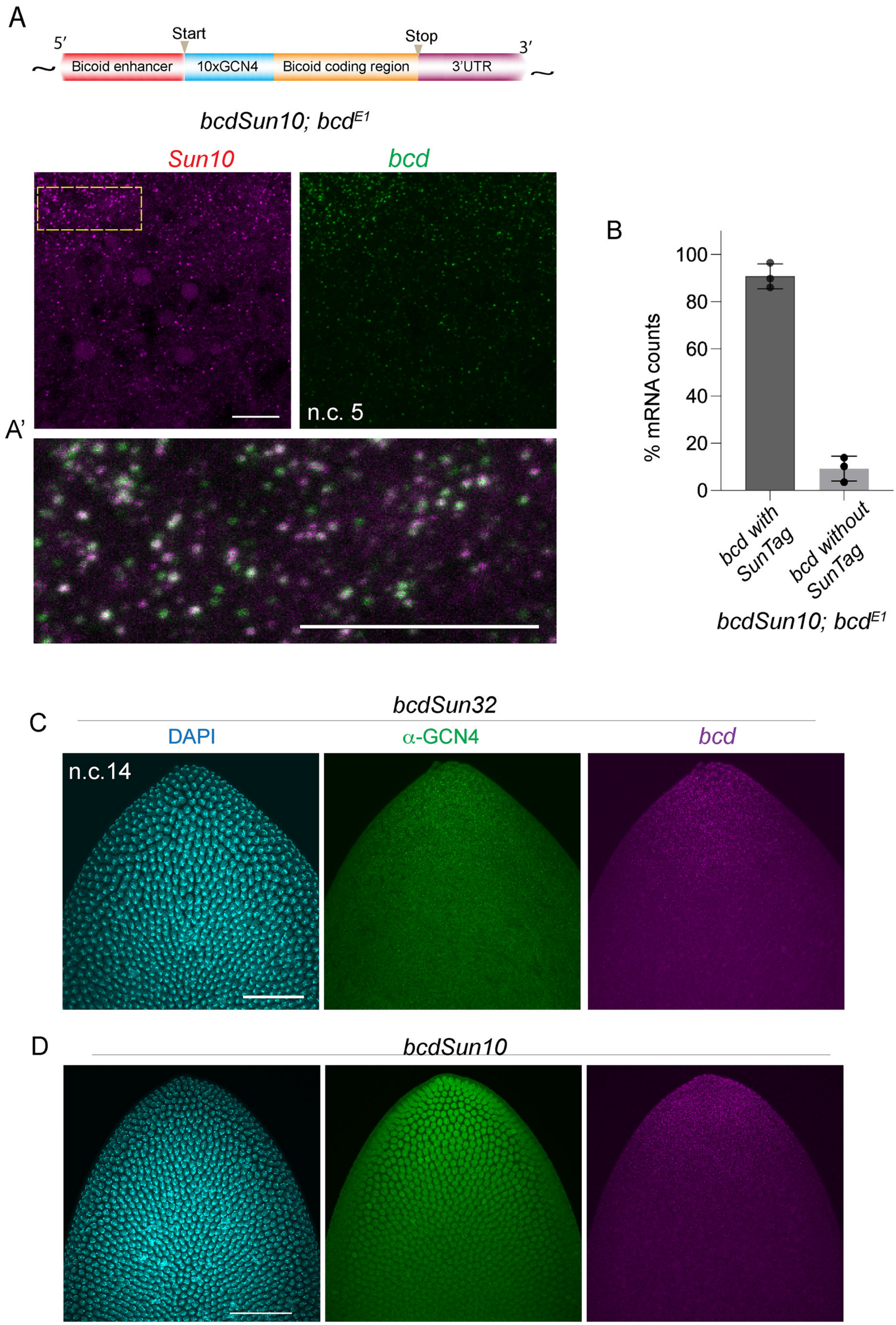
A
5′
Start
Stop
3′
Bicoid enhancer
10xGCN4
Bicoid coding region
3′UTR
bcdSun10; bcdE1
Sun10
bcd
n.c. 5
A'
B
100
80
60
40
20
0
% mRNA counts
bcd with SunTag
bcd without SunTag
bcdSun10; bcdE1
C
bcdSun32
DAPI
α-GCN4
bcd
n.c.14
D
bcdSun10

Figure EV3. (Related to Fig. 3): *bcdSun10* rescues *bcd* null mutant.

(**A**,**A′**) (Top) Cartoon of *bcdSun10* construct. smFISH of SunTag (magenta) and *bcd* (green) regions of *bcdSun10* mRNA localised in the anterior domain of the early blastoderm of homozygous bcd^{E1} mutant background. Yellow box magnified in (**A′**), showing the highly resolved *bcdSun10* mRNA particles. (**B**) Percentage of colocalisation of *bcd* and SunTag regions in the total *bcd* counts in the embryo's anterior pole (3 embryos). Error bars are SD. (**C**, **D**) Immuno FISH images of n.c.14 embryo. Embryos from *bcdSun32* (**C**) and *bcdSun10* (**D**) embryos are shown in upper and lower panels, respectively (see Methods section for details). Scale bars: (**A**, **A′**) = 10 µm; (**C**, **D**) = 50 µm.

 © The Author(s)

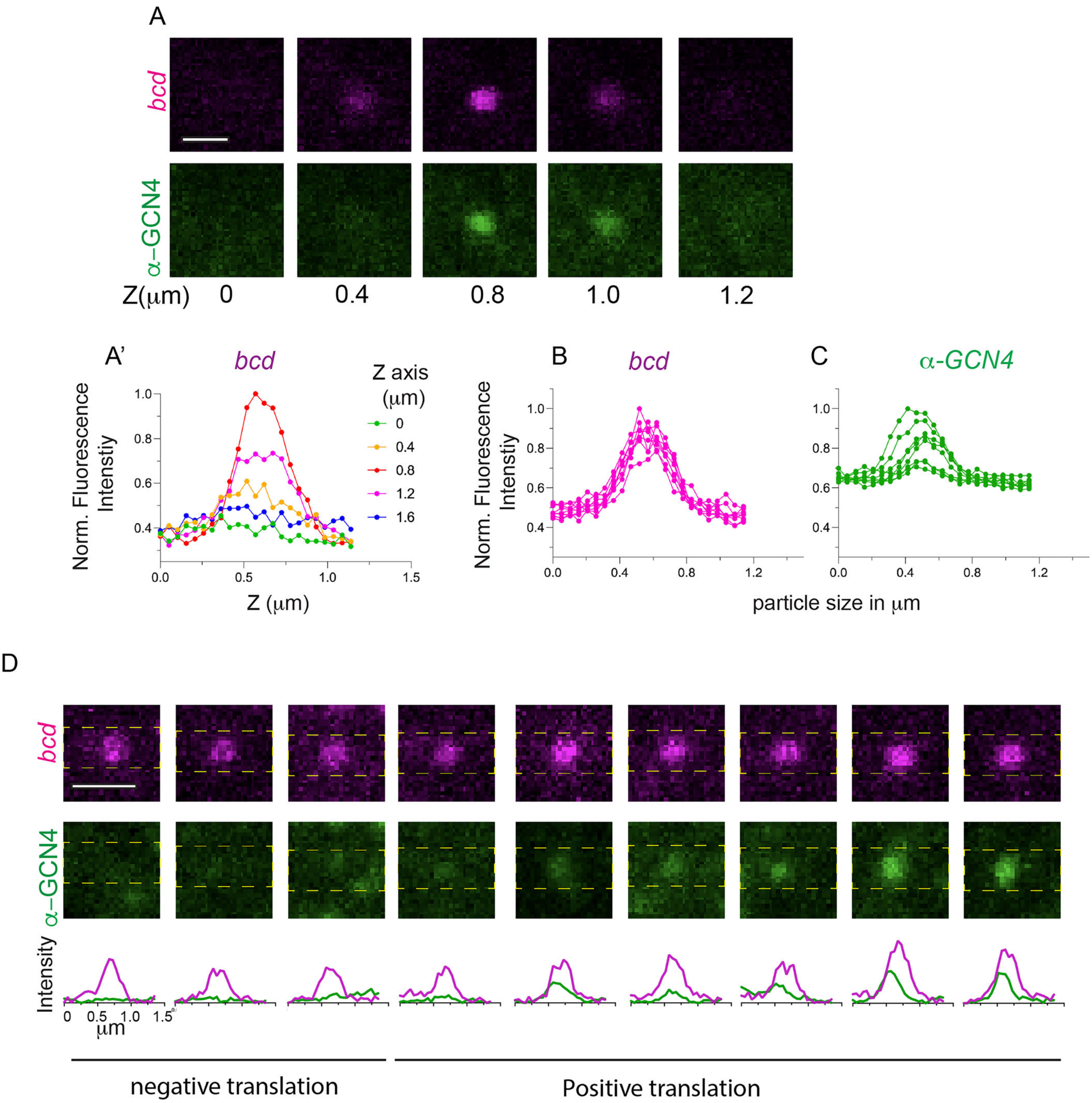

Figure EV4. (Related to Fig. 3): Quantification of translating smFISH *bcd*.

(**A**) Z-slices of translating *bcd* (*bcd* (magenta), anti-GCN4 (Green)) from a *bcdSun10;bcd*^{E1} embryo at n.c.3. The *bcd* intensity from z-slices in (**A**) is captured in (**A'**). (**B–D**) Plot showing maximum intensity of multiple *bcd* particles superimposed to show the overall spot size (Magenta, **B**) and variations in the intensity profile of SunTag ((**C**), green). We find mean width of *bcd* spots is 0.7–0.8 μm. The intensity variations of GCN4 are shown in the bottom panel (**D**). Translating and non-translating *bcd* are classified based on the thresholded SunTag signal. Scale bar: (**A**, **D**) = 1 μm.

© The Author(s)

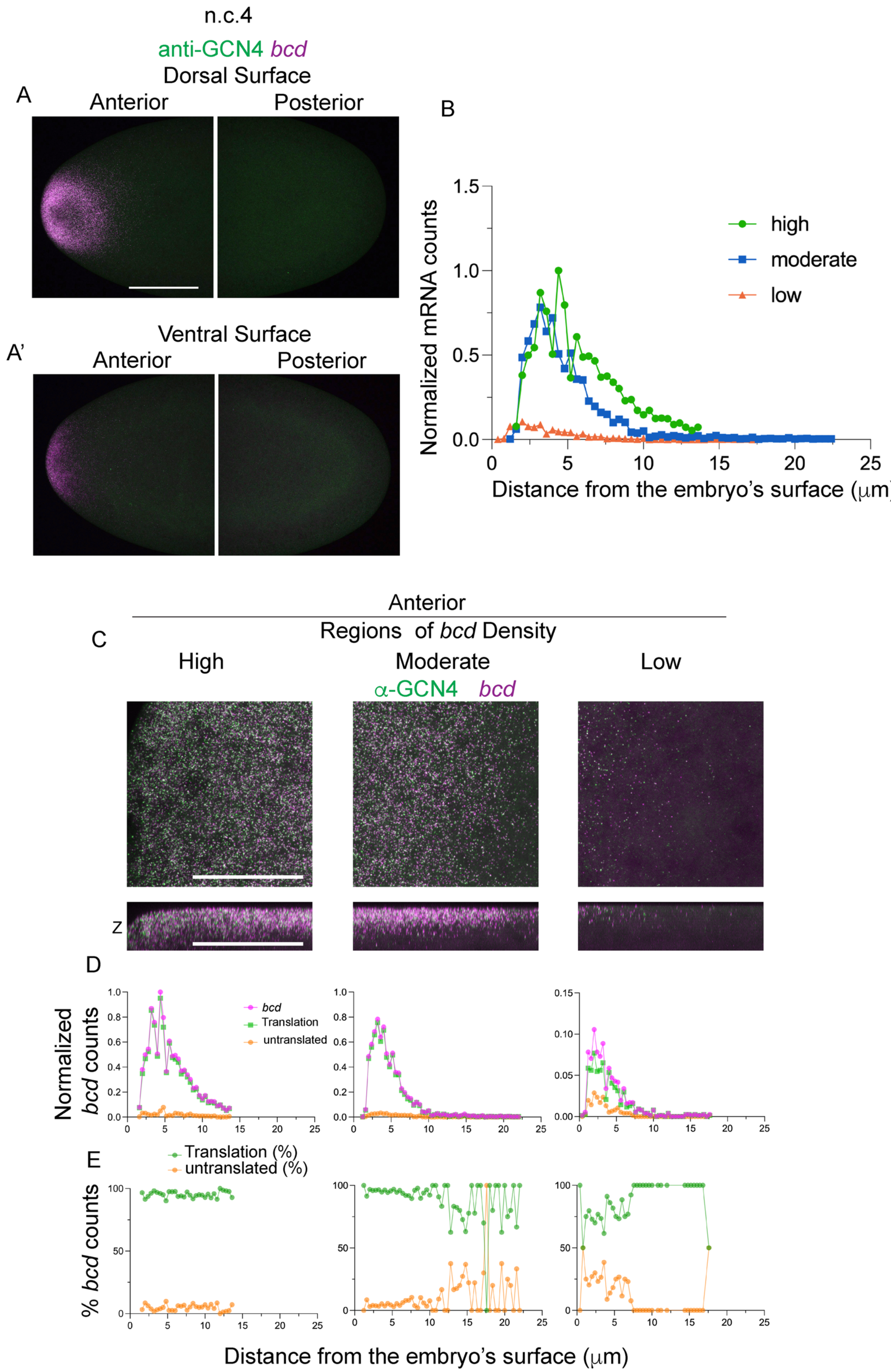

n.c.4
anti-GCN4 *bcd*
Dorsal Surface
A
Anterior
Posterior
A'
Ventral Surface
Anterior
Posterior
B
Normalized mRNA counts
high
moderate
low
Distance from the embryo's surface (μm)
C
Anterior
Regions of *bcd* Density
High
Moderate
Low
α-GCN4 *bcd*
Z
D
Normalized *bcd* counts
bcd
Translation
untranslated
E
Translation (%)
untranslated (%)
% *bcd* counts
Distance from the embryo's surface (μm)

Figure EV5. (Related to Fig. 3): Quantification of translation in the anterior domain of the embryo.

(**A**,**A′**) Immuno-FISH embryos at n.c.4 for *bcd* (magenta) and anti-GCN4 (Green) on the dorsal surface (**A**) and ventral surface (**A′**). The dorsal surface was reproduced here from Fig. 3A,A′, to compare it with the ventral surface. (**B**) Plot showing the distribution of *bcdSun10* counts from the surface-to-depth of the embryo at n.c.4 and at the anterior domains where the mRNA are of high, moderate and low density. Counts are normalised to the total number of mRNA counts in the most anterior domain. (**C**) Maximum intensity projections of Z-slices and Z-cross sections at high, moderate and low dense *bcd* regions as quantified in (**B**). (**D**, **E**) Normalised *bcdSun10* counts (**D**) and their percentages (**E**) undergoing translation at different positions from the embryo's cortical surface (2 embryos analysed). Counts are normalised to the total number of mRNA counts in the most anterior domain. Scale bar: (**A**) = 100 µm, (**C**) = 50 µm.

© The Author(s)

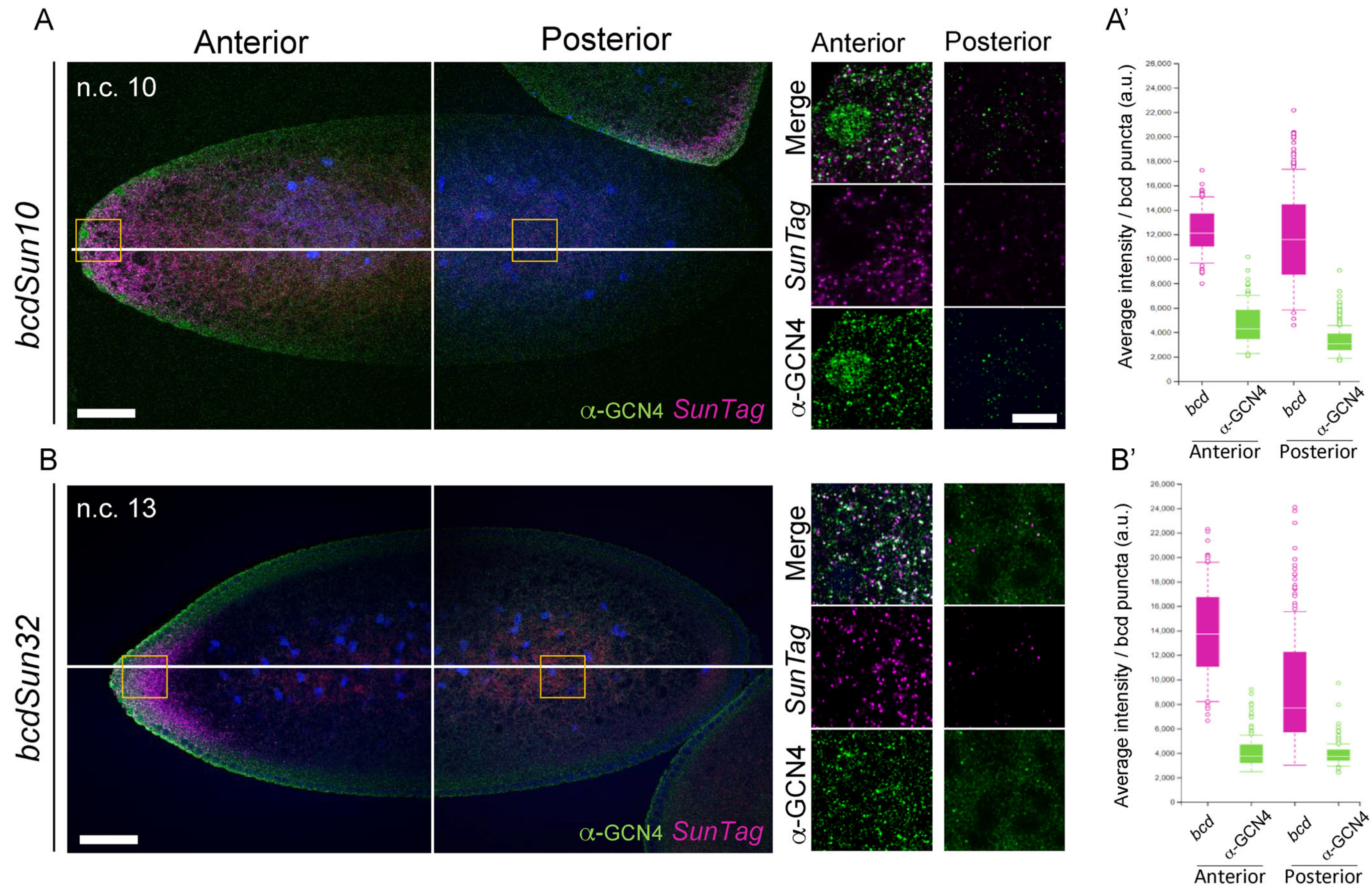

Figure EV6. (Related to Fig. 4): quantification of translation intensity of *bcd* in the anterior and posterior domains.

(**A–B′**) Projected Z stacks of the anterior and posterior domains embryos expressing *bcdSun*32 (**A**) and *bcdSun*10 (**B**) transgenes. *Bcd* reporters and translation spots were detected by Immuno-FISH using Suntag probes and anti-GCN4 shown in magenta and green, respectively. Quantification of the single molecule average intensity for bcd-reporters FISH and GCN4 IF signals in anterior and posterior domains (**A**′, **B**′) (right panel).

 © The Author(s)

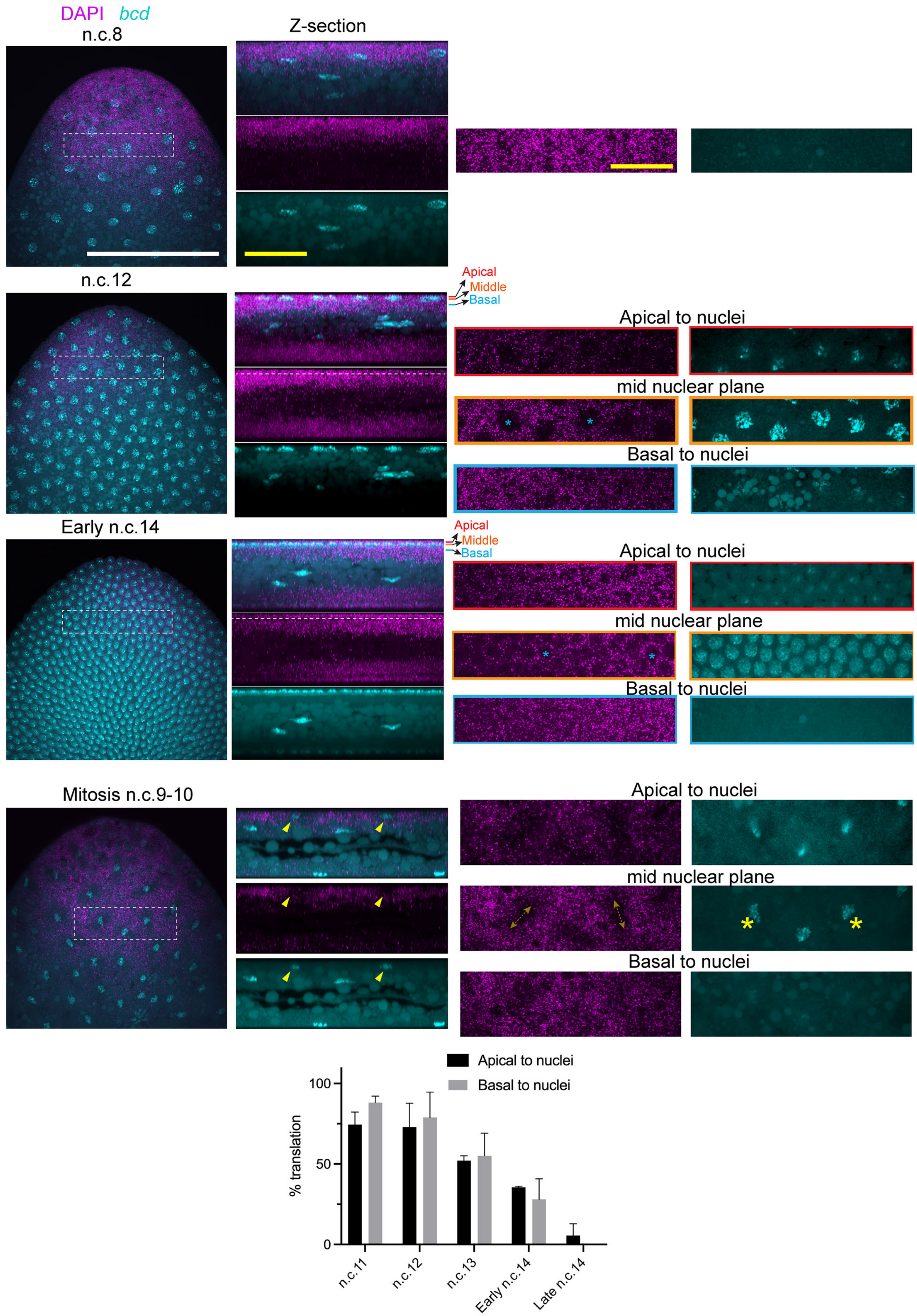
DAPI bcd
n.c.8
Z-section
n.c.12
Apical
Middle
Basal
Apical to nuclei
mid nuclear plane
Basal to nuclei
Early n.c.14
Apical
Middle
Basal
Apical to nuclei
mid nuclear plane
Basal to nuclei
Mitosis n.c.9-10
Apical to nuclei
mid nuclear plane
Basal to nuclei
Apical to nuclei
Basal to nuclei
% translation
100
50
0
n.c.11
n.c.12
n.c.13
Early n.c.14
Late n.c.14

Figure EV7. (Related to Figs. 4 and 5): Redistribution of *bcd* mRNA upon cortical migration and division of blastoderm nuclei.

Left panel: Anterior domain of flattened embryos staged at pre-cortical nuclear migration stages (n.c.8), post cortical nuclear migration stages at n.c.12 and early n.c.14, and at n.c.9-10 mitosis stages stained for *bcd*(magenta) and Dapi (cyan). Middle panel: Z-section of a magnified region (broken box) of the aforementioned staged embryos showing *bcd* distribution across the depth. Broken white lines in the *bcd* single channel of n.c.12 and n.c.14 segregates nuclear boundary. Yellow arrowheads indicate mitotic nuclei. Right panel: X–Y planes of the magnified region in the left panel. Apical, mid, and basal nuclear planes are shown for n.c.12, 14 and mitosis stages. Cyan stars indicate nuclear regions. Yellow double arrowheads indicate the direction of the nuclear division. Yellow stars indicate mitotic nuclei. Bottom: Barplot showing variation in *bcd* translation localised apical and basal to the cortical nuclei. $N = 3$ embryos per nuclear cycle. Scale bars: left panel = 100 µm; middle panel (yellow) = 50 µm; right panel (yellow) = 50 µm.

 © The Author(s)

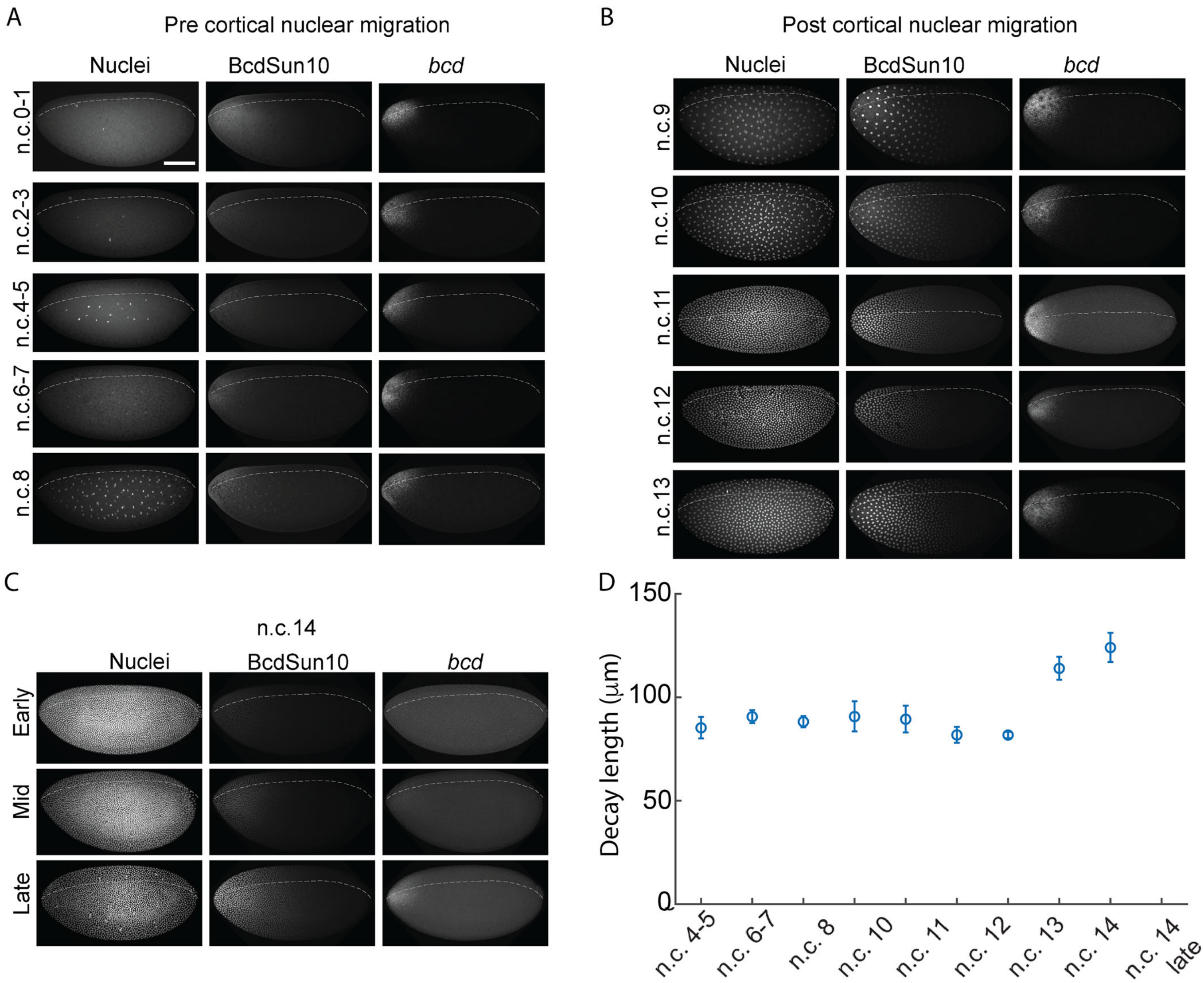

Figure EV8. (Related to Fig. 5): The *bcdSun10* gradient is formed by spread from the translation domain.

(**A**–**C**) *bcdSun10;bcd*^{E1} embryos at different stages, showing the evolution of the SunTag-Bcd gradient (middle panel) in pre-cortical (**A**), post-cortical nuclear migration (**B**) and n.c.14 (**C**) with respect to the *bcd* distribution (right panel). The embryos are stained with anti-GCN4 antibody (Bcd-SunTag), *bcd* (mRNA) and DAPI (nuclei). Embryos classified based on the nuclear counts and internuclear distances from the DAPI staining. Broken lines indicate the mid-dorsal lines of the embryo. Scale bar: 50 μm. (**D**) Quantification of the decay length of the BcdSun10 gradient between 100 μm to 350 μm (from data presented in Fig. 5). Equation of fit: $[Bcd] = [Bcd]_0 e^{-x/\lambda}$, where λ is the measured decay length. Error bars 95% confidence intervals. Increase in n.c.14 due to decreasing Bcd concentration, leading to flatter profile.

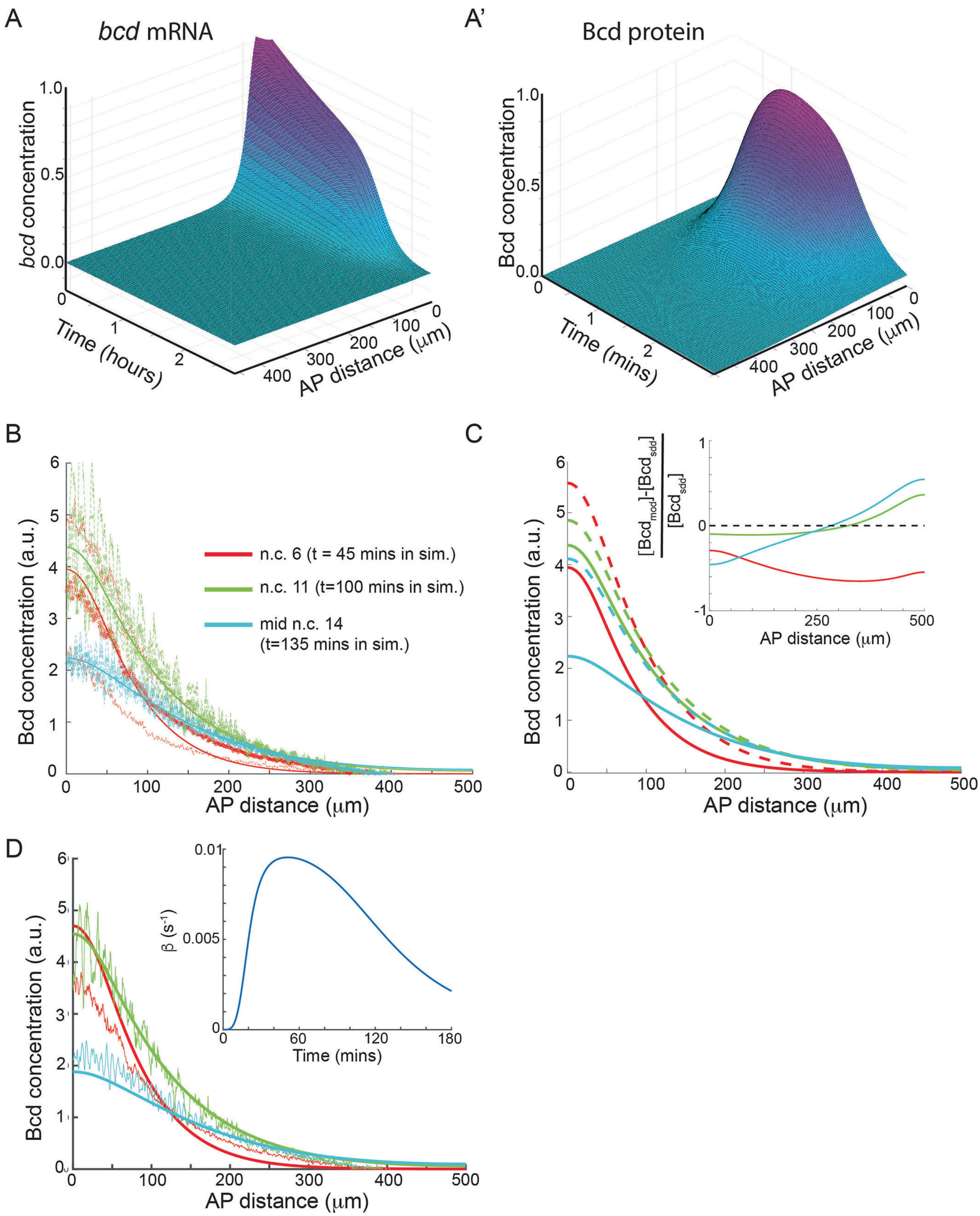
A
bcd mRNA
bcd concentration
1.0
0.5
0.0
Time (hours)
0
1
2
AP distance (μm)
0
100
200
300
400
A'
Bcd protein
Bcd concentration
1.0
0.5
0.0
Time (mins)
0
1
2
AP distance (μm)
0
100
200
300
400
B
Bcd concentration (a.u.)
AP distance (μm)
n.c. 6 (t = 45 mins in sim.)
n.c. 11 (t=100 mins in sim.)
mid n.c. 14
(t=135 mins in sim.)
0
100
200
300
400
500
C
Bcd concentration (a.u.)
AP distance (μm)
[Bcd_mod]-[Bcd_sdd] / [Bcd_sdd]
1
0
-1
0
250
500
AP distance (μm)
D
Bcd concentration (a.u.)
AP distance (μm)
β (s-1)
0.01
0.005
0
Time (mins)
0
60
120
180

© The Author(s)

Figure EV9. (Related to Fig. 7): Modified SDD model of Bcd gradient formation.

(**A**) Normalised concentration (by maximum value) in space and time for *bcd* (**A**) and Bcd protein (**A**′) from the modified SDD model. (**B**) Same as Fig. 7D but showing the standard error on the experimental concentration measurements (dashed lines). (**C**) Comparison of SDD (dashed) and modified-SDD (solid) models, with colour scheme same as (**B**). Inset shows the relative difference in the two models. Here, the SDD model is taken to have constant diffusion coefficient, mRNA degradation, and translation rates (Table S5). (**D**) Modified SDD model with time varying β parameter, as shown in inset. Colour coding same as in (**B**).

© The Author(s)
