# Supplementary material for: Dynamics of bicoid mRNA localisation and translation dictate morphogen gradient formation
Source: EMBO J. 2026 Oct 10;45(21):7374–404. doi: 10.1038/s44318-026-00927-5 (PMC13728150; doi:10.1038/s44318-026-00927-5)
Supplement: Supplementary file 4 [file 44318_2026_927_MOESM3_ESM.zip › Movie EV1.rtf]

Movie EV1: Time lapse video of the developing blastoderm of the Drosophila embryo of genotype nos-scFv-mNeonGreen/10xSunTag-bcd (upper panel) and the control, nos-scFv-mNeonGreen (lower panel). 

Imaging conditions : 
Upper panel: image size: 2304x2304 pixels, 4.66mm/pixel, 16 bits, Z-2mm, thickness: 54mm, time frames: 0.5min/frame. 

Lower panel: Image size: 2304x2304 pixels, 4.66mm/pixel, 16 bits, Z-2mm, thickness: 54mm, time frames: 0.5min/frame. Scale Bar 100mm.
